# Effect of Structural Differences in Naringenin, Prenylated Naringenin, and Their Derivatives on the Anti-Influenza Virus Activity and Cellular Uptake of Their Flavanones

**DOI:** 10.3390/ph15121480

**Published:** 2022-11-28

**Authors:** Ryosuke Morimoto, Chiaki Matsubara, Akari Hanada, Yuta Omoe, Tokutaro Ogata, Yuji Isegawa

**Affiliations:** 1 Department of Food Sciences and Nutrition, Mukogawa Women’s University, Nishinomiya 663-8558, Hyogo, Japan; 2 Faculty of Pharmaceutical Sciences, Hokuriku University, Kanazawa, 920-1181, Ishikawa, Japan

**Keywords:** naringenin, 8-prenylnaringenin, 6-prenylnaringenin, flavonoid, flavanone, *Citrullus lanatus*

## Abstract

Vaccines and antiviral drugs are widely used to treat influenza infection. However, they cannot rapidly respond to drug-resistant viruses. Therefore, new anti-influenza virus strategies are required. Naringenin is a flavonoid with potential for new antiviral strategies. In this study, we evaluated the antiviral effects of naringenin derivatives and examined the relationship between their cellular uptake and antiviral effects. Madin–Darby canine kidney (MDCK) cells were infected with the A/PR/8/34 strain and exposed to the compound-containing medium for 24 h. The amount of virus in the supernatant was calculated using focus-forming reduction assay. Antiviral activity was evaluated using IC_50_ and CC_50_ values. Cells were exposed to a constant concentration of naringenin or prenylated naringenin, and intracellular uptake and distribution were evaluated using a fluorescence microscope. Prenylated naringenin showed strong anti-influenza virus effects, and the amount of intracellular uptake was revealed by the strong intracellular fluorescence. In addition, intracellular distribution differed depending on the position of the prenyl group. The steric factor of naringenin is deeply involved in influenza A virus activity, and prenyl groups are desirable. Furthermore, the prenyl group affects cellular affinity, and the uptake mechanism differs depending on its position. These results provide important information on antiviral strategies.

## 1. Introduction

The influenza virus is an RNA virus that belongs to the family Orthomicroviridae, which mainly causes infections in mammals. While the spread of COVID-19 appears to have reduced influenza virus infections [1], co-infections in animals [2] and humans [3] have been reported. Influenza virus has caused pandemics, with many reported deaths [4,5]. Due to its susceptibility to antigenic variation [6], influenza virus may cause a pandemic in the future.

Several agents have been developed for the treatment of influenza. M2 channel blockers (amantadine and rimantadine), neuraminidase inhibitors (oseltamivir, zanamivir, peramivir, and laninamivir), viral RNA polymerase inhibitor (favipiravir), and cap-dependent endonuclease inhibitor (baloxavir marboxil) are known. Antiviral drugs, such as oseltamivir, act directly on the virus multiplication mechanism. However, successive reports of drug-resistant strains or potential have been reported [7,8]. Lampejo warned that new antiviral strategies should be con-sidered when preparing for a pandemic [9].

Natural plant compounds are important sources for future drug development. Flavonoids are plant secondary metabolites classified as polyphenols. Naringenin is classified as a flavanone and is present in plants such as citrus fruits and others [10,11,12]. Flavanone group compounds and derivatives have broad-spectrum biological activity [13,14,15,16,17,18] and have attracted attention as antiviral agents [19]. Recently, the physiological functions, including biological and pharmacological effects of prenylated compounds have been reported [19,20,21,22,23,24]. We previously reported that 8-prenylnaringenin (8-PN) isolated from wild watermelon (*Citrullus lanatus* var. citroides (L.H. Bailey)) exhibits high antiviral activity [25]. Therefore, we believe that plants rich in prenylated compounds have the potential to be important sources for antiviral drug development strategies. For example, xanthohumol, 8-PN, and 6-prenylnaringenin (6-PN), which are produced by hop (*Humulus lupulus* L.) [26,27].

Additionally, we reported that the antiviral activity of flavonoids depends on the type and structure of the modifying group, suggesting that this may be due to the differences in substituent position, steric effect, and coordination effect of the compound [28]. When considering anti-influenza virus strategies, it is important to study the effects of these chemical structures and the pharmacokinetics of tissues and cells. Mukai et al. reported the cellular uptake of flavonols, quercetin, and 8-prenyl quercetin [29]. However, the cellular distribution of prenylated naringenin has not been fully investigated. Therefore, in this paper, to investigate the chemical and pharmacological properties related to antiviral activity, derivatives with several substituents with different properties were synthesized to elucidate the relationship between the cellular uptake and intracellular distribution of prenylated naringenin and its antiviral activity.

## 2. Results

To investigate the viral effects of chemical structure, we synthesized compounds based on 8-PN and 6-PN. The chemical structures of the compounds were confirmed using ^1^H NMR (500 MHz) and ^13^C NMR (125 MHz). The synthesized compound information is shown in Figures S1–S32 and Supplementary Materials. This was within the range of no cytotoxicity.

### 2.1. Anti-Influenza Activity of Prenylated Naringenin (8-PN, 6-PN) and Other Derivatives (***1a***–***2d***)

First, we evaluated four compounds (naringenin, naringenin-4′,7′-diacetate, 8-PN, and 6-PN). The IC_50_ value of naringenin was 290 μM. Additionally, naringenin-4′,7-diacetate markedly lost its anti-influenza activity. Prenylated naringenin, 8-PN and 6-PN, showed strong activity with IC_50_ values of 24 and 38 μM, respectively. Compared to naringenin, prenylation of the 8- or 6-position dramatically increases anti-influenza virus activity. However, no significant difference in activity was observed between the 8- and 6-position substituents (Table 1).

### 2.2. Coordination Effect of Modifying Groups on the Naringenin Skeleton 8-Position

Next, we evaluated the effect of unshared electron pairs **1a**, **1b**, and **1e**. Compound **1a**, in which an epoxide group was introduced into the molecular structure, showed activity at 59 μM, but was less active than 8-PN (Table 1). The activities of **1b** and **1e** were lost. In addition, to evaluate the coordination ability of the double bonds in the molecular structure at the 8-position, additional compounds were synthesized. The IC_50_ values of compounds **1c** and **1d**, which are saturated hydrocarbon side chains, were 42 and 61 μM, respectively.

### 2.3. Stereoscopic Effect of Modifying Groups on the Naringenin Skeleton

Previous studies have also suggested the effect of steric factors on the anti-influenza virus effect [28]. Subsequently, to investigate the steric effect, substituents with different steric effects (allyl groups: **2a**, isopentyl group: **2b**, cycloalkanes: **2c** and **2d**) were introduced. Compounds **2a** and **2b** showed weaker activities than 6-PN. The activities of **2c** and **2d** were not detected. In addition, isopentyl derivatives (**1d** and **2b)**, showed lower activity than prenylated naringenins (Table 1).

### 2.4. Uptake of Naringenin and Prenylated Naringenin into Madin–Darby Canine Kidney (MDCK) Cells

**Figure 1:**
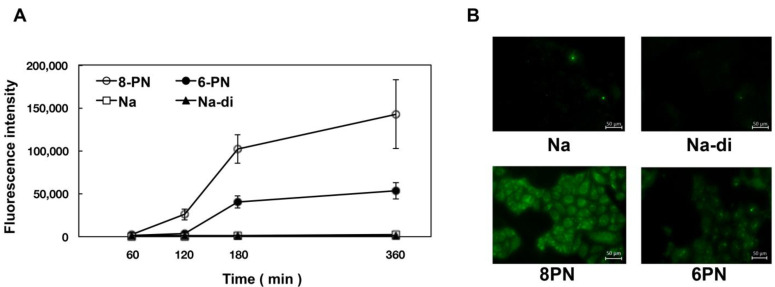
Time course and fluorescence images of prenylated naringenin and other derivatives. (**A**) Fluorescence intensity of 8-PN, 6-PN, naringenin, and naringenin-4′,7′-diacetate. (**B**) Fluorescence images.

**Figure 2:**
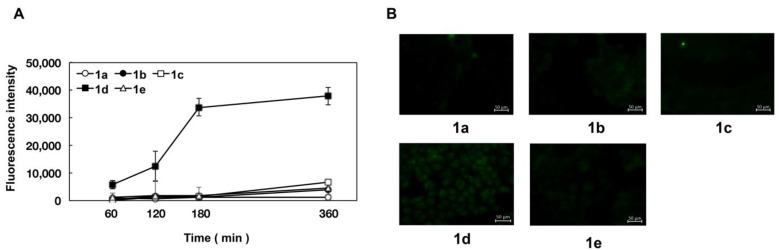
Time course and fluorescence images of 8-position naringenin derivatives. (**A**) Fluorescence intensity of 8-position naringenin derivatives. (**B**) Fluorescence images.

**Figure 3:**
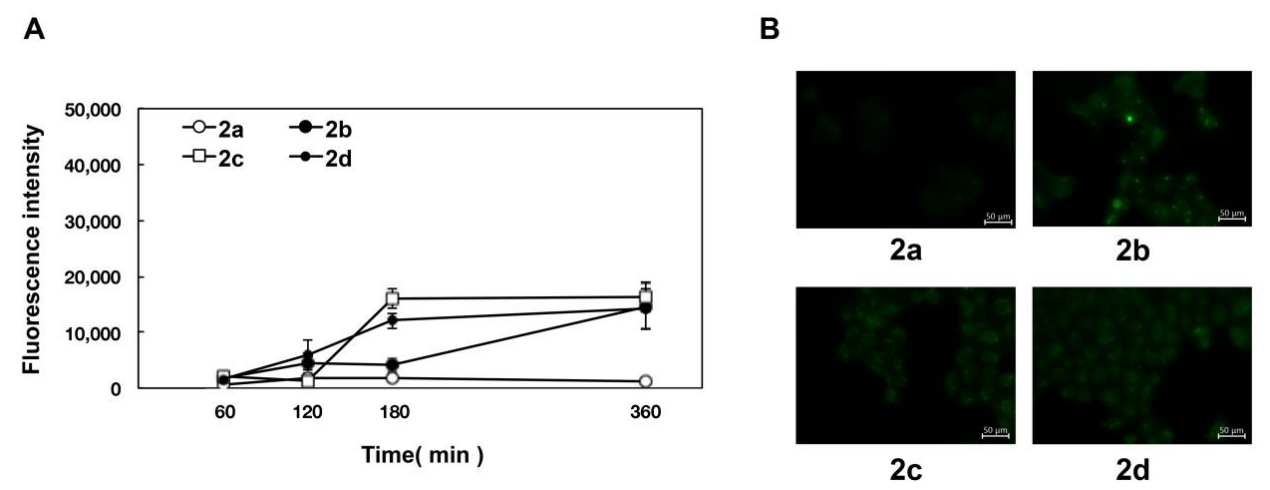
Time course and fluorescence images of 6-position naringenin derivatives. (**A**) Fluorescence intensity of 6-position naringenin derivatives. (**B**) Fluorescence images. Madin–Darby canine kidney (MDCK) cells were treated with 30 μM of the compound in phenol red-free medium. We measured the mean fluorescence intensity at each time point (60, 120, 180, and 360 min), which was calculated from approximately 20 cells per time point. (**B**) Images show cells after 180 min of compound treatment. Scale bars = 50 μm.

From the obtained results (Table 1), it was found that the substituents in the flavanone skeleton greatly affected antiviral activity. To confirm the relationship between compound uptake and antiviral activity, we evaluated intracellular uptake by fluorescence microscopy. The experiment was conducted by modifying the method described by Wolf et al. [30]. Figure 1, Figure 2 and Figure 3 summarize the fluorescence intensity of each compound per 20 cells at each time point. This fluorescence intensity transition was also confirmed by the images. Images obtained after 180 min are shown in Figure 1B, Figure 2B and Figure 3B. Several compounds increased the fluorescence intensity after 120 min, reaching plateaus between 180 and 360 min. Naringenin, naringenin-4′,7′-diacetate, 8-position derivatives, and 6-position derivatives showed low fluorescence intensity, whereas 8-PN and 6-PN showed high fluorescence intensity (Figure 1A, Figure 2A and Figure 3A). This fluorescence intensity transition was also confirmed by the images (Figure 1B, Figure 2B and Figure 3B). In addition, the isopentyl group (**1d**) showed stronger fluorescence intensity than the other compounds. The fluorescence intensity at 360 min decreased in the order of 8-PN > 6-PN > **1d** > other compounds.

### 2.5. Intracellular Distribution of Naringenin and Prenylated Naringenin

**Figure 4:**
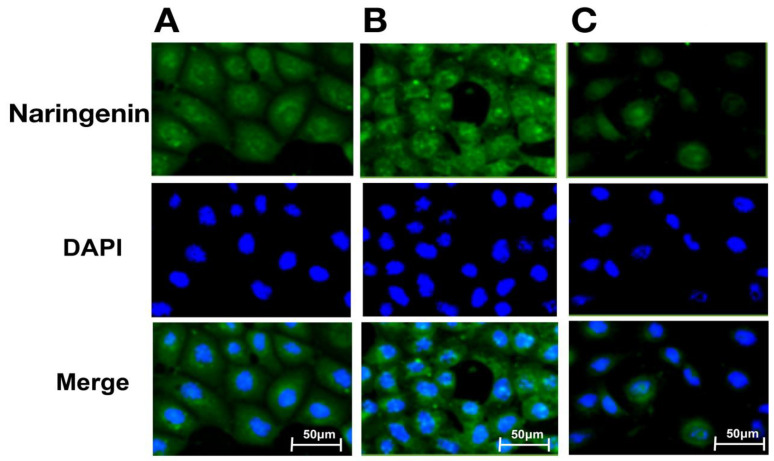
Analysis of prenylated naringenin and naringenin fluorescence distribution by imaging. After removing the medium, cells were treated with 30 μM compound for 180 min and fixed with 4% paraformaldehyde. Fixed cells were stained with DAPI (blue). These images show fluorescence of 8-prenylnaringenin (8-PN; **A**), 6-prenylnaringenin (6-PN; **B**), and naringenin (**C**). Scale bars = 50 μm.

Based on these results, we investigated the intracellular distributions of naringenin and prenylated naringenin. Cells cultured on glass plates were exposed to a concentration of 30 μM for 180 min. The exposed cells were washed with PBS and the compounds were removed from the medium. The cells were then fixed with 4% paraformaldehyde and observed under a fluorescence microscope. At 30 μM, the intracellular prenylated naringenin group showed a remarkable fluorescence pattern (Figure 4). The amount of naringenin taken up by the cells may be small. Moreover, fluorescence was detected for prenylated naringenin. We confirmed that 8-PN was distributed mostly in the cytoplasm and nucleus, while 6-PN tended to localize to the cytoplasmic periphery.

### 2.6. Extracellular Kinetics of Prenylated Naringenin

**Figure 5:**
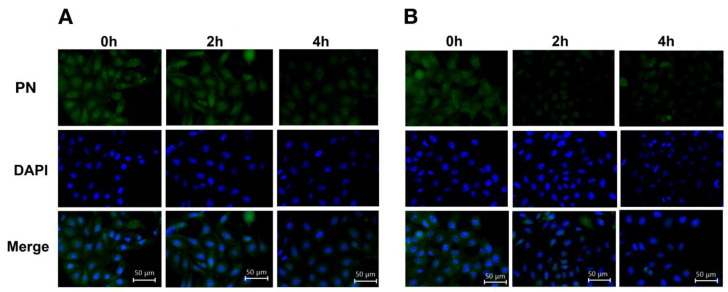
Efflux of prenylated naringenin after intracellular saturation. The amount of the compound in the cells was saturated by exposure for 180 min, and the cells were cultured in Dulbecco’s Modified Eagle Medium (DMEM) for 0, 120, and 240 min. After removing the medium, cells were fixed with 4% paraformaldehyde and stained with DAPI (blue). These images show 8-PN (**A**) and 6-PN (**B**) fluorescence. Scale bars = 50 μm.

**Figure 6:**
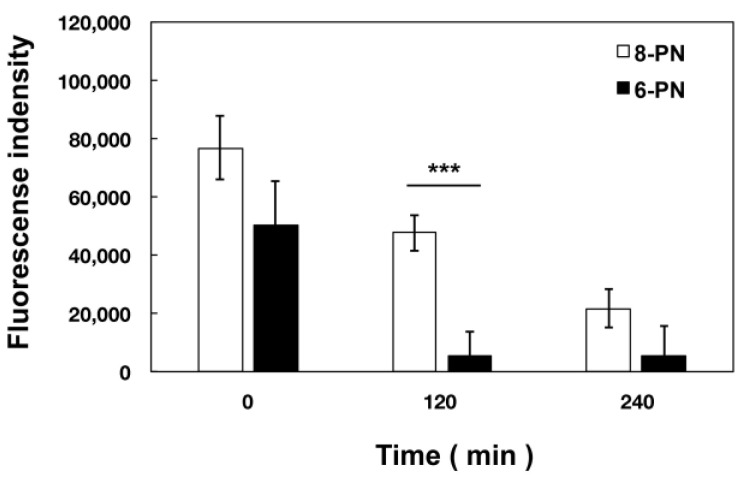
Efflux and fluorescence intensity of intracellular prenylated naringenins. Fluorescence intensity of 8-PN and 6-PN from Figure 5. The standard deviation bars are indicated on each bar graph. *** *p* < 0.001.

Based on our results, we examined the kinetics of intracellular prenylated naringenin in Dulbecco’s Modified Eagle Medium (DMEM) containing 1% fetal bovine serum (FBS). After 180 min of exposure, the cells were cultured in compound-free medium and fluorescence was observed at each time point. Consequently, each prenylated naringenin exhibited different kinetics. Intracellular 8-PN was released from the cell slowly, while 6-PN was released relatively quickly (Figure 5 and Figure 6). The intracellular kinetics changed greatly depending on the position of the prenyl group. After 120 min of culturing in the medium, the fluorescence intensity of 8-PN and 6-PN increased approximately 1.6-fold and 9.4-fold, respectively, compared to that at 0 min (Figure 6).

### 2.7. Growth of Influenza Virus Was Inhibited by Prenylated Naringenin

**Figure 7:**
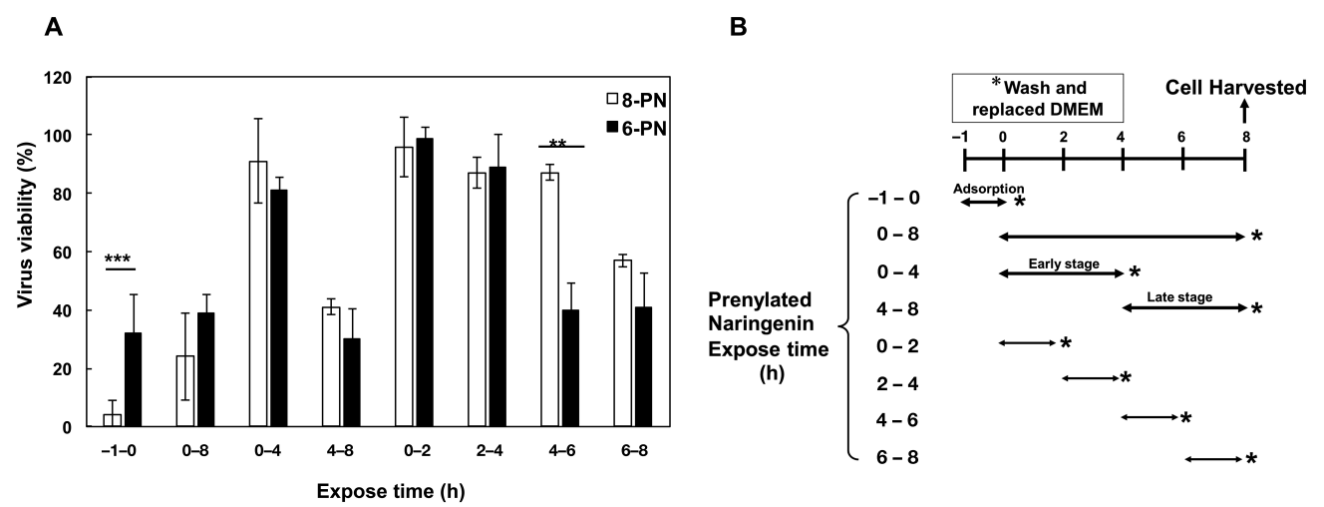
Effect of prenylated naringenin on virus viability. (**A**) 8-PN and 6-PN (30 μM). (**B**) Schedule of the experiment. Cells were infected with influenza virus A/PR/8/34 at a multiplicity of infection of 0.01 in 24-well plates. After infection at each time point, the cells were harvested and viruses were assayed using a focus-forming reduction assay. Each column shows virus viability at each stage of virus growth induced by 8-PN and 6-PN. Data are representative of three independent experiments. Standard deviation bars indicate each bar graph (*** *p* < 0.001, ** *p* < 0.01).

We performed a time-of-addition assay to verify the effect of the prenyl group position on viral growth inhibition. Figure 7A,B show virus viability when exposed to each prenylated naringenin for each time duration. It was commonly inhibited after infection at the late stage (4–8 h, 6–8 h). However, 8-PN and 6-PN showed characteristic inhibition, depending on the position of the prenyl group. When exposed to prenylated naringenin during the virus adsorption period (−1–0 h), 8-PN was confirmed to have a high inhibitory effect. Conversely, 6-PN showed a high inhibition tendency during a 2 h exposure time (4–6 h). In previous studies, inhibitory effects differed depending on the flavonoid backbone [28]. The anti-influenza virus effect depends on the position of the prenyl group, which is a novel observation of the present study.

## 3. Discussion

The antiviral effects of some flavonoid aglycones and their derivatives have been investigated [31,32] and have the potential to inhibit each viral growth stage [33,34,35]. Naringenin has been reported to be effective against various viruses [36,37], but there are few reports on its effectiveness against influenza viruses. We have previously reported the antiviral effects of 8-PN in plants [25]; 8-PN showed stronger activity than naringenin. In addition, we reported that the antiviral effect depends on the structure of the flavonoids [28]. Here, we investigated the anti-influenza effects of naringenin derivatives and their kinetics in cells.

First, we focused on the relationship between the structure and activity of naringenin and its derivatives, including prenylated naringenin. We hypothesized that the structure of the substituents was a factor in viral activity and synthesized 13 compounds with substituents with different properties. Naringenin showed antiviral activity, while naringenin-4′,7′-diacetate lost its activity. We predicted that the diacetyl structure is involved in the expression of activity. However, compound **1c** exhibited antiviral activity at 42 μM. These results suggest that the 8-position substituent, rather than the 4′and 7′ positions, is essential for anti-influenza virus activity. Additionally, the activity was expressed by the isopentyl group (**1c** and **1d**); however, **1b** and **1e** were not active. These results suggested that the dimethyl structure of the carbon chain is involved in the expression of activity. The epoxide group (**1a**) had decreased anti-influenza virus activity. Therefore, substituents (hydrocarbon chains) that do not exhibit coordinating properties in the flavanone skeleton are desirable.

Next, the antiviral activities of the 6-position derivatives were evaluated. Since the activity of 6-allylnaringenin (**2a**) decreased, highlighting the effect of the length of the carbon chain rather than the electronic effect of the substituents, it was inferred that a steric factor was also involved in the 6-position derivatives. The effect of the double-bond site in the substituent is thought to be due to the coordinating property or partial planarity, but no clear factor could be confirmed. However, unsaturated substituents would be desirable, as compounds with double bonds show stronger activity (Table 1). In addition, the **2c** and **2d** results suggest that cyclohexane may be a steric obstacle for activity, and the steric size suitable for the anti-influenza virus effect was the dimethyl structure in the prenyl group, which has a branched structure.

Therefore, 8-PN and 6-PN showed the strongest activities among the synthesized derivatives group, and the prenyl group dramatically affected the anti-influenza virus effect. The results of the 8- and 6-position derivatives suggest that the steric factors of the compounds are deeply involved in anti-influenza virus activity.

In addition, based on the research report by Murota et al. [38], we speculated that the prenyl group has the potential to enhance affinity with cell lipid membranes and increase cellular uptake. To confirm this hypothesis, we exposed cultured cells to naringenin and prenylated naringenin and observed them using fluorescence microscopy. Cellular uptake experiments confirmed the characteristic fluorescence intensity depending on the structure of the compound (Figure 1, Figure 2 and Figure 3). After 180 min of exposure, many compounds showed a plateau in MDCK cells, and **1d**, 8-PN, and 6-PN showed high fluorescence intensities in each derivative group. These results indicate that the prenyl and isopentyl groups at the 8-position enhance the affinity to cells and to the cell membrane or other uptake factors. When further cellular distribution was examined by fluorescence microscopy and compared to naringenin, the distribution of fluorescence was clearly confirmed (Figure 4). Tanaka et al. reported that the cellular uptake of 8-PN in HEK293 cells was higher than that of naringenin [39], and similar results were obtained in MDCK cells. While 8-PN was localized throughout the nucleus and cytoplasm, 6-PN was localized in the periphery of the cytoplasm. This kinetics can be attributed to the in vivo interactions of each compound. Low-molecular-weight compounds such as flavonoids are known to bind to proteins in vivo, and albumin is also used as an indicator of in vivo nutrition [40]. Flavonoids have also been reported to have a high affinity for intracellular proteins [41,42], which is not only due to the affinity of the compound to the cell membrane, but also due to the interaction with intracellular proteins. The fact that the medium contained approximately 20 nM albumin during fluorescence microscopy and about 60 μM at the time of the addition assay may also affect the results. To confirm the kinetics of prenylated naringenin, we prepared cells saturated with the compound. After saturation, the cells were exposed to DMEM containing 1% FBS and fluorescence was observed at each time point. Consequently, the intracellular amount of 8-PN decreased slowly, and that of 6-PN decreased dramatically (Figure 5 and Figure 6). These results indicate that the intracellular residence time of prenylated flavonoids increased and affected the efflux time. Mukai et al. reported the intracellular localization of flavonols and flavones in fluorescence microscopy experiments; their behaviors differed depending on the substituent [43]. To clarify the anti-influenza virus mechanism of each flavonoid, it is necessary to investigate the intracellular uptake mechanisms, such as endocytosis, passive diffusion, and membrane transporters, in detail. The kinetics of this prenylated compound are of great interest and provide pharmacological insights into antiviral strategies.

This result suggests different intracellular kinetics, which may also be involved in the antiviral effects. Although 8-PN and 6-PN commonly block the late stage of viral growth (6–8 h), a time-of-addition assay revealed that the inhibitory effect on the antiviral effect differs depending on the position of the substituent (Figure 7A). Interestingly, 6-PN showed a different inhibitory effect than 8-PN 4–6 h after infection. In the influenza A virus, it has been reported that assembly of viral proteins and budding of mature virus particles take place in cells approximately 6–8 h after infection [44]. This suggests the possibility of inhibiting the growth process until viral maturation. DMEM containing bovine serum albumin (BSA) was used for the time-of-addition assay. From the results shown in Figure 5 and Figure 6, it is likely that part of the prenylated naringenin in the cells was bound to albumin in the medium. This binding may be involved in the intracellular and extracellular kinetics. Interactions between in vivo substances and flavonoids may affect the kinetics and anti-influenza virus efficacy. This experiment, in addition to Hanada’s report [25], indicates that prenylation of naringenin confers broad antiviral effects on influenza A and B viruses, broadening its potential as a novel antiviral agent. Moreover, in the process of chemical synthesis, diastereomeric mixtures of naringenin derivatives were generated and their separation was difficult due to their physical properties. Future research will focus on the effects of structure mixtures and subtle structural differences. However, this work is one of the few reports investigating the biological properties of flavonoid structures against influenza virus.

We were able to elucidate some of the structure-activity relationships of flavanone derivatives against influenza viruses, providing important information for antiviral strategies. Further study of this property may result in qualitative improvements in the pharmacological activity of drugs against influenza viruses.

## 4. Materials and Methods

### 4.1. General

All materials used for chemical synthesis not explicitly mentioned were purchased from Fujifilm Wako Pure Chemical Co. (Osaka, Japan), Tokyo Kasei Kogyo Co., Ltd. (Tokyo, Japan), Nacalai Tesque, Inc. (Kyoto, Japan), and Sigma-Aldrich (St. Louis, MO, USA). (±)—Naringenin was purchased from Cayman Chemical Co., (Ann Arbor, MI, USA) and was used directly without purification. ^1^H NMR (500 MHz) and ^13^C NMR (125 MHz) spectra were recorded using a JEOL JNM-ECP500 spectrometer. Chemical shift values are expressed in ppm relative to the solvent residual signals of CDCl_3_ (7.26 ppm), DMSO-*d6* (2.50 ppm) in ^1^H NMR, and CDCl_3_ (77.1 ppm), DMSO-*d6* (39.5 ppm) in ^13^C NMR. TMS (tetramethylsilane) was used as an internal standard in case of using CDCl_3_. Abbreviations: s, singlet; d, doublet; t, triplet; m, multiplet; br, broad. The coupling constant values are expressed in hertz. High resolution mass spectrometry (HR-MS) was performed using an LTQ-Orbitrap XL Mass Spectrometer (Thermo Fisher Scientific Inc., Waltham, MA, USA) or LCMS-9030 mass spectrometer (Shimadzu, Kyoto, Japan). Melting points were measured using a Yanaco micro melting point apparatus, without correction. Flash column chromatography was performed using silica gel (Wakosil C-200; Fujifilm Wako Pure Chemical Co., Osaka, Japan). Analytical thin-layer chromatography was performed on Silica gel 60 F_254_ glass plates (Merck Millipore, Burlington, MA, USA) and visualized using a UV lamp. All compounds were dissolved in dimethyl sulfoxide and stored at −30 °C. The compounds were synthesized according to the literature [45,46,47,48,49,50] and supplemental materials.

### 4.2. Cell and Viruses

MDCK cells were grown in Eagle’s minimum essential medium (MEM; Fujifilm Wako Pure Chemical Co., Osaka, Japan) containing 7% FBS. Type A influenza virus H1N1 (PR/8/34) was used in the experiments. To infect the cells, the virus was diluted in serum-free MEM containing 0.04% BSA (fraction V; Sigma-Aldrich, St. Louis, MO, USA) and incubated with the cells at a multiplicity of infection of 0.001 for 1 h at 37 °C. The medium was then removed and replaced with serum-free DMEM containing 0.4% BSA and 2 µg/mL acetyl trypsin (Sigma-Aldrich, St. Louis, MO, USA) for the remainder of the infection period. For fluorescence microscopy, cells were grown in DMEM (Fujifilm Wako Pure Chemical Co., Osaka, Japan) without phenol red containing 1% FBS and 2 mM glutamine.

MDCK cells and the A/PR/8/34 strain were obtained from the Research Institute for Microbial Diseases (Osaka University, Suita, Osaka, Japan).

### 4.3. Cell Viability Determination

Cell viability was determined using the Cell Proliferation Kit I (MTT) (F. Hoffmann–La Roche Ltd., Basel, Switzerland). The cytopathic effect on the virus-infected cells treated with various concentrations of flavonoids was observed under a microscope. The experiment was performed at a concentration that was not cytotoxic.

### 4.4. Determination of Cell Viability in the Presence of Naringenin Derivatives

The effects of naringenin derivatives on viral yield were determined according to a previously described procedure [51,52]. The focus-forming reduction assay for viral activity was performed according to a previously described procedure [51,52].

### 4.5. Cellular Uptake of Naringenin and Its Derivatives

Fluorescence microscopy experiments were performed according to Wolff et al. [30]. Briefly, cells were plated in glass-bottom dishes (Matsunami, Osaka, Japan) and cultured in a medium without phenol red. Cells were exposed to 30 μM naringenin and its derivatives, and phase-contrast and fluorescence images were acquired for a fixed exposure time before and after the addition of the compounds at defined intervals. For the imaging, we prepared a custom-built filter (ex 470/40, em 605/70). Upon excitation at approximately 470 nm (bandwidth of 40 nm), a major emission was observed at approximately 605 nm (bandwidth of 70 nm). The emission signal from naringenin and its derivatives was detectable at wavelengths > 565 nm, making it possible to design filters that avoid cellular autofluorescence in the green range.

Fluorescence microscopy of living and fixed cells was performed using an inverted fluorescence phase-contrast microscope (Keyence BZ-X800), consisting of an infinity optical system (Nikon CFI60 series with software BZ-H4 series; Keyence, Osaka, Japan).

### 4.6. Estimation of Intracellular Naringenin and Prenylated Naringenin

Cells were cultured on glass plates and incubated in a medium supplemented with the compound for 180 min. They were then washed and fixed with 4% paraformaldehyde for 30 min. After washing with PBS, DAPI staining was performed. Intracellular distribution was photographed and observed using an inverted fluorescence phase-contrast microscope (BZ-X800, Keyence, Osaka, Japan).

### 4.7. Time-of-Addition Assay

We conducted a time-of-addition assay as previously described [51,52]. DMEM containing prenylated naringenin (30 μM) was added at −1–0 h (adsorption), 0–8 h (replication), 0–4 h, 0–2 h, 2–4 h, 4–8 h, 4–6 h, and 6–8 h. Furuta et al. [44] estimated the intracellular cycle of infected cells before extracellular budding to be 8 h, and growth stages were assumed to occur at 2 h intervals.

### 4.8. Statistical Analysis

Statistical analyses were performed using the unpaired t-test and analysis of variance with the Tukey–Kramer test using SPSS ver. 21.0 software (SPSS, Inc., Chicago, IL, USA). Statistical significance was set at *p* < 0.05.

## 5. Conclusions

Naringenin, a flavanone found mainly in vegetables and fruits, has been reported to possess various physiological functions, including antiviral activity. In this study, we investigated the relationship between the antiviral activity of naringenin, prenylated naringenin, and their derivatives against influenza infection as well as the cellular uptake of these flavanones. Our results indicated that the steric effect of the compounds is an important factor in their antiviral activity. In the structure of naringenin, the substituent that enhances antiviral activity is the prenyl group, and its structure and position have a significant effect on its cell permeability. We hope that the results of this study will be useful for the development of future therapeutic drugs against viruses.

## Figures and Tables

**Table 1:** Anti-viral effects of prenylated naringenin and derivatives.

Compound Name	Structure	IC_50_ ^a^ (μM)	SI ^b^
(±)-Naringenin (Na)	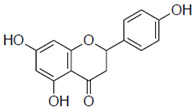	290 ± 16.2	4.9
Naringenin-4′,7′-diacetate (Na-di)	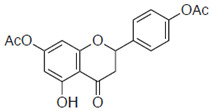	ND	ND
8-prenylnaringenin (8-PN)	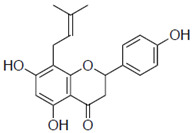	24 ± 0.6	4.9
6-prenylnaringenin (6-PN)	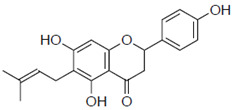	38 ± 4.7	5.5
**1a**	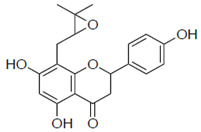	59 ± 9.0	2.7
**1b**	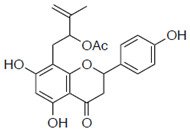	ND	ND
**1e**	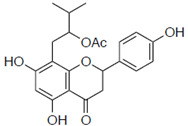	ND	ND
**1c**	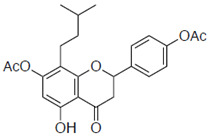	42 ± 2.1	6.3
**1d**	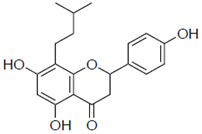	61 ± 14.0	3.0
**2a**	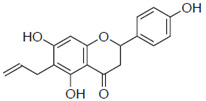	103 ± 3.8	0.8
**2b**	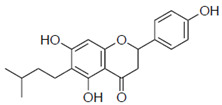	58 ± 15.2	1.3
**2c**	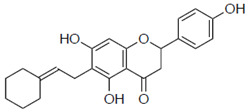	ND	ND
**2d**	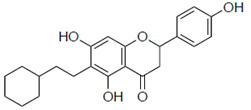	ND	ND

^a^ Values are the average of the results obtained using various final concentration of compounds and MDCK cells from three independent experiments. The IC_50_ of compounds were reported as μM. The values after ‘‘±’’ in the IC50 are the standard deviations. The IC50 value for oseltamivir acid in the A/PR/8/34 strain was 2.0 ± 0.7 nM. ^b^ Selectivity index = CC_50_/IC_50_(CC_50_, 50% cytotoxicity concentration; IC_50_, 50% maximal inhibitory concentration); ND: Not detected.

## Data Availability

Data is contained within the article.

## Supplementary Materials

The following supporting information can be downloaded at: https://www.mdpi.com/article/10.3390/ph15121480/s1. Figure S1. ^1^H NMR chart of compound **3**; Figure S2. ^1^H NMR chart of compound **4**; Figure S3. ^1^H NMR chart of compound **5**; Figure S4. ^1^H NMR chart of compound **7**; Figure S5. ^1^H NMR chart of compound **8**-PN; Figure S6. ^1^H NMR chart of compound **1c**; Figure S7. ^13^C NMR chart of compound **1c**; Figure S8. ESI-HR-MS chart of compound **1c**; Figure S9. ^1^H NMR chart of compound **1d**; Figure S10. ^13^C NMR chart of compound **1d**; Figure S11. ESI-HR-MS chart of compound **1d**; Figure S12. ^1^H NMR chart of compound **8**; Figure S13. ^13^C NMR chart of compound **8**; Figure S14. ESI-HR-MS chart of compound **8**; Figure S15. ^1^H NMR chart of compound **1a**; Figure S16. ^13^C NMR chart of compound **1a**; Figure S17. ESI-HR-MS chart of compound **1a**; Figure S18. ^1^H NMR chart of compound **1b**; Figure S19. ^13^C NMR chart of compound **1b**; Figure S20. ESI-HR-MS chart of compound **1b**; Figure S21. ^1^H NMR chart of compound **10**; Figure S22. ^13^C NMR chart of compound **10**; Figure S23. ESI-HR-MS chart of compound **10**; Figure S24. ^1^H NMR chart of compound **1e**; Figure S25. ^13^C NMR chart of compound **1e**; Figure S26. ESI-HR-MS chart of compound **10**; Figure S27. ^1^H NMR chart of compound **2a**; Figure S28. ^13^C NMR chart of compound **2a**; Figure S29. ^1^H NMR chart of compound **6**-PN; Figure S30. ^1^H NMR chart of compound **12**; Figure S31. ^1^H NMR chart of compound **2b**; Figure S32. ^1^H NMR chart of compound **13**; Figure S33. ^13^C NMR chart of compound **13**; Figure S34. ESI-HR-MS chart of compound **13**; Figure S35. ^1^H NMR chart of compound **2c**; Figure S36. ^13^C NMR chart of compound **2c**; Figure S37. ESI-HR-MS chart of compound **2c**; Figure S38. ^1^H NMR chart of compound **14**; Figure S39. ^13^C NMR chart of compound **14**; Figure S40. HR-MS chart of compound **14**; Figure S41. ^1^H NMR chart of compound **2d**; Figure S42. ^13^C NMR chart of compound **2d**; Figure S43. ESI-HR-MS chart of compound **2d**. Refs. [53,54] are cited in supplementary materials
