# Supplementary material for: Effect of Structural Differences in Naringenin, Prenylated Naringenin, and Their Derivatives on the Anti-Influenza Virus Activity and Cellular Uptake of Their Flavanones
Source: Pharmaceuticals (Basel). 2022 Nov 28;15(12):1480. doi: 10.3390/ph15121480 (PMC9785311; doi:10.3390/ph15121480)
Supplement: Supplementary file 1 [file pharmaceuticals-15-01480-s001.zip › pharmaceuticals-1982419-supplementary.pdf]

Supplementary data

materials and methods

All materials used for chemical synthesis not explicitly mentioned were purchased from Fujifilm Wako Pure Chemical Co. (Osaka, Japan), Tokyo Kasei Kogyo Co., Ltd. (Tokyo, Japan), Nacalai Tesque, Inc. (Kyoto, Japan), and Sigma-Aldrich (St. Louis, MO, USA). (±) - Naringenin was purchased from Cayman Chemical Co., (Ann Arbor, MI, USA) and was used directly without purification. ^{1}H NMR (500 MHz) and ^{13}C NMR (125 MHz) spectra were recorded using a JEOL JNM-ECP500 spectrometer. Chemical shift values are expressed in ppm relative to the solvent residual signals of $CDCl_3$ (7.26 ppm), DMSO-*d6* (2.50 ppm) in ^{1}H NMR, and $CDCl_3$ (77.1 ppm), DMSO-*d6* (39.5 ppm) in ^{13}C NMR. TMS (tetramethylsilane) was used as an internal standard in case of using $CDCl_3$. Abbreviations: s, singlet; d, doublet; t, triplet; m, multiplet; br, broad. The coupling constant (*J*) values are expressed in hertz. High resolution mass spectrometry (HR-MS) was performed using an LTQ-Orbitrap XL Mass Spectrometer (Thermo Fisher Scientific Inc., Waltham, MA, USA) or LCMS-9030 mass spectrometer (Shimadzu, Kyoto, Japan). Melting points were measured using a Yanaco micro melting point apparatus, without correction. Flash column chromatography was performed using silica gel (Wakosil C-200; Fujifilm Wako Pure Chemical Products Co., Osaka, Japan). Analytical thin-layer chromatography was performed on Silica gel 60 F_{254} glass plates (Merck Millipore, Burlington, MA, USA) and visualized using a UV lamp. All compounds were dissolved in dimethyl sulfoxide and stored at -30 °C.

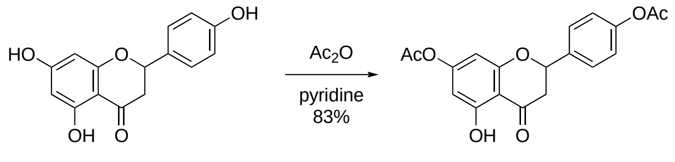

1) Naringenin-4′,7-diacetate (3) [1]

According to the reported procedure [1], to a solution of (±) - naringenin (5.44 g, 20.0 mmol) in 20.0 mL of anhydrous pyridine was added dropwise acetic anhydride (4.08 g, 40.0 mmol) at rt, and then stirred for overnight. The resulting suspension was poured into ice water, and the precipitate was collected by filtration, washed twice with water and recrystallized from methanol to give 5.92 g (83%) of titled compound as colorless needles. ^{1}H NMR (DMSO-*d6*, 500 MHz) δ 2.26 (s, 3H), 2.28 (s, 3H), 2.90 (dd, 1H, *J* = 2.8, 17.2), 3.46 (dd, 1H, *J* = 13.3, 17.2), 5.73 (dd, 1H, *J* = 2.8, 13.3), 6.37 (d, 1H, *J* = 2.3), 6.39 (d, 1H, *J* = 2.3), 7.21 (d, 2H, *J* = 8.3), 7.59 (d, 2H, *J* = 8.3), 11.93 (s, 1H).

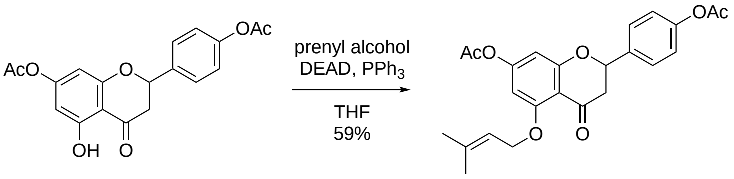

3 4

2-(4-Acetoxyphenyl)-5-(3-methylbut-2-enyloxy)-4-oxochroman-7-yl acetate (4) [1]

According to the reported procedure [1], to a solution of **3** (3.56 g, 10 mmol) and triphenylphosphine (3.20 g, 12.2 mmol) in 120 mL of anhydrous THF (with BHT as a stabilizer) was added prenyl alcohol (1.29 g, 15.0 mmol) under an argon atmosphere. A solution of diethyl azodicarboxylate (2.78 g, 16.0 mmol) in 37.5 mL of anhydrous THF (with BHT as a stabilizer) was slowly added to the mixture over 2 h using syringe pump under ice water bath cooling, and then stirred for 2 h at rt. After removal of the solvent by evaporation, the crude product was purified by column chromatography eluting with ethyl acetate and hexane in 1/3 to 1/2 to give 3.50 g of white solid. The solid was recrystallized from ethyl acetate and hexane gave 2.51 g of titled compound as a white powder. ^{1}H NMR ($CDCl_3$, 500 MHz) δ 1.74 (s, 3H), 1.79 (s, 3H), 2.30 (s, 3H), 2.32 (s, 3H), 2.82 (dd, 1H, *J* = 3.2, 16.5), 3.02 (dd, 1H, *J* = 13.2, 16.5), 4.61 (br-s, 2H), 5.43 (dd, 1H, *J* = 3.2, 13.2), 5.52–5.55 (m, 1H), 6.32 (d, 1H, *J* = 2.3), 6.43 (d, 1H, *J* = 2.3), 7.15 (d, 2H, *J* = 8.7), 7.47 (d, 2H, *J* = 8.7).

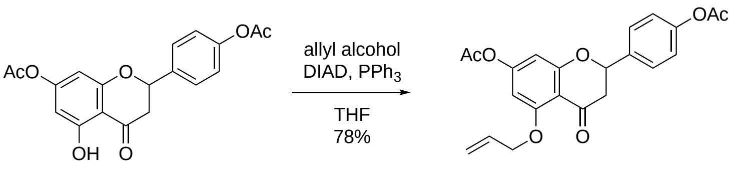

3 5

Acetic acid 2-(4-acetoxyphenyl)-5-allyloxy-4-oxochroman-7-yl ester (5) [2]

According to the similar literature procedure [2], to a solution of **3** (3.56 g, 10 mmol) and triphenylphosphine (4.45 g, 17 mmol) in 100 mL of anhydrous THF (with BHT as a stabilizer) was added allyl alcohol (0.87 g, 15.0 mmol) under an argon atmosphere. A solution of diisopropyl azodicarboxylate (3.23 g, 16.0 mmol) in 50 mL of anhydrous THF (with BHT as a stabilizer) was slowly added to the mixture over 5 h using syringe pump under ice water bath cooling, and then stirred for overnight at rt.

After removal of the solvent by evaporation, the crude product was purified by column chromatography eluting with ethyl acetate and hexane in 1/4 ratio give 3.09 g of a pale yellow solid. The solid was recrystallized from ethyl acetate and hexane gave 2.02 g (51%) of titled compound as a white powder ^1H NMR ($CDCl_3$, 400 MHz) δ 2.30 (s, 3H), 2.32 (s, 3H), 2.83 (dd, 1H, *J* = 3.2, 16.4), 3.03 (dd, 1H, *J* = 13.2, 16.4), 4.62 (d, 2H, *J* = 4.4), 5.35 (dd, 1H, *J* = 1.6, 10.8), 5.44 (dd, 1H, *J* = 3.2, 16.4), 5.64 (dd, 1H, *J* = 1.6, 17.6), 6.02–6.12 (m, 1H), 6.32 (d, 1H, *J* = 2.0), 6.45 (d, 1H, *J* = 2.0), 7.15 (d, 2H, *J* = 8.8), 7.47 (d, 2H, *J* = 8.8).

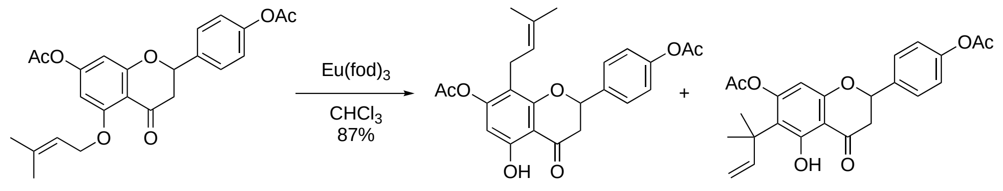

2-(4-Acetoxyphenyl)-5-hydroxy-8-(3-methylbut-2-enyl)-4-oxochroman-7-yl acetate (6) [1]
2-(4-Acetoxyphenyl)-6-(1,1-dimethylallyl)-5-hydroxy-4-oxochroman-7-yl acetate (15) [1]

According to the reported procedure [1], a solution of **4** (3.50 g, 8.25 mmol) and $Eu(fod)_3$ (868 mg, 0.837 mmol) in 1.40 mL of dry $CHCl_3$ was stirred at 60℃ for 12 h under an argon atmosphere. After complete consumption of the starting material which was checked by TLC, the reaction mixture was concentrated in vacuo, and the residue was purified by column chromatography eluting with ethyl acetate and hexane in 1/3 ratio to give 3.34 g of **6** which include a small amount of **15** as a pale yellow solid. The compound **6** was used directly for the next step without further purification.

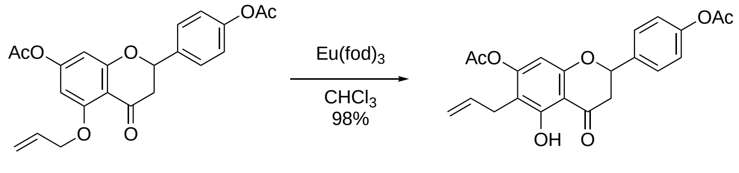

Acetic acid 2-(4-acetoxyphenyl)-6-allyl-5-hydroxy-4-oxochroman-7-yl ester (7) [2]

According to the similar literature procedure [2], a solution of **5** (1.19 g, 3 mmol) and $Eu(fod)_3$ (311 mg, 0.3 mmol) in 1.40 mL of dry $CHCl_3$ was stirred at 85℃ under an argon atmosphere. After refluxing in a sealed tube for 2 days, the reaction mixture was concentrated in vacuo, and the residue was purified by column chromatography eluting with ethyl acetate and hexane in 1/5 ratio to give 1.17 g (98%) of titled

compound as a white powder. ^{1}H NMR ($CDCl_3$, 500 MHz) δ 2.30 (s, 3H), 2.32 (s, 3H), 2.88 (dd, 1H, *J* = 3.5, 17.5), 3.11 (dd, 1H, *J* = 13.5, 17.5), 3.28 (d, 2H, *J* = 6.5), 4.99–5.04 (m, 2H), 5.45 (dd, 1H, *J* = 3.5, 13.5), 5.82–5.90 (m, 1H), 6.31 (s, 1H), 7.16 (d, 2H, *J* = 9.0), 7.47 (d, 2H, *J* = 9.0), 12.18 (s, 1H).

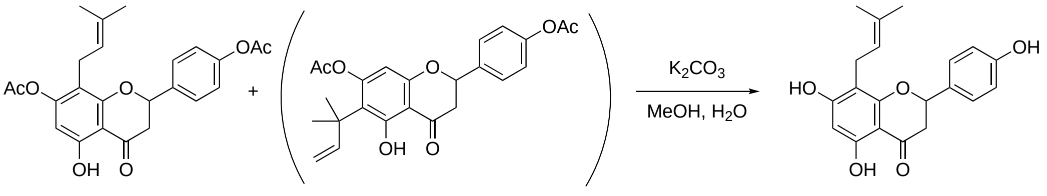

8-Prenylnaringenin (8-PN) [1]

According to the reported procedure [1], to a solution of a mixture of **6** and **15** (3.34 g, 7.88 mmol) in 82.0 mL of methanol was added K_2CO_3 (163 mg, 1.18 mmol) and 0.20 mL of water. The mixture was heated at 40°C for 4 hrs. After complete consumption of the starting material which was checked by TLC, the reaction mixture was concentrated in vacuo. 10.0 mL of satd. aqueous $NaHCO_3$ was added to the residue, and the mixture was extracted twice with CH_2Cl_2. The combined organic layer was dried over Na_2SO_4, filtered, and concentrated in vacuo. The crude products were purified by column chromatography eluting with methanol and CH_2Cl_2 in 1/20 ratio to give pale yellow solid, and the separated component was recrystallized from $CHCl_3$ to give 1.20 g (48%, 2 steps from 4) of titled compound 8-PN as a pale yellow solid. In order to clarify and prove the structural comparison of 8-PN and 6-PN, ^{1}H NMR was measured using two deuterated solvents (DMSO-*d6* and $CDCl_3$). The ^{1}H NMR spectrum of 8-PN using DMSO-*d6* was completely agreement with the values reported in the reference [1].^{1}H NMR (DMSO-*d6*, 500 MHz) δ 1.54 (s, 3H), 1.59 (s, 3H), 2.72 (dd, 1H, *J* = 3.2, 17.0), 3.08 (d, 2H, *J* = 7.3), 3.21 (dd, 1H, *J* = 12.8, 17.0), 5.09 (t, 1H, *J* = 7.3), 5.43 (dd, 1H, *J* = 3.2, 12.8), 5.97 (s, 1H), 6.80 (d, 2H, *J* = 8.3), 7.32 (d, 2H, *J* = 8.3), 9.58 (s, 1H), 10.76 (br-s, 1H), 12.12 (s, 1H). ^{1}H NMR ($CDCl_3$, 500 MHz) δ 1.73 (s, 6H), 2.80 (dd, 1H, *J* = 3.0, 17.0), 3.05 (dd, 1H, *J* = 13.0, 17.0), 3.31 (d, 2H, *J* = 7.0), 4.92 (s, 1H), 5.21 (t, 1H, *J* = 7.0), 5.35 (dd, 1H, *J* = 3.0, 13.0), 6.02 (s, 1H), 6.05 (s, 1H), 6.88 (d, 2H, *J* = 8.5), 7.33 (d, 2H, *J* = 8.5), 12.00 (s, 1H).

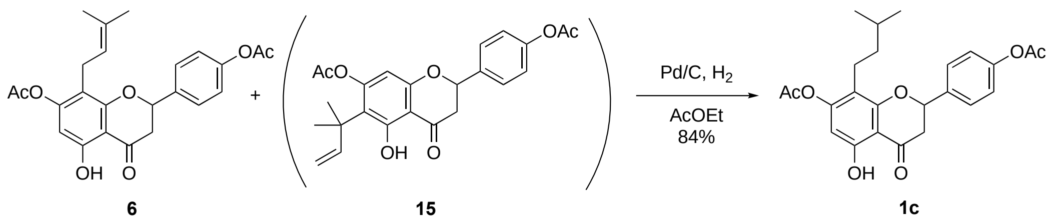

Acetic acid 2-(4-acetoxyphenyl)-5-hydroxy-8-(3-methylbutyl)-4-oxochroman-7-yl ester (1c)

To a solution of **6** and **15** (212 mg, 0.5 mmol) in 4 mL of ethyl acetate was added wet 10 w/w% Pd/C (42 mg) under an argon atmosphere. The argon gas filled in the flask was exchanged to hydrogen gas, and the reaction mixture was stirred vigorously at rt for overnight. After complete consumption of the starting material which was checked by TLC, the catalyst was removed by filtration using short plug of Celite® pad, and the filtrate was concentrated in vacuo. The crude product was purified by column chromatography eluting with ethyl acetate and hexane in 1/4 ratio to give white solid. The solid was recrystallized from ethyl acetate and hexane to give 178 mg (84%) of titled compound as colorless needles of mp 113.5–114.5 °C. ^{1}H NMR ($CDCl_3$, 500 MHz) δ 0.88 (d, *J* = 6.0, 6H), 1.28–1.39 (m, 2H), 1.50–1.56 (m, 1H), 2.32 (s, 3H), 2.33 (s, 3H), 2.40–2.49 (m, 2H), 2.90 (dd, 1H, *J* = 2.8, 17.0), 3.06 (dd, 1H, *J* = 13.3, 17.0), 5.46 (dd, 1H, *J* = 2.8, 13.3), 6.31 (s, 1H), 7.20 (d, 2H, *J* = 8.7), 7.47 (d, 2H, *J* = 8.7), 11.71 (s, 1H). ^{13}C NMR ($CDCl_3$, 125 MHz) δ 20.9, 21.1, 21.4, 22.4, 22.5, 28.0, 38.4, 43.7, 78.4, 104.0, 106.6, 114.9, 122.1, 127.0, 136.0, 150.8, 156.8, 159.9, 160.6, 168.5, 169.4, 197.2. ESI-HR-MS $[M+Na]^+$ m/z: Calcd for $C_{24}H_{26}O_7Na$ 449.1576; Found 449.1569.

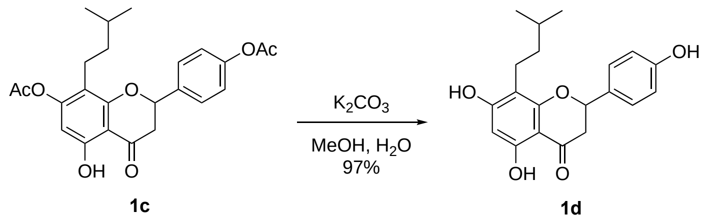

5,7-Dihydroxy-2-(4-hydroxyphenyl)-8-(3-methylbutyl)chroman-4-one (1d)

A same procedure for the preparation of **8-PN** using **1c** (213 mg, 0.5 mmol) as a substrate was conducted to give 165 mg (97%) of titled compound as a white powder of mp 199.0–200.5 °C. ^{1}H NMR (DMSO-*d6*, 500 MHz) δ 0.82 (d, *J* = 6.5, 6H), 1.24–1.28 (m, 2H), 1.40–1.48 (m, 1H), 2.40 (t, 2H, *J* = 7.3), 2.72 (dd, 1H, *J*

= 2.8, 17.0), 3.15 (dd, 1H, *J* = 12.8, 17.0), 5.40 (dd, 1H, *J* = 2.8, 12.8), 5.96 (s, 1H), 6.78 (d, 2H, *J* = 8.7), 7.30 (d, 2H, *J* = 8.7), 9.55 (br-s, 1H), 10.68 (br-s, 1H), 12.09 (s, 1H). ^{13}C NMR (DMSO-*d6,* 125 MHz) δ 19.9, 22.4, 22.6, 27.4, 38.1, 41.9, 78.0, 95.2, 101.8, 108.0, 115.1, 127.8, 129.3, 157.5, 159.8, 161.0, 164.5, 196.7. ESI-HR-MS [M+H]$^+$ m/z: Calcd for $C_{20}H_{23}O_5$ 343.1545; Found 343.1540.

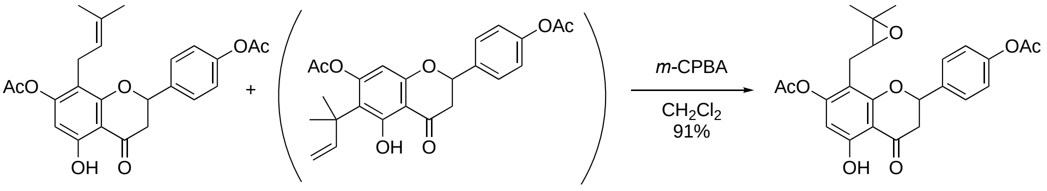

6 **15** **8**

Acetic acid 2-(4-acetoxyphenyl)-8-(3,3-dimethyloxiranylmethyl)-5-hydroxy-4-oxochroman-7-yl ester (8)

To a solution of a mixture of **6** and **15** (424 mg, 1.00 mmol) in 3.00 mL of CH_2Cl_2 was added wet *m*-chloroperbenzoic acid (70% purity, 368 mg, 1.5 mmol) under ice water bath cooling. The mixture was warm up to rt and stirred for 2 hrs. After complete consumption of the starting material which was checked by TLC, the reaction mixture was diluted with $CHCl_3$ and water. The whole solution was successively washed with 1w/w% aqueous $Na_2S_2O_3$, and brine. The combined organic layer was dried over Na_2SO_4, filtered, and concentrated. The crude product was purified by column chromatography eluting with ethyl acetate and hexane in 1/5 to 1/3 ratio to give white solid. The solid was recrystallized from ethyl acetate and hexane to give 400 mg (91%) of titled compound (diastereomeric mixture) as a white powder of mp 110.0–112.0 °C. ^{1}H NMR ($CDCl_3$, 500 MHz) δ 1.23–1.26 (m, 6H), 2.33 (s, 6H), 2.63–2.70 (m, 1H), 2.75–2.84 (m, 2H), 2.88 (dd, 0.5H, *J* = 3.2, 5.5), 2.91 (dd, 0.5H, *J* = 3.2, 5.5), 3.07–3.17 (m, 1H), 5.46 (t, 0.5H, *J* = 3.2), 5.48 (t, 0.5H, *J* = 3.2), 6.36 (s, 0.5H), 6.37 (s, 0.5H), 7.17–7.19 (m, 2H), 7.47–7.49 (m, 2H), 11.78 (s, 0.5H), 11.80 (s, 0.5H). ^{13}C NMR ($CDCl_3$, 125 MHz) δ 18.9, 21.10, 21.14, 23.4, 24.7, 43.3, 43.8, 58.76, 58.82, 63.1, 78.95, 79.00, 104.4, 104.5, 106.46, 106.51, 110.1, 110.2, 122.16, 122.19, 127.2, 127.5, 135.5, 135.7, 151.0, 151.1, 157.15, 157.22, 160.16, 160.21, 161.45, 161.48, 168.33, 168.34, 169.35, 169.37, 197.0, 197.1 (six alkyl carbons were duplicated). ESI-HR-MS [M+Na]$^+$ m/z: Calcd for $C_{24}H_{24}O_8Na$ 463.1369; Found

463.1359.

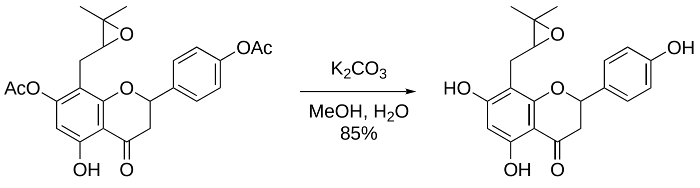

8 1a

8-(3,3-Dimethyloxiranylmethyl)-5,7-dihydroxy-2-(4-hydroxyphenyl)chroman-4-one (1a)

A same procedure for the preparation of **8-PN** using **8** (110 mg, 0.25 mmol) as a substrate was conducted to give 76 mg (85%) of titled compound (diastereomeric mixture) as a white powder of mp 191.5–193.0 °C. ^{1}H NMR (DMSO-*d6,* 500 MHz) δ 1.08 (s, 3H), 1.11 (s, 3H), 2.71 (dd, 1H, *J* = 2.8, 17.4), 2.91 (dd, 2H, *J* = 2.8, 8.7), 4.64–4.68 (m, 2H), 5.47–5.51 (m, 1H), 5.96 (s, 1H), 6.79 (d, 2H, *J* = 7.8), 7.32 (d, 2H, *J* = 7.8), 9.60 (s, 1H), 12.427 (s, 0.5H), 12.434 (s, 0.5H). ^{13}C NMR (DMSO-*d6,* 125 MHz) δ 24.9, 25.0, 25.59, 25.64, 25.98, 26.02, 39.8, 41.9, 42.0, 69.9, 70.0, 78.4, 78.5, 90.5, 91.5, 102.2, 102.3, 104.67, 104.73, 115.2, 128.3, 128.8, 156.8, 156.9, 157.8, 164.0, 168.55, 168.58, 196.2 (one alkyl carbon and six aryl carbons were duplicated). ESI-HR-MS [M+Na]$^{+}$ m/z: Calcd for $C_{20}H_{20}O_6Na$ 379.1158; Found 379.1153.

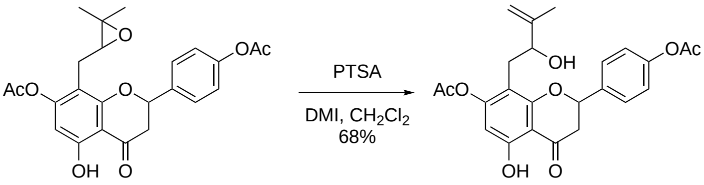

8 9

Acetic acid 2-(4-acetoxyphenyl)-5-hydroxy-8-(2-hydroxy-3-methylbut-3-enyl)-4-oxochroman-7-yl ester (9)

According to the reported procedure [3], to a solution of **8** (589 mg, 1.34 mmol) in 7 mL of CH_2Cl_2 was added 1,3-dimethylimidazolidin-2-one (306 mg, 2.68 mmol) and p-toluenesulfonic acid monohydrate (127 mg, 0.670 mmol), and the solution was stirred at rt for overnight. The reaction was quenched with water, and the reaction mixture was extracted twice with CH_2Cl_2. The combined organic extract was successively washed with water and brine, dried over Na_2SO_4, filtered and concentrated in vacuo. The residue was purified by column chromatography eluting with ethyl acetate and hexane in 1/5 to 1/3

ratio to give 401 mg (68%) of titled compound as white solid. This compound was used for the next step without further purification.

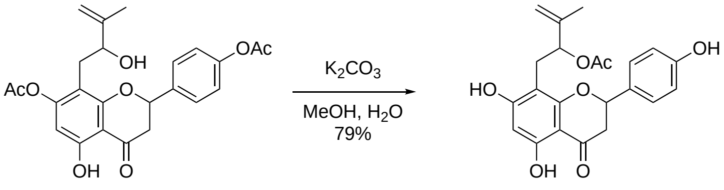

9 → 1b

Acetic acid 1-[5,7-dihydroxy-2-(4-hydroxyphenyl)-4-oxochroman-8-ylmethyl]-2-methylallyl ester (1b)

A same procedure for the preparation of **8-PN** using **9** (138 mg, 0.314 mmol) as a substrate was conducted to give 99 mg (79%) of titled compound (diastereomeric mixture) as a white powder of mp 189.0–191.5 °C. ^{1}H NMR (DMSO-*d6*, 500 MHz) δ 1.57, 1.62 (s, 3H), 1.90, 1.91 (s, 3H), 2.67–2.75 (m, 3H), 3.12–3.19 (m, 1H), 4.65–4.76 (m, 2H), 5.28–5.40 (m, 2H), 5.95 (s, 1H), 6.79 (d, 2H, *J* = 8.3), 7.32–7.35 (m, 2H), 9.57 (br-s, 1H), 10.88 (br-s, 1H), 12.13 (s, 0.5H), 12.14 (s, 0.5H). ^{13}C NMR (DMSO-*d6*, 125 MHz) δ 17.7, 18.0, 20.8, 26.10, 26.13, 41.9, 42.3, 75.47, 75.52, 78.3, 78.5, 95.2, 101.61, 101.64, 103.0, 103.1, 111.8, 112.1, 115.1, 127.86, 127.91, 129.1, 129.2, 143.0, 143.3, 157.54, 157.55, 160.6, 160.7, 161.6, 164.92, 164.95, 169.4, 196.69, 196.71 (one alkyl carbon and four aryl carbons were duplicated). ESI-HR-MS $[M+Na]^+$ m/z: Calcd for $C_{22}H_{22}O_7Na$ 421.1263; Found 421.1257.

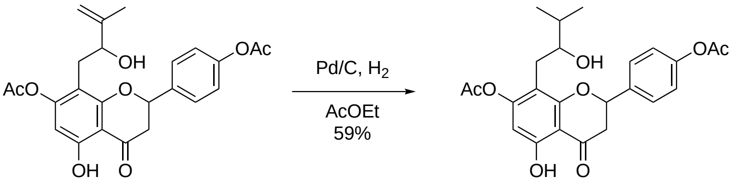

9 → 10

Acetic acid 2-(4-acetoxyphenyl)-5-hydroxy-8-(2-hydroxy-3-methylbutyl)-4-oxochroman-7-yl ester (10)

A same procedure for the preparation of **1c** using **9** (367 mg, 0.830 mmol) as a substrate was conducted to give 218 mg (59%) of titled compound (diastereomeric mixture) as white solid of mp 182.5–184.0 °C. ^{1}H NMR ($CDCl_3$, 500 MHz) δ 0.82–0.95 (m, 6H), 1.75, 1.89 (br-s, 1H), 2.08, 2.10 (s, 3H), 2.33 (s, 3H), 2.47–2.53 (m, 1H), 2.80 (t, 1H, *J* = 15.6), 3.02 (t, 1H, *J* = 15.6), 3.12 (dd, 1H, *J* = 8.7, 14.2), 4.46–4.52 (m, 1H), 5.38 (t, 1H, *J* = 11.5), 6.12, 6.14 (s, 1H), 7.17 (d, 2H, *J* = 8.3), 7.47 (br-s, 2H), 8.23, 8.44 (br-s, 1H), 11.98 (s, 0.5H),

12.00 (s, 0.5H). ^{13}C NMR ($CDCl_3$, 125 MHz) δ 15.95, 16.02, 19.4, 19.6, 21.15, 21.18, 24.7, 24.9, 29.9, 30.1, 43.3, 43.6, 78.7, 79.0, 79.4, 80.0, 97.6, 97.7, 102.7, 102.8, 102.87, 102.88, 122.0, 127.15, 127.19, 136.0, 136.2, 150.86, 150.89, 160.0, 160.1, 162.9, 163.0, 164.7, 165.0, 169.36, 169.39, 173.3, 173.5, 195.7 (one carbonyl carbon and one aryl carbon were duplicated). ESI-HR-MS $[M+Na]^+$ m/z: Calcd for $C_{24}H_{26}O_8Na$ 465.1525; Found 465.1519.

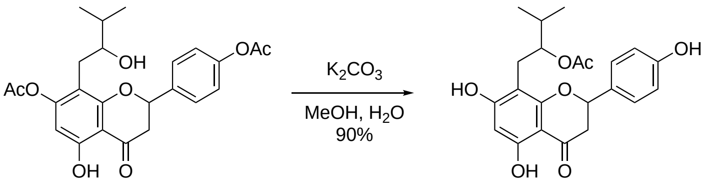

10 **1e**

Acetic acid 1-[5,7-dihydroxy-2-(4-hydroxyphenyl)-4-oxochroman-8-ylmethyl]-2-methylpropyl ester (1e)

A same procedure for the preparation of **8-PN** using **10** (218 mg, 0.493 mmol) as a substrate was conducted to give 178 mg (90%) of titled compound (diastereomeric mixture) as a pale yellow solid of mp 155.0–156.0 °C. ^1H NMR (DMSO-*d6*, 500 MHz) δ 0.79–0.83 (m, 6H), 1.67–1.72 (m, 1H), 1.84 (s, 3H), 2.59–2.74 (m, 3H), 3.10–3.17 (m, 1H), 4.85–4.94 (m, 1H), 5.36–5.40 (m, 1H), 5.94 (s, 1H), 6.79 (d, 2H, *J* = 8.3), 7.32–7.35 (m, 2H), 9.56 (br-s, 1H), 10.81 (br-s, 1H), 12.12 (s, 0.5H), 12.14 (s, 0.5H). ^{13}C NMR (DMSO-*d6*, 125 MHz) δ 17.3, 17.4, 18.8, 18.9, 20.7, 24.4, 24.6, 31.0, 31.1, 41.9, 42.1, 76.5, 76.8, 78.1, 78.2, 95.2, 101.6, 103.8, 103.9, 115.1, 127.8, 127.9, 129.16, 129.19, 157.5, 160.5, 160.6, 161.5, 165.0, 169.76, 169.83, 196.67, 196.74 (one carbonyl carbon and six aryl carbons were duplicated). ESI-HR-MS $[M+Na]^+$ m/z: Calcd for $C_{22}H_{24}O_7Na$ 423.1420; Found 423.1414.

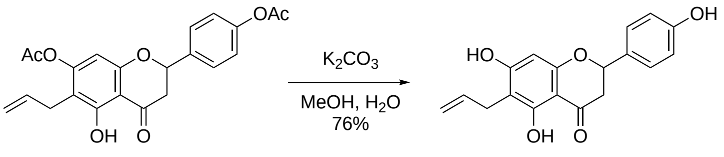

7 **2a**

6-Allyl-5,7-dihydroxy-2-(4-hydroxyphenyl)chroman-4-one (2a) [4, 5]

A same procedure for the preparation of **8-PN** using **7** (198 mg, 0.5 mmol) as a substrate was conducted

to give 118 mg (76%) of titled compound as a white powder of mp 196.0–197.0 °C. ^{1}H NMR (DMSO-*d6*, 500 MHz) δ 2.66 (dd, 1H, J = 3.2, 17.4), 3.16 (d, 2H, J = 6.0), 3.25 (dd, 1H, *J* = 13.3, 17.4), 4.88–4.93 (m, 2H), 5.40 (dd, 1H, *J* = 3.2, 13.3), 5.78–5.86 (m, 1H), 5.97 (s, 1H), 6.78 (d, 2H, *J* = 8.7), 7.30 (d, 2H, *J* = 8.7), 9.58 (br-s, 1H), 10.78 (br-s, 1H), 12.42 (s, 1H). ^{13}C NMR (DMSO-*d6*, 125 MHz) δ 25.7, 42.1, 78.4, 94.3, 101.5, 105.6, 114.3, 115.2, 128.3, 129.0, 136.1, 157.7, 160.8, 164.4, 196.5 (one aryl carbon was duplicated).

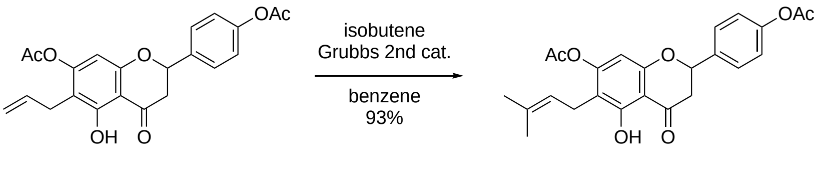

2-(4-Acetoxyphenyl)-5-hydroxy-6-(3-methylbut-2-enyl)-4-oxochroman-7-yl acetate (11) [2]

According to the similar literature procedure [2], to a solution of **7** (198 mg, 0.5 mmol) in 16 mL of dry benzene was added Grubbs 2nd generation catalyst (8.5 mg, 2 mol %) under an argon atmosphere. The mixture was cooled to –80 °C followed by the addition of isobutene (12 mL). After stirring the mixture in a sealed tube at room temperature for 2 days, the reaction mixture was concentrated in vacuo, and the residue was purified by column chromatography eluting with ethyl acetate and hexane in 1/8 to 1/3 ratio to give 197 mg of pale yellow solid. These processes were conducted twice to improve the yield and purity of the product. The compound **11** was used directly for the next step without further purification.

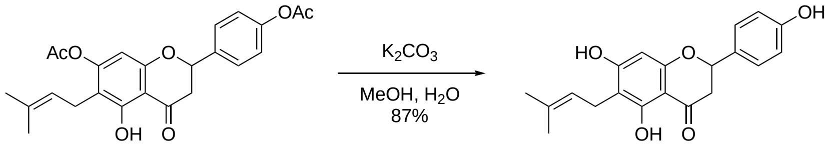

6-Prenylnaringenin (6-PN) [6,7]

A same procedure for the preparation of **8-PN** using **11** (297 mg, 0.7 mmol) as a substrate was conducted to give 207 mg (87%) of titled compound as a white powder. In order to clarify and prove the structural comparison of 8-PN and 6-PN, ^{1}H NMR was measured using two deuterated solvents (DMSO-*d6* and $CDCl_3$). The ^{1}H NMR spectrum of 6-PN using DMSO-*d6* was completely agreement with the values

reported in the reference [5].¹H NMR (DMSO-d_6, 500 MHz) δ 1.60 (s, 3H), 1.68 (s, 3H), 2.66 (dd, 1H, *J* = 3.2, 17.0), 3.10 (d, 2H, *J* = 7.5), 3.22 (dd, 1H, *J* = 13.2, 17.0), 5.11 (t, 1H, *J* = 7.5), 5.39 (dd, 1H, *J* = 3.2, 13.2), 5.94 (s, 1H), 6.78 (d, 2H, *J* = 8.5), 7.29 (d, 2H, *J* = 8.5), 9.58 (s, 1H), 10.75 (br-s, 1H), 12.41 (s, 1H). ^{1}H NMR ($CDCl_3$, 500 MHz) δ 1.77 (s, 3H), 1.82 (s, 3H), 2.78 (dd, 1H, *J* = 3.0, 17.5), 3.08 (dd, 1H, *J* = 13.5, 17.5), 3.36 (d, 2H, *J* = 7.5), 4.89 (s, 1H), 5.25 (t, 1H, *J* = 7.5), 5.33 (dd, 1H, *J* = 3.0, 13.5), 5.99 (s, 1H), 6.13 (s, 1H), 6.88 (d, 2H, *J* = 8.5), 7.33 (d, 2H, *J* = 8.5), 12.41 (s, 1H).

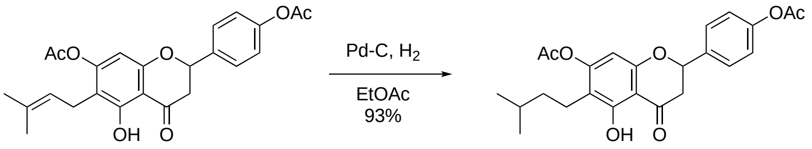

11 **12**

Acetic acid 4-[7-acetoxy-5-hydroxy-6-(3-methyl-butyl)-4-oxo-chroman-2-yl]-phenyl ester (12) [8]

A same procedure for the preparation of **1c** using **11** (318 mg, 0.75 mmol) as a substrate was conducted to give 297 mg (93%) of titled compound as white solid. ^{1}H NMR ($CDCl_3$, 500 MHz) δ 0.94 (d, 6H, *J* = 7.5), 1.36 (dd, 2H, *J* = 7.0, 11.0), 1.55–1.61 (m, 1H), 2.19 (s, 6H), 2.48 (t, 2H, *J* = 11.0), 2.86 (dd, 1H, *J* = 3.0, 17.3), 3.09 (dd, 1H, *J* = 13.0, 17.3), 5.43 (dd, 1H, J = 3.0, 13.0), 6.27 (s, 1H), 7.16 (d, 2H, *J* = 8.5), 7.47 (d, 2H, *J* = 8.5), 12.15 (s, 1H).

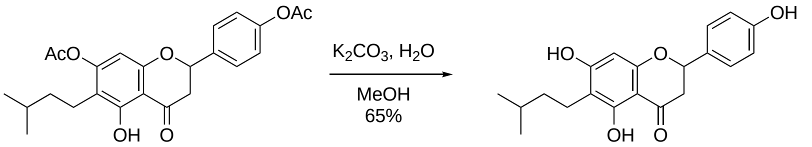

12 **2b**

5,7-Dihydroxy-2-(4-hydroxy-phenyl)-6-(3-methyl-butyl)-chroman-4-one (2b) [8]

A same procedure for the preparation of **8-PN** using **12** (212 mg, 0.5 mmol) as a substrate was conducted to give 138 mg (65%) of titled compound as a white powder. ^{1}H NMR (DMSO-*d6*, 500 MHz) δ 0.87 (d, 6H, *J* =6.5), 1.28 (dd, 2H, *J* = 6.5, 11.0), 1.46–1.52 (m, 1H), 2.42 (t, 2H, J = 11.0) 2.64 (dd, 1H, *J* = 3.0, 17.0), 3.23 (dd, 1H, *J* = 12.5, 17.0), 5.39 (dd, 1H, *J* = 3.0, 12.5), 5.95 (s, 1H), 6.78 (d, 2H, *J* = 9.0), 7.30 (d, 2H, 9.0), 9.58 (br-s, 1H), 10.66 (br-s, 1H), 12.41 (s, 1H).

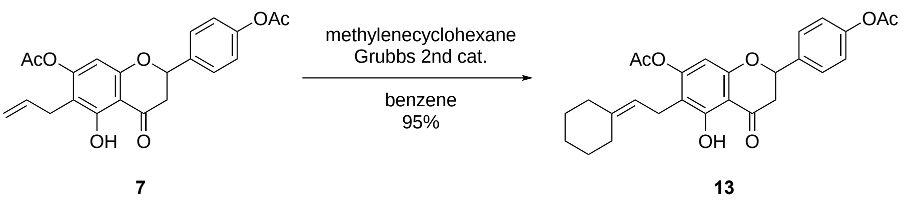

Acetic acid 4-[7-acetoxy-6-(2-cyclohexylidene-ethyl)-5-hydroxy-4-oxo-chroman-2-yl]-phenyl ester (13)

According to the similar literature procedure [2], to a solution of **7** (198 mg, 0.5 mmol) in 16 mL of dry benzene was added Grubbs 2nd generation catalyst (12.1 mg, 3 mol %) under an argon atmosphere. The mixture was cooled under ice water bath followed by the addition of methylenecyclohexane (1.8 mL). After stirring the mixture in a sealed tube at room temperature for 3 days, the reaction mixture was concentrated in vacuo, and the residue was purified by flash column chromatography eluting with ethyl acetate and hexane in 1/4 ratio to give pale yellow solid. These processes were conducted twice to improve the yield and purity of the product. The solid was recrystallized from ethyl acetate and hexane to give 189 mg (95%) of titled compound as white powder of mp 111.6–112.6 °C. ^{1}H NMR ($CDCl_3$, 500 MHz) δ 1.54–1.56 (m, 6H), 2.04 (t, 2H, *J* = 5.5), 2.28 (t, 2H, *J* = 5.5), 2.32 (s, 6H), 2.86 (dd, 1H, *J* = 2.5, 17.3), 3.09 (dd, 1H, *J* = 13.5, 17.3), 3.22 (d, 2H, *J* = 6.5), 5.06 (t, 1H, *J* = 6.5), 5.43 (dd, 1H, *J* = 2.5, 13.5), 6.28 (s, 1H), 7.16 (d, 2H, *J* = 8.5), 7.46 (d, 2H, *J* = 8.5), 12.14 (s, 1H). ^{13}C NMR (DMSO-*d6*, 125 MHz) δ 20.6, 20.7, 20.9, 26.3, 27.3, 28.0, 28.2, 36.5, 78.1, 102.2, 105.7, 114.1, 117.9, 122.1, 128.0, 135.9, 139.2, 150.6, 156.0, 159.4, 160.2, 168.3, 169.2, 198.3. ESI-HR-MS $[M+H]^+$ m/z: Calcd for $C_{27}H_{29}O_7$ 465.1908; Found 465.1915.

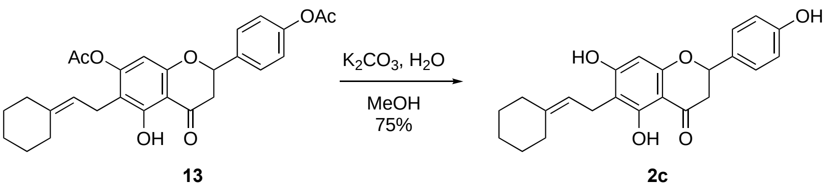

6-(2-Cyclohexylidene-ethyl)-5,7-dihydroxy-2-(4-hydroxy-phenyl)-chroman-4-one (2c)

A same procedure for the preparation of **8-PN** using **13** (140 mg, 0.3 mmol) as a substrate was conducted to give 105 mg (75%) of titled compound as white powder of mp 204.4–205.2 °C. ^{1}H NMR (DMSO-*d6*, 500 MHz) δ 1.42–1.48 (m, 6H), 1.97 (t, 2H, *J* = 6.0), 2.24 (t, 2H, *J* = 5.5), 2.66 (dd, 1H, *J* = 3.0, 17.0), 3.11 (d, 2H, *J* = 7.5), 3.22 (dd, 1H, *J* = 12.5, 17.0), 5.05 (t, 1H, J = 7.5), 5.39 (dd, 1H, *J* = 3.0, 12.5), 5.94 (s, 1H), 6.78 (d,

2H, *J* = 8.5), 7.29 (d, 2H, *J* = 8.5), 9.57 (br–s, 1H), 10.73 (br–s, 1H), 12.40 (br–s, 1H). ^{13}C NMR (DMSO-*d6*, 125 MHz) δ 19.7, 26.4, 27.3, 28.3, 36.5, 42.1 78.3, 94.3, 101.6, 107.6, 115.1, 119.1, 128.3, 129.0, 138.1, 157.7, 160.5, 160.6, 164.2, 196.5. ESI-HR-MS [M+H]+ m/z: Calcd for $C_{23}H_{23}O_5$ 379.1551; Found 379.1560.

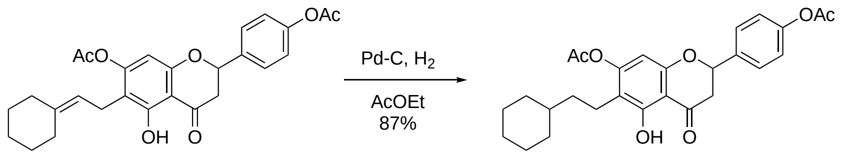

13 14

Acetic acid 4-[7-acetoxy-6-(2-cyclohexyl-ethyl)-5-hydroxy-4-oxo-chroman-2-yl]-phenyl ester (14)

A same procedure for the preparation of **1c** using **13** (116 mg, 0.25 mmol) as a substrate was conducted to give 102 mg (87%) of titled compound (conformational isomers) as white powder of mp 115.1–117.1 °C. ^1H NMR ($CDCl_3$, 500 MHz) δ 0.90–0.97 (m, 2H), 1.15–1.26 (m, 4H), 1.34–1.39 (m 2H), 1.65–1.67 (m,1H), 1.70–1.72 (m, 2H), 1.78 –1.80 (m, 2H), 2.33 (s, 6H), 2.49 (t, 2H, *J* = 8.0), 2.87 (dd, 1H, *J* = 3.0, 17.0), 3.10 (dd, 1H, *J* = 13.5, 17.0), 5.44 (dd, 1H, *J* = 3.0, 13.5), 6.28 (s, 1H,), 7.17 (d, 2H, *J* = 8.5),7.47 (d, 2H, *J* = 8.5), 12.15 (s, 1H). ^{13}C NMR (DMSO-*d6*, 125 MHz) δ 19.8, 20.2, 20.6, 20.8, 25.8, 26.1, 26.2, 32.68, 32.72, 32.8, 36.1, 36.4, 36.6. 36.9, 42.4, 78.03, 78.08, 102.1, 103.4, 105.7, 106.0, 114.2, 115.2, 122.0, 127.5, 127.9, 128.0, 135.9, 136.1, 150.5, 150.6, 156.12, 156.19, 159.3, 159.5, 159.8, 160.3, 168.4, 168.5, 169.2, 198.2, 198.3 (four carbons were duplicated). ESI-HR-MS [M+H]+ m/z: Calcd for $C_{27}H_{31}O_7$ 467.2064; Found 467.2069.

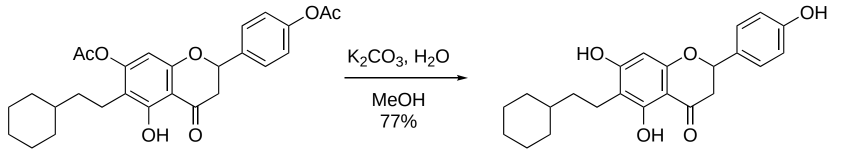

14 2d

6-(2-Cyclohexyl-ethyl)-5,7-dihydroxy-2-(4-hydroxy-phenyl)-chroman-4-one (2d)

A same procedure for the preparation of **8-PN** using **14** (140 mg, 0.3 mmol) as a substrate was conducted to give 108 mg (77%) of titled compound (conformational isomers) as white powder of mp 213.3–215.3 °C. ^1H NMR (DMSO-*d6*, 500 MHz) δ 0.82–0.90 (m, 2H), 1.09–1.21 (m, 2H), 1.26–1.60 (m, 4H), 1.63–1.75(m, 2H), 1.90– 1.98 (m, 2H), 2.39–2.44 (m, 1H), 2.62 (dd, 1H, *J* = 3.0, 17.0), 3.23 (dd, 1H, *J* = 13.0, 17.0), 5.39 (dd, 1H, *J* = 3.0, 13.0), 5.94 (s, 1H), 6.77 (d, 2H, *J* = 8.5), 7.29 (d, 2H, *J* = 8.5), 9.58 (br–s, 1H), 10.68 (br–

s, 1H), 12.40, 12.41 (s, 1H). ^{13}C NMR (DMSO-*d6*, 125 MHz) δ 14.0, 18.9, 20.2, 21.7, 22.1, 22.6, 23.5, 24.7, 25.8, 26.2, 27.9, 32.8, 36.2, 36.6, 37.1, 42.1, 78.3, 94.3, 101.5, 108.1, 108.6, 115.1, 120.1, 128.3, 129.0, 137.5, 157.7, 160.4, 160.5, 160.7, 160.8, 164.4, 196.5 (six carbons were duplicated). ESI-HR-MS $[M+H]^+$ m/z: Calcd for $C_{23}H_{25}O_5$ 381.1707; Found 381.1715.

Results

^1H NMR (500 MHz) and ^{13}C NMR (125 MHz) spectra of our synthesized samples were recorded using a JEOL JNM-ECP500 spectrometer and the data are shown below. Chemical shift values are expressed in ppm relative to the solvent residual signals of $CDCl_3$ (7.26 ppm), DMSO-*d6* (2.50 ppm) in ^1H NMR, and $CDCl_3$ (77.1 ppm), DMSO-*d6* (39.5 ppm) in ^{13}C NMR. TMS (tetramethylsilane) was used as an internal standard in case of using $CDCl_3$. Abbreviations: s, singlet; d, doublet; t, triplet; m, multiplet; br, broad. The coupling constant values are expressed in hertz. High resolution mass spectrometry (HR-MS) was performed using an LTQ-Orbitrap XL Mass Spectrometer (Thermo Fisher Scientific, Inc. Waltham, MA, USA) or LCMS-9030 mass spectrometer (Shimadzu, Kyoto, Japan). Melting points were measured using a Yanaco micro melting point apparatus, without correction.

Figure S1. ^{1}H NMR chart of compound **3**. (DMSO-*d6*, 500 MHz).

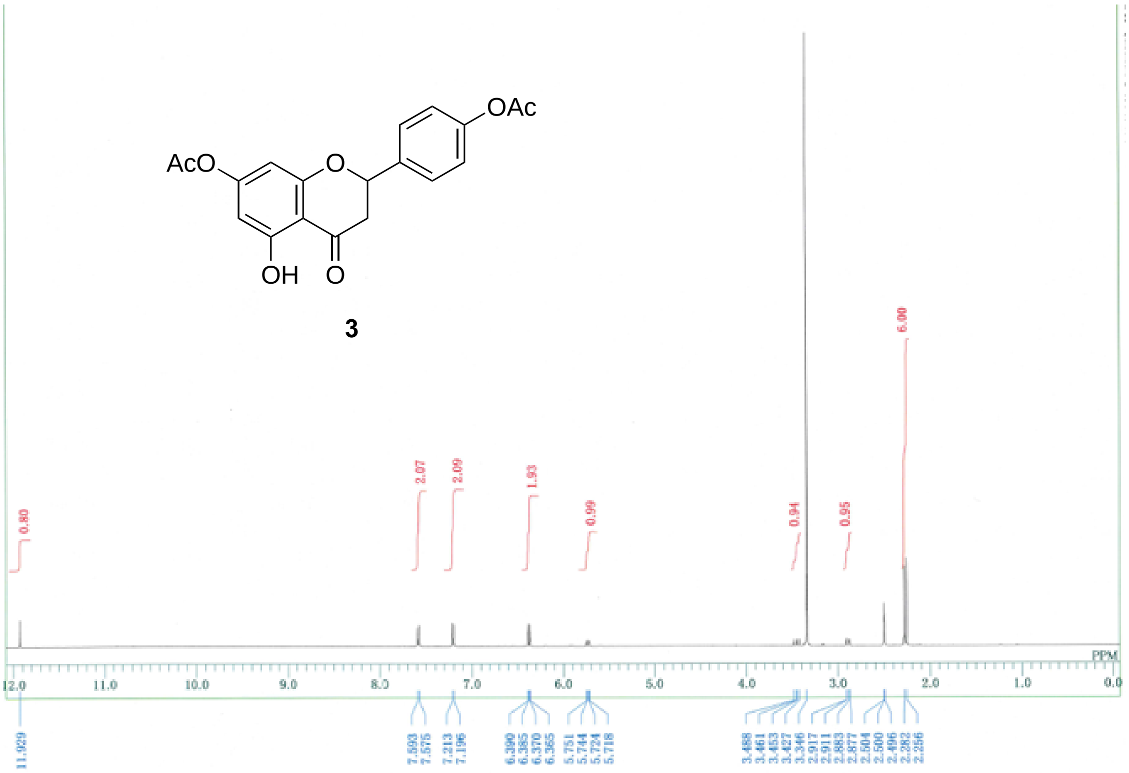

Figure S2 ^{1}H NMR chart of compound **4**. ($CDCl_3$, 500 MHz, internal standard: TMS)

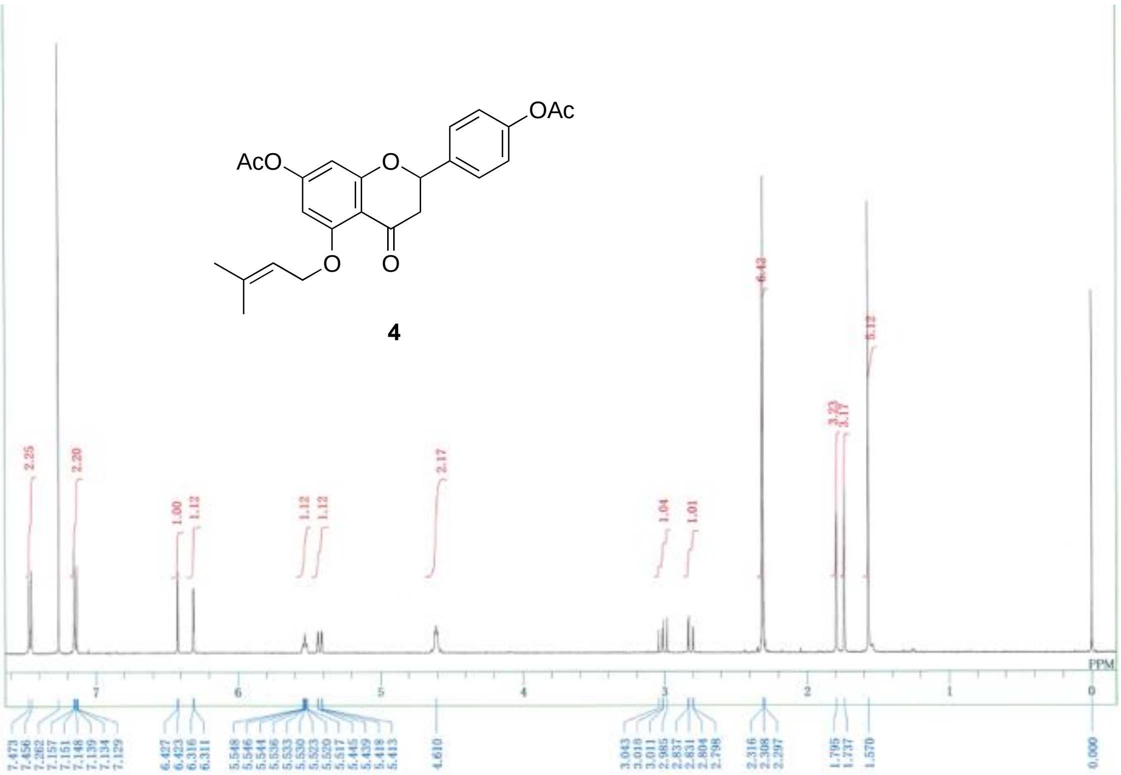

Figure S3 ^{1}H NMR chart of compound **5**. ($CDCl_3$, 400 MHz, internal standard: TMS)

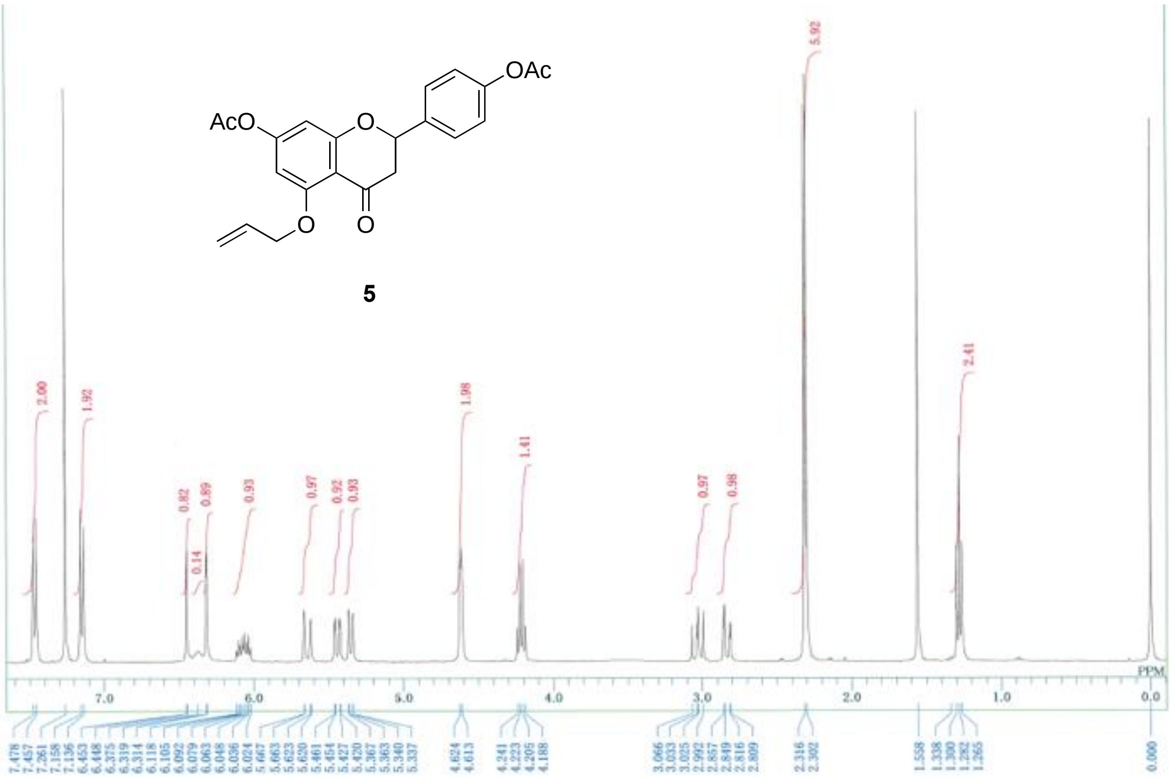

Figure S4 ^{1}H NMR chart of compound **7**. ($CDCl_3$, 500 MHz, internal standard: TMS)

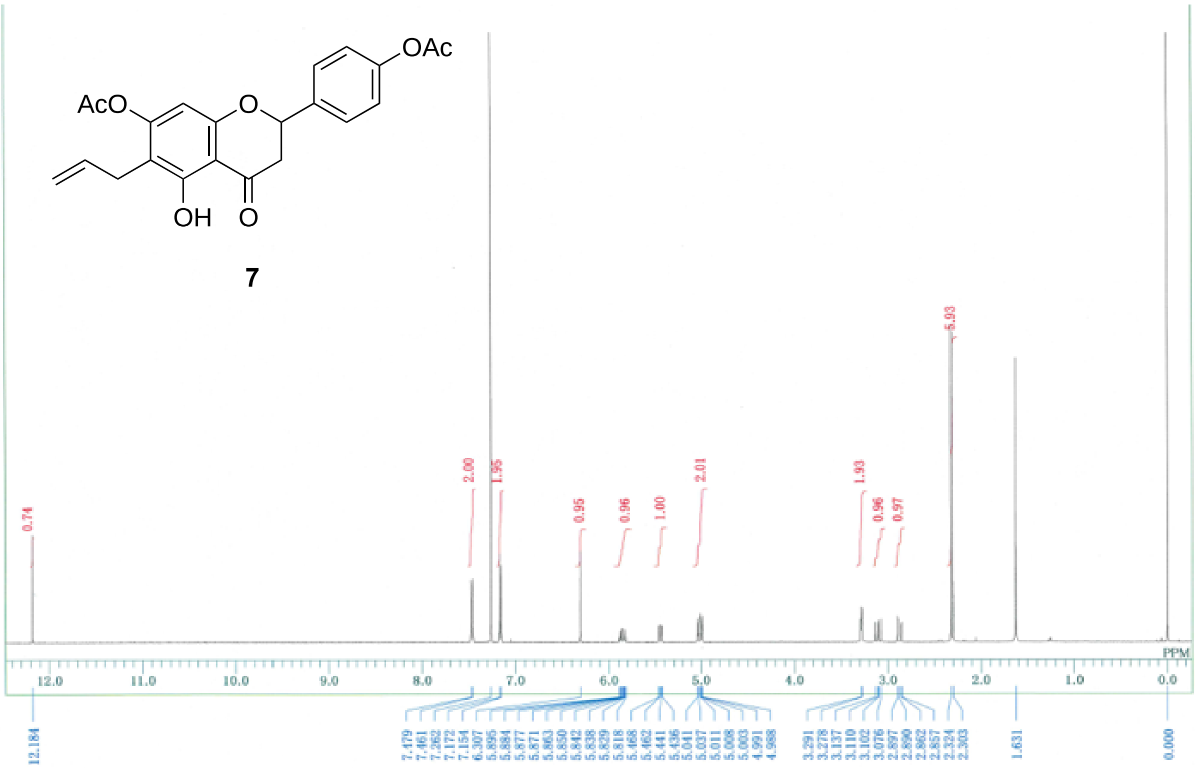

Figure S5a ^{1}H NMR chart of compound **8-PN**. (DMSO-*d6*, 500 MHz)

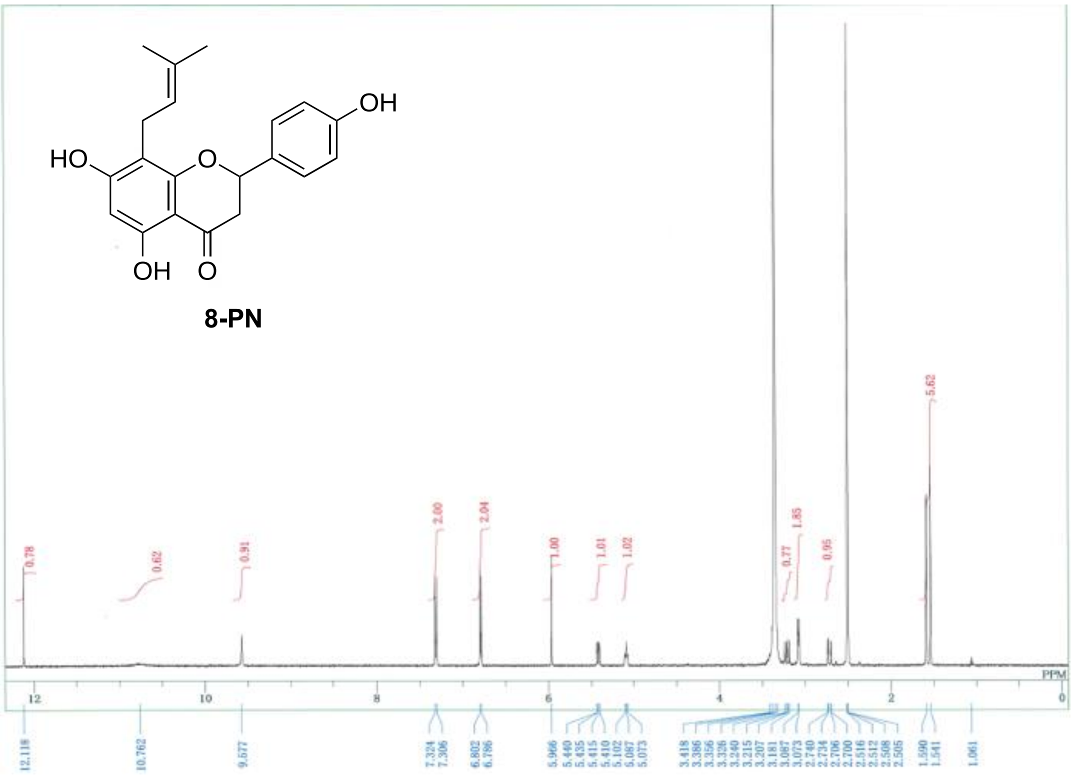

Figure S5b ^{1}H NMR chart of compound **8-PN**. ($CDCl_3$, 500 MHz, internal standard: TMS)

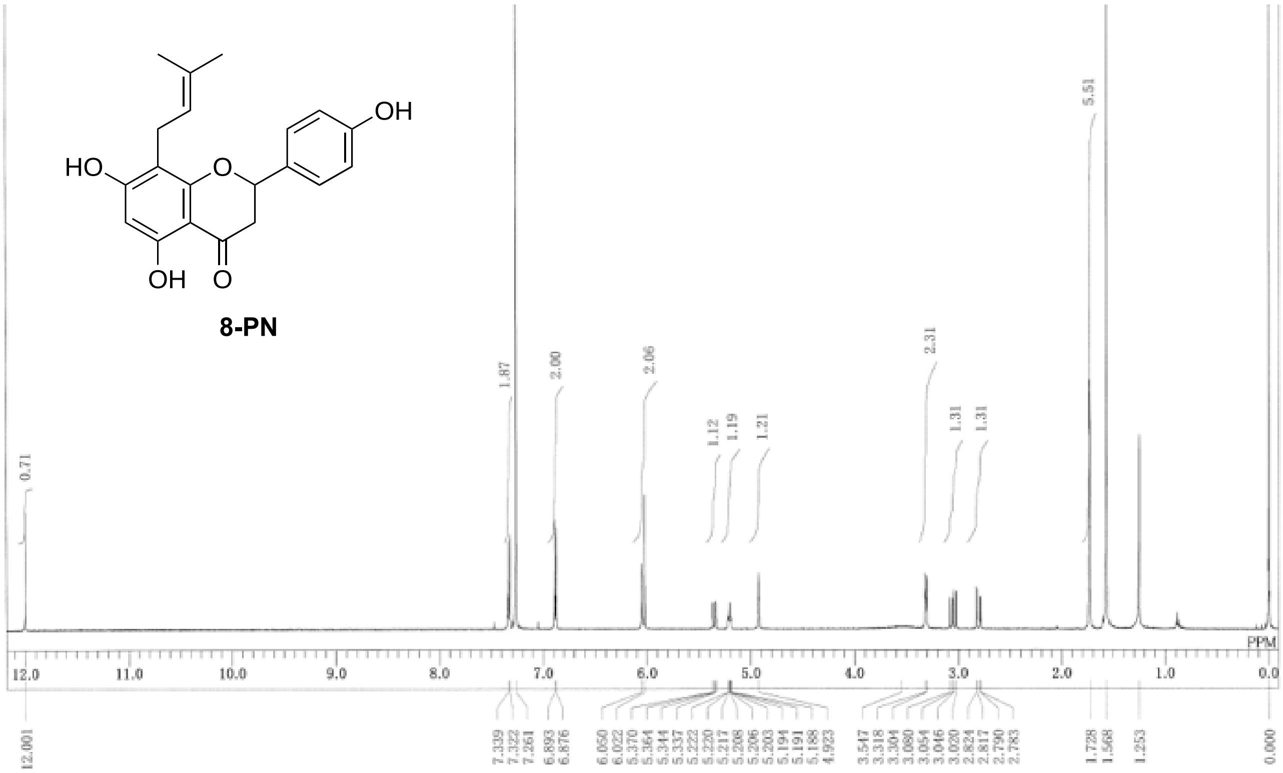

Figure S6 ^{1}H NMR chart of compound **1c**. ($CDCl_3$, 500 MHz, internal standard: TMS)

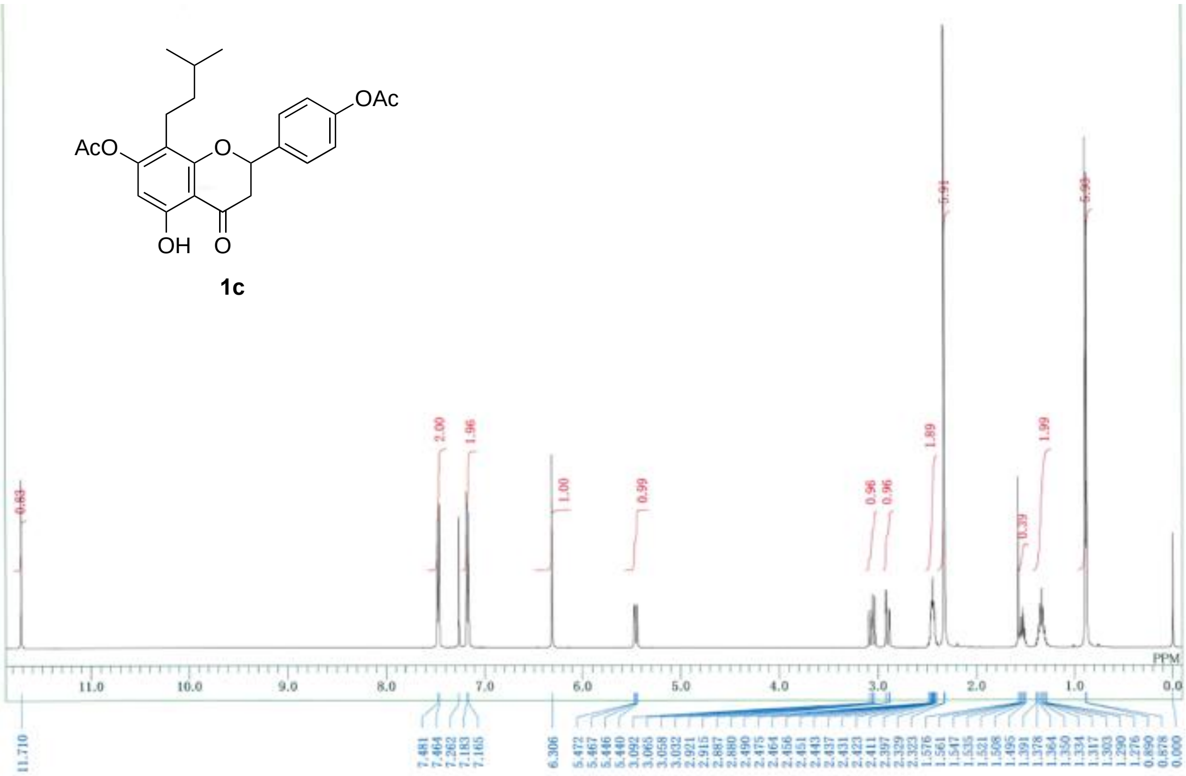

Figure S7 ^{13}C NMR chart of compound **1c**. ($CDCl_3$, 125 MHz, internal standard: TMS)

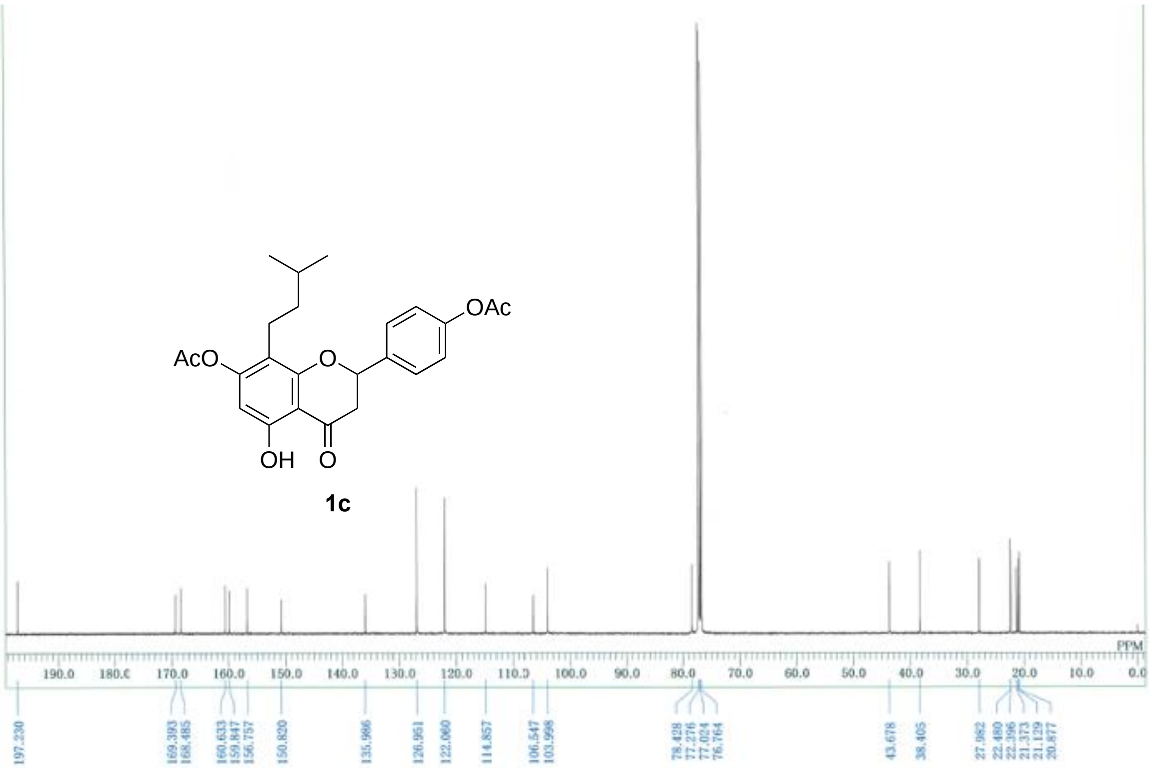

Figure S8 ESI-HR-MS chart of compound **1c**. [M+Na]$^+$ m/z: Calcd for $C_{24}H_{26}O_7Na$ 449.1576; Found 449.1569.

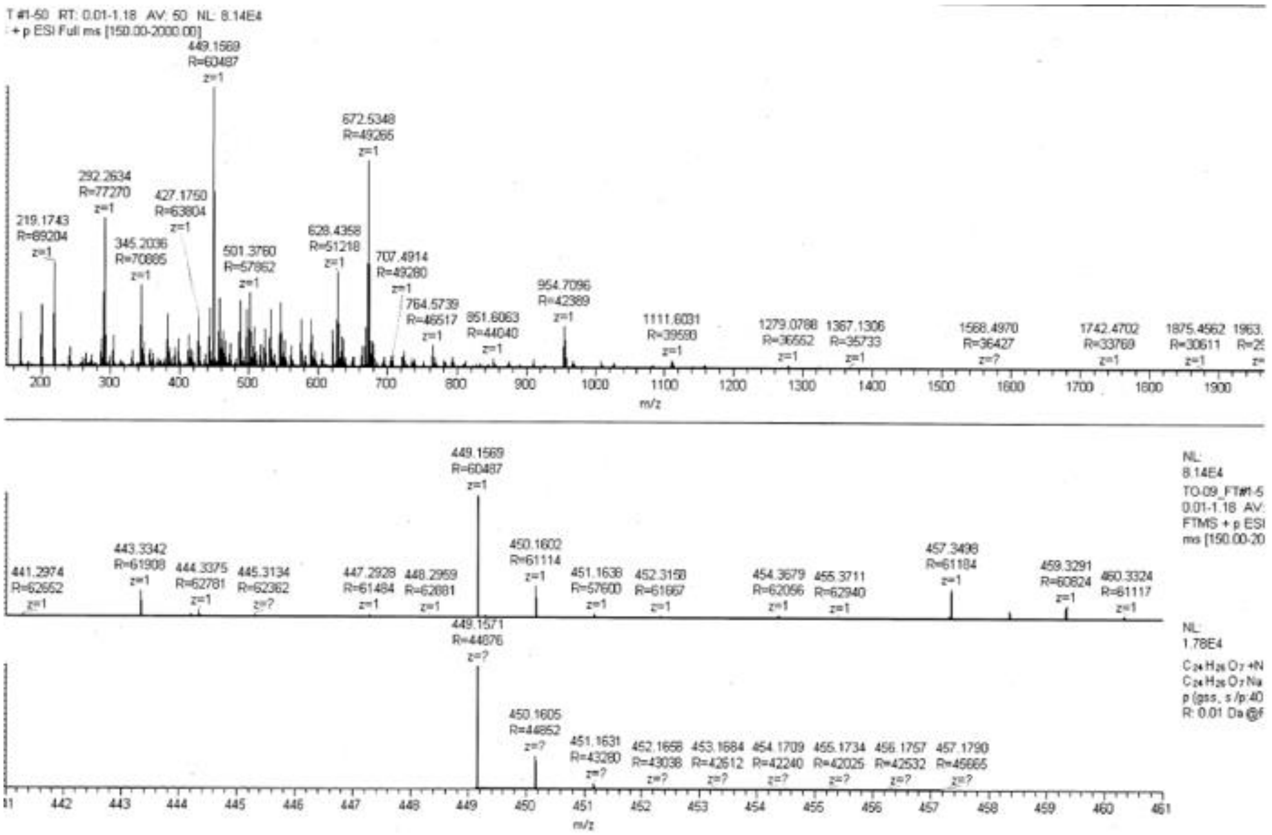

target m/z 449.1569

Calculated results

Idx.	Formula	RDB	Delta ppm
1	C24 H26 O7 Na	11.5	−0.343

Bounding conditions

Charge		1
Nitrogen Rule	None	
Mass tolerance		5 ppm
RDB equiv.		−1 100

Elements in use

16 O	0	15	0	15.995
12 C	0	50	1	12
1 H	0	50	−0.5	1.008
23 Na	0	1	−0.5	22.99

Figure S9 ^{1}H NMR chart of compound **1d**. (DMSO-*d6*, 500 MHz)

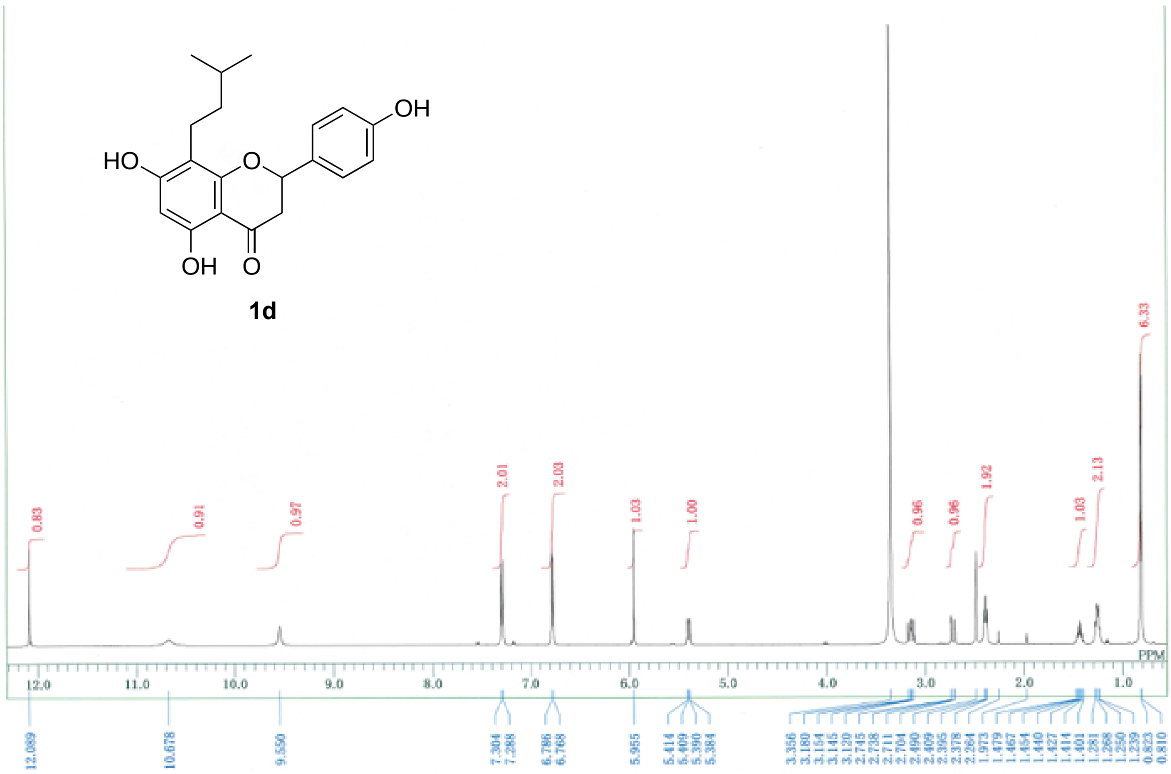

Figure S10 ^{13}C NMR chart of compound **1d**. (DMSO-*d6*, 125 MHz)

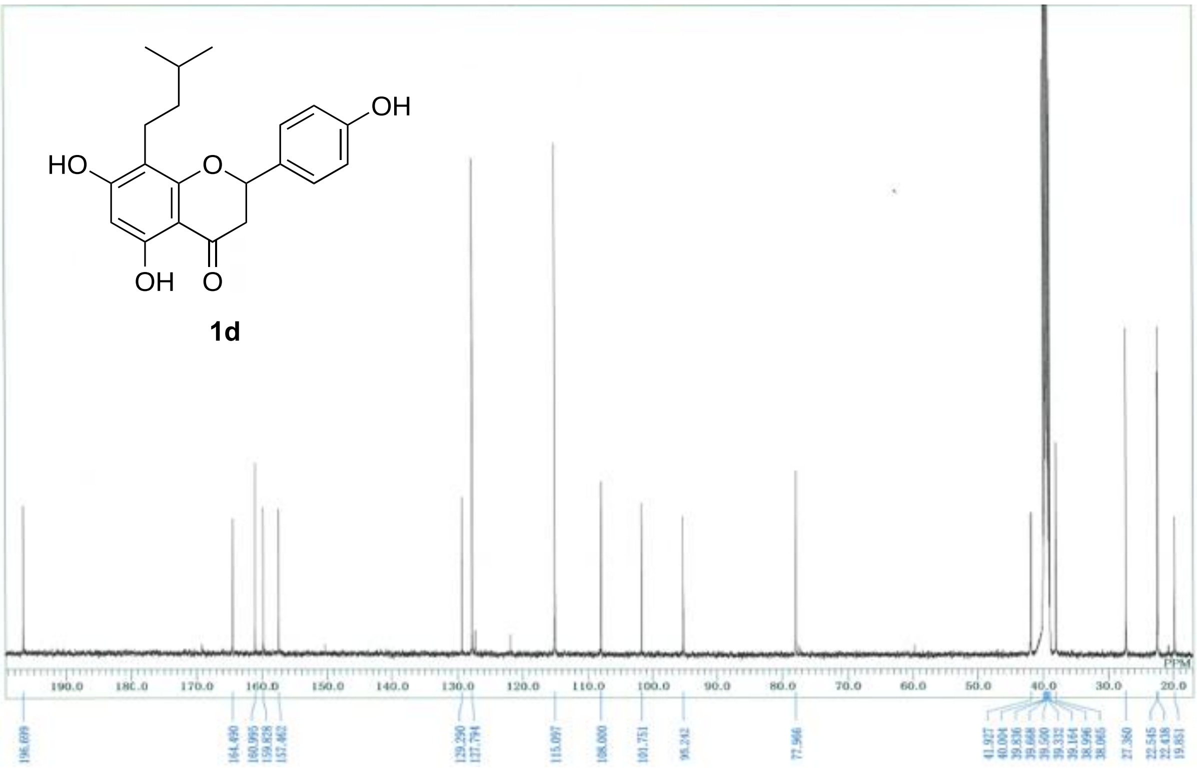

Figure S11 ESI-HR-MS chart of compound **1d**. $[M+H]^+$ m/z: Calcd for $C_{20}H_{23}O_5$ 343.1545; Found 343.1540.

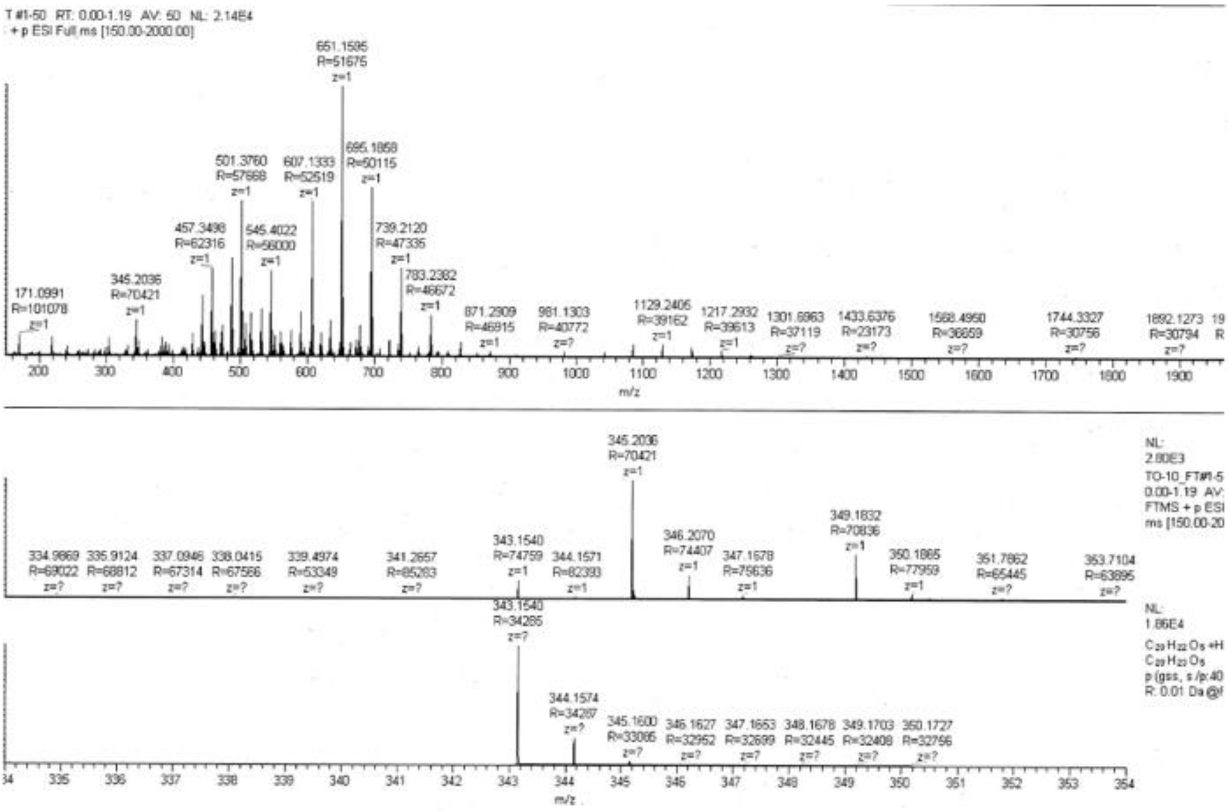

target m/z	343.1540

Calculated results

Idx.	Formula	RDB	Delta ppm
1	C20 H23 O5	9.5	0.087

Bounding conditions

Charge		1	
Nitrogen Rule	None		
Mass tolerance		5 ppm	
RDB equiv.		−1 _100	

Elements in use

16 O	0	15	0	15.995
12 C	0	50	1	12
1 H	0	50	−0.5	1.008
23 Na	0	1	−0.5	22.99

Figure S12 ^{1}H NMR chart of compound **8**. ($CDCl_3$, 500 MHz, internal standard: TMS)

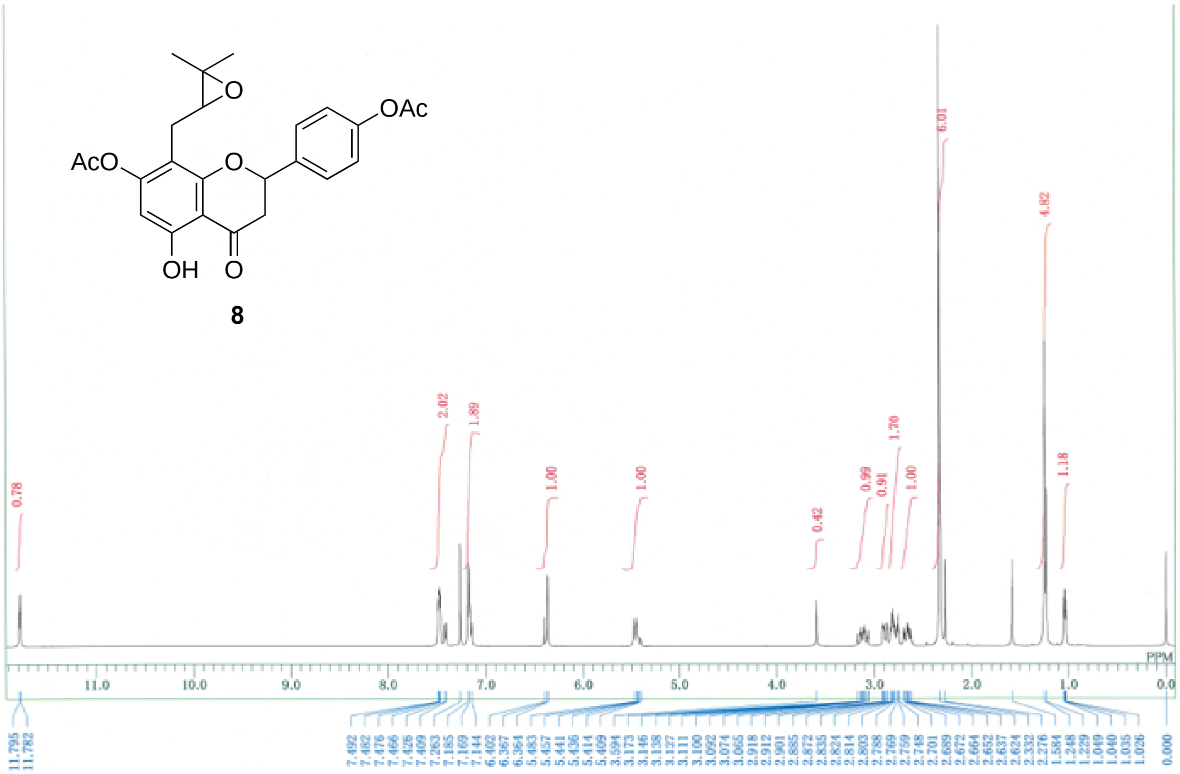

Figure S13 ^{13}C NMR chart of compound **8**. ($CDCl_3$, 125 MHz, internal standard: TMS)

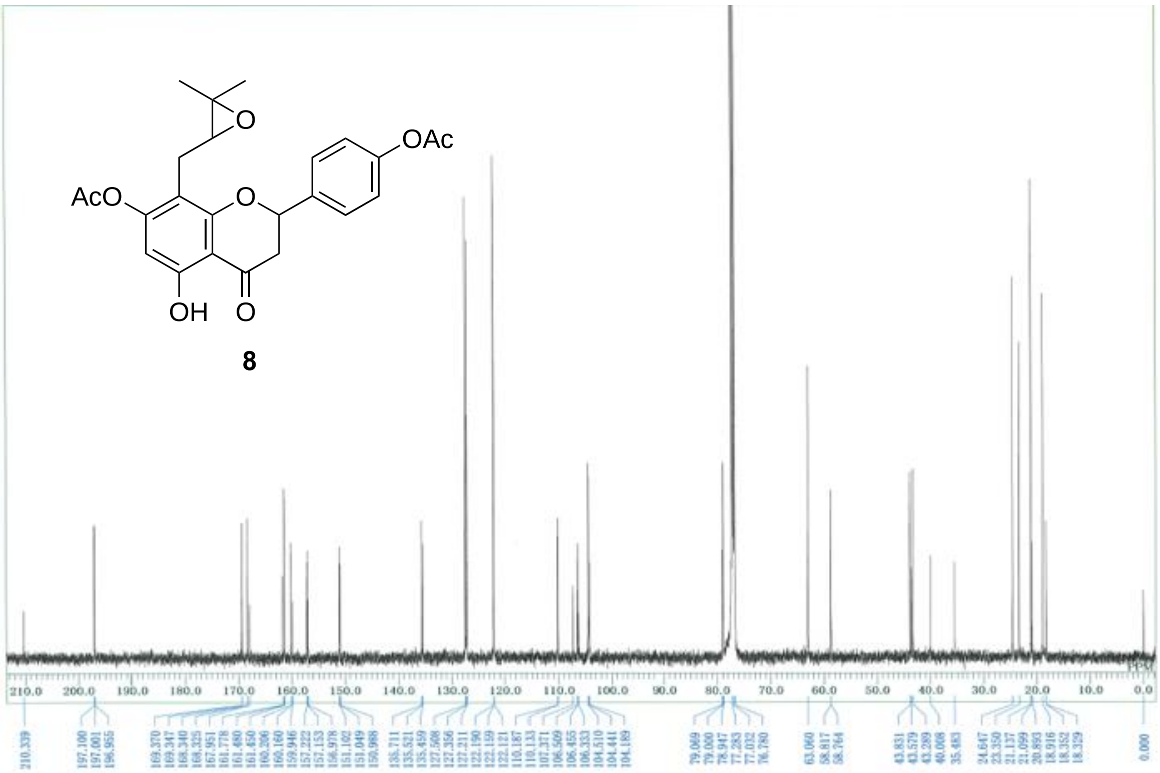

Figure S14. ESI-HR-MS chart of compound **8**. $[M+Na]^+$ m/z: Calcd for $C_{24}H_{24}O_8Na$ 463.1369; Found 463.1359.

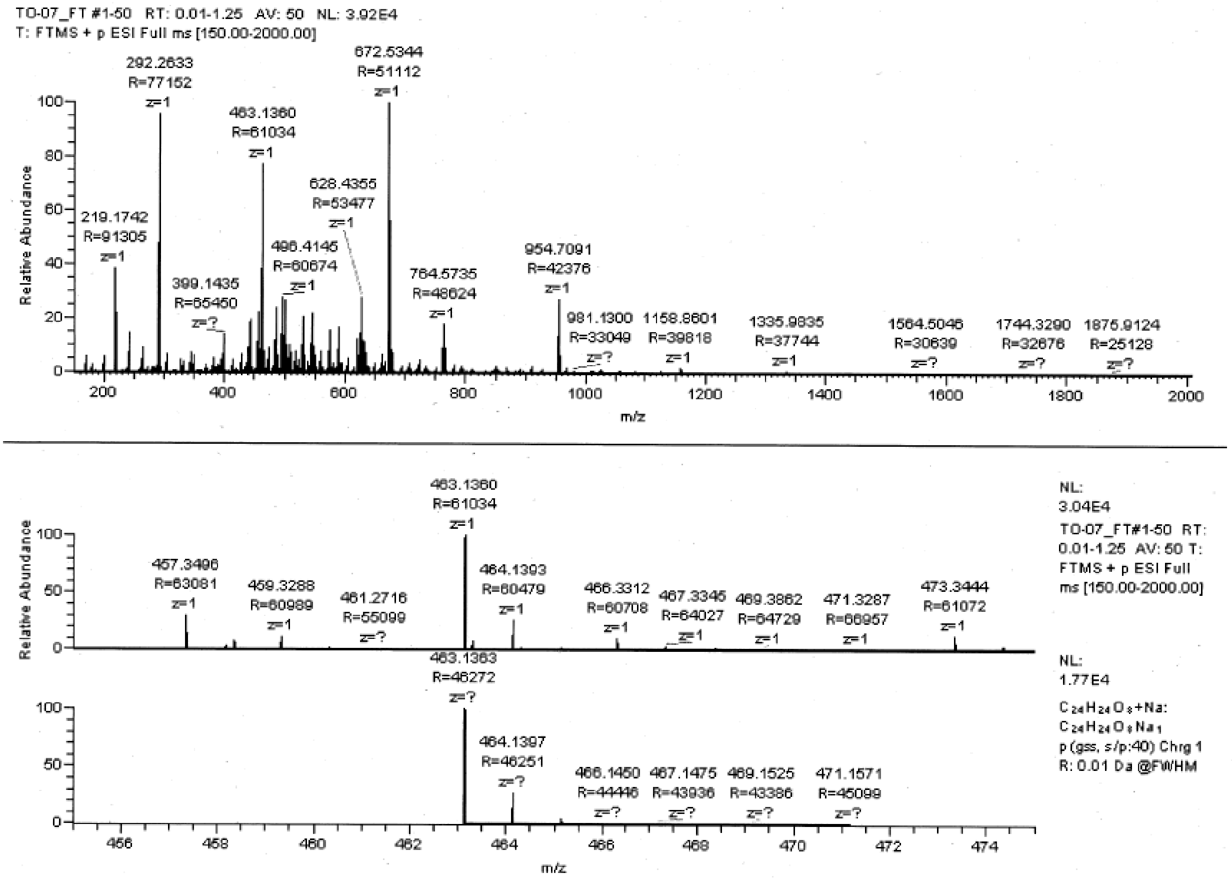

target m/z 463.1359

Calculated results

Idx.	Formula	RDB	Delta ppm
1	C24 H24 O8 Na	12.5	−0.775

Bounding conditions

Charge		1
Nitrogen Rule	None	
Mass tolerance		5 ppm
RDB equiv.		−1 _100

Elements in use

16 O	0	15	0	15.995
12 C	0	50	1	12
1 H	0	50	−0.5	1.008
23 Na	0	1	−0.5	22.99

Figure S15. ^{1}H NMR chart of compound **1a**. (DMSO-*d6*, 500 MHz)

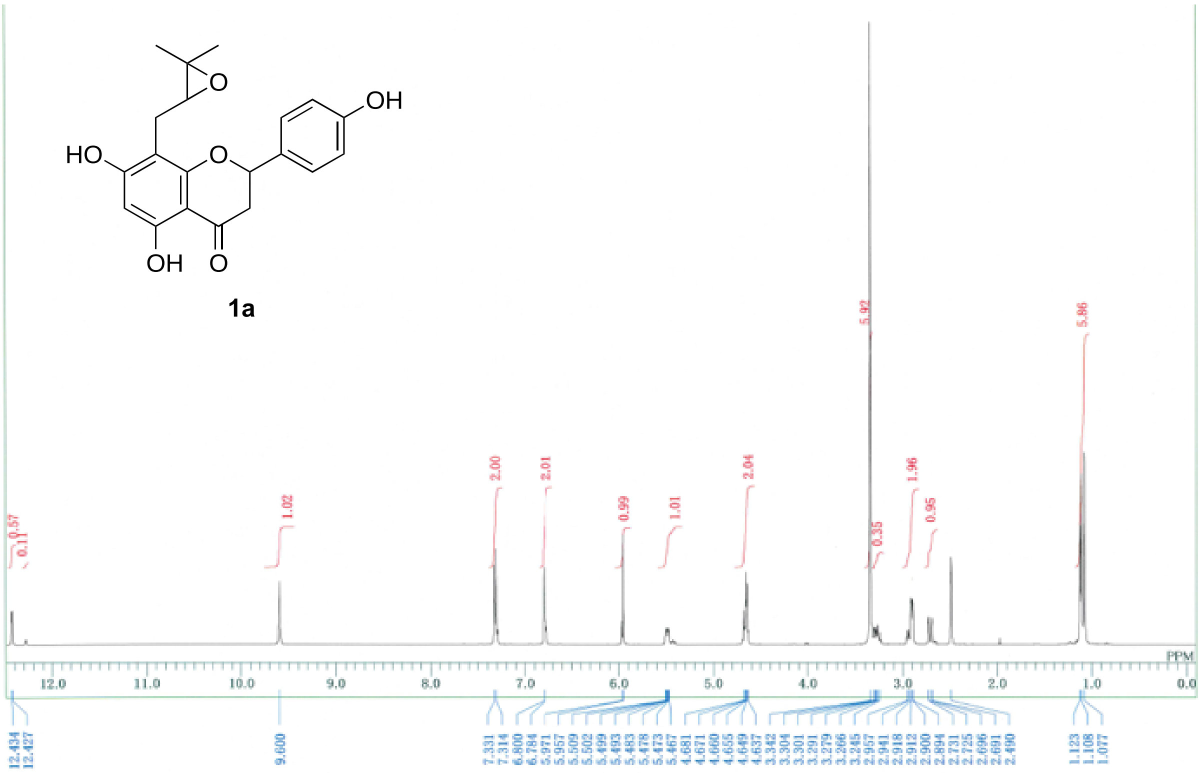

Figure S16. ^{13}C NMR chart of compound **1a**. (DMSO-*d6*, 125 MHz)

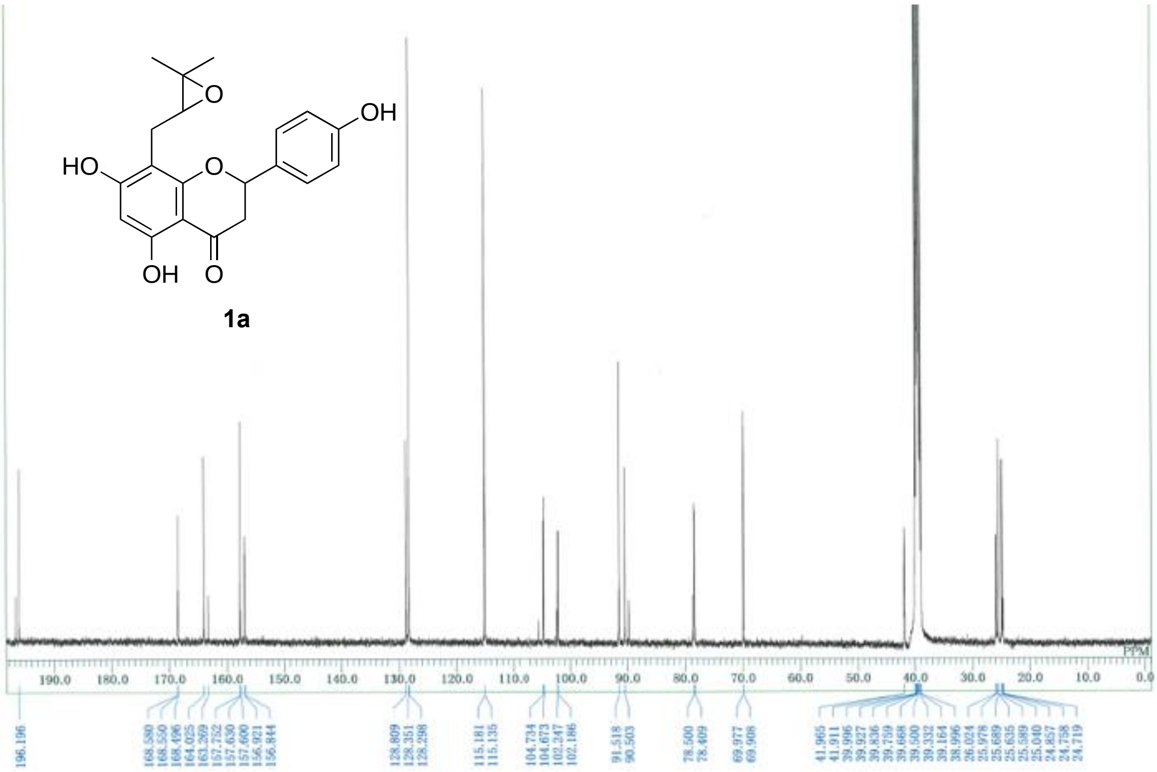

Figure S17. ESI-HR-MS chart of compound **1a**. $[M+Na]^+$ m/z: Calcd for $C_{20}H_{20}O_6Na$ 379.1158; Found 379.1153.

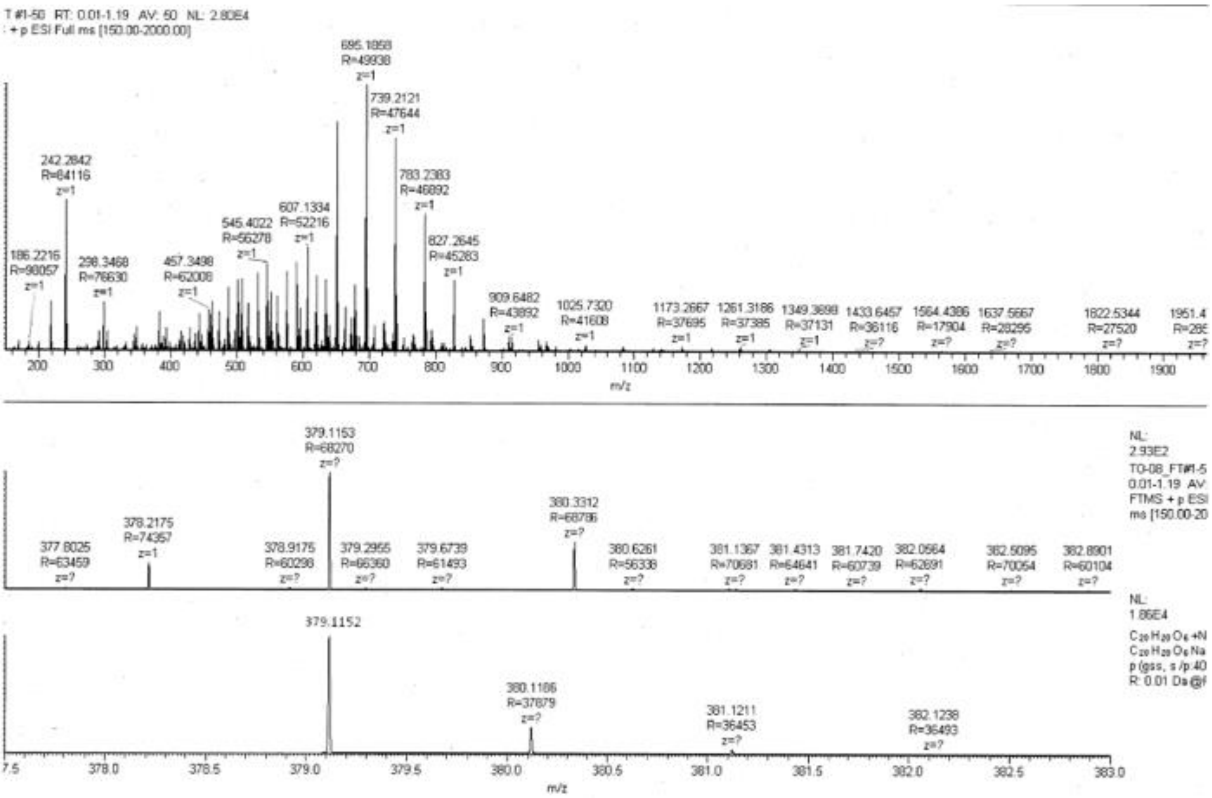

target m/z 379.1153

Calculated results

Idx.	Formula	RDB	Delta ppm
1	C20 H20 O6 Na	10.5	0.186

Bounding conditions

Charge		1
Nitrogen Rule	None	
Mass tolerance		5 ppm
RDB equiv.		−1 _100

Elements in use

16 O	0	15	0	15.995
12 C	0	50	1	12
1 H	0	50	−0.5	1.008
23 Na	0	1	−0.5	22.99

Figure S18. ^{1}H NMR chart of compound **1b**. (DMSO-*d6*, 500 MHz)

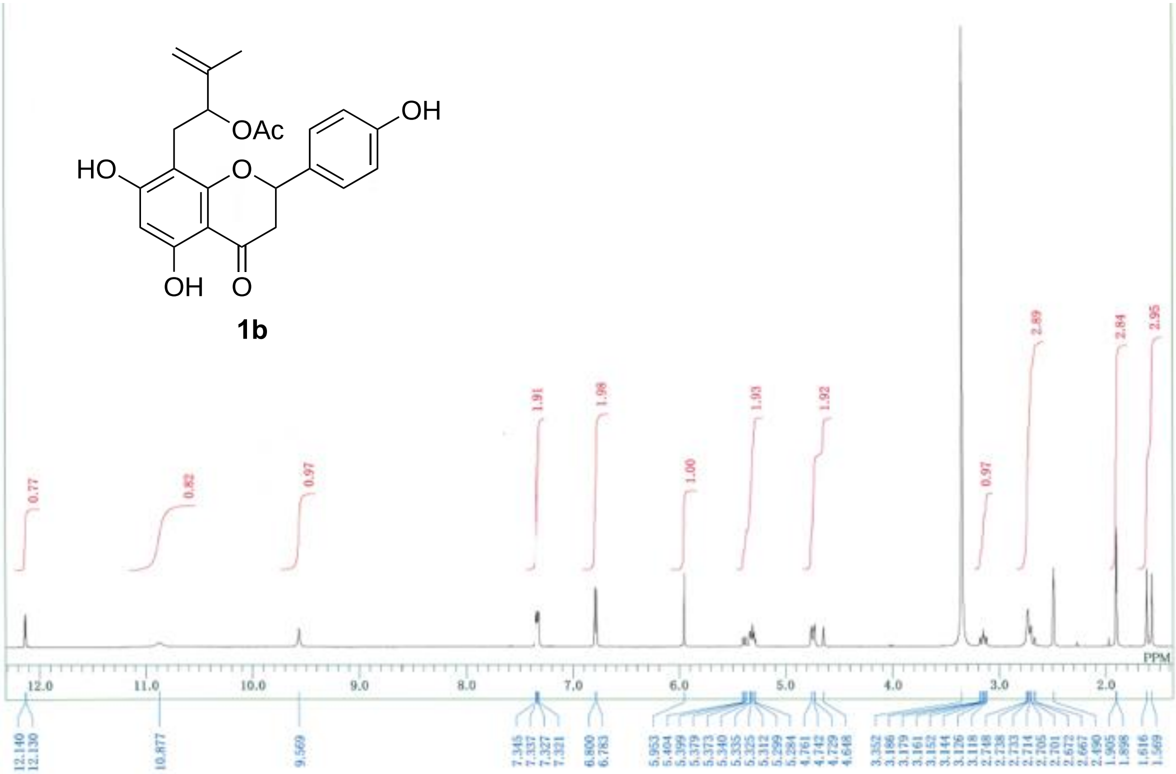

Figure S19. ^{13}C NMR chart of compound **1b**. (DMSO-*d6*, 125 MHz)

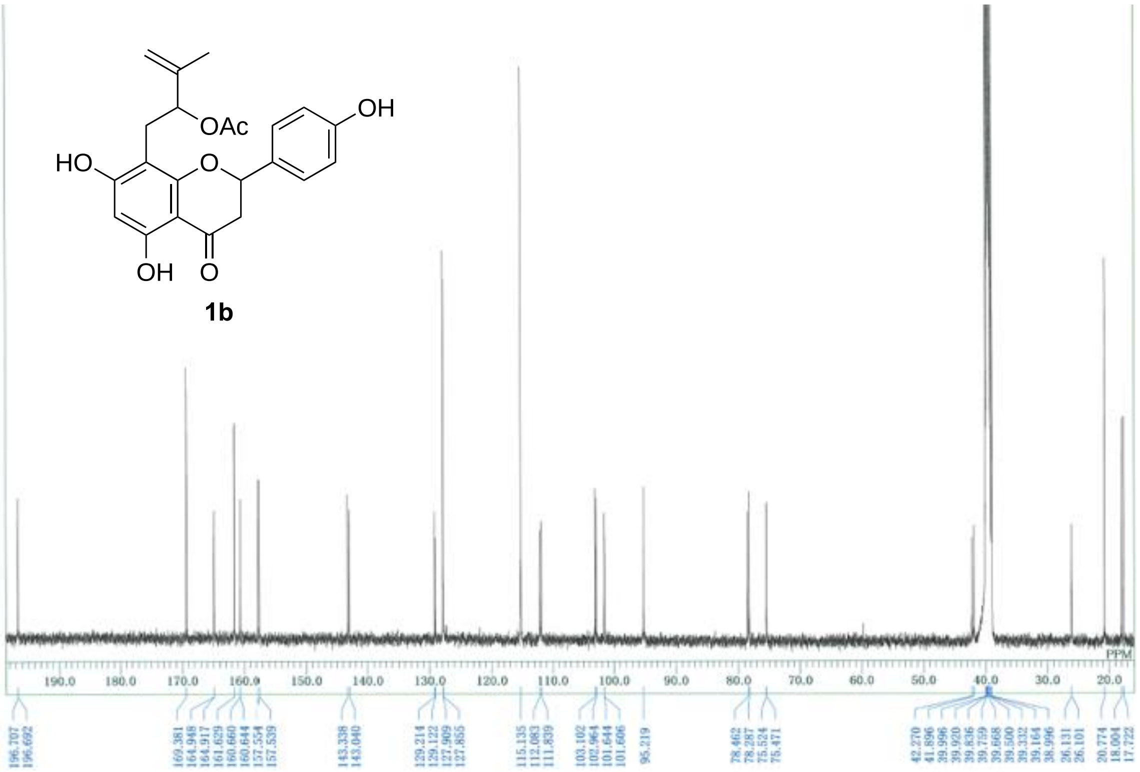

Figure S20. ESI-HR-MS chart of compound **1b**. $[M+Na]^+$ m/z: Calcd for $C_{22}H_{22}O_7Na$ 421.1263; Found 421.1257.

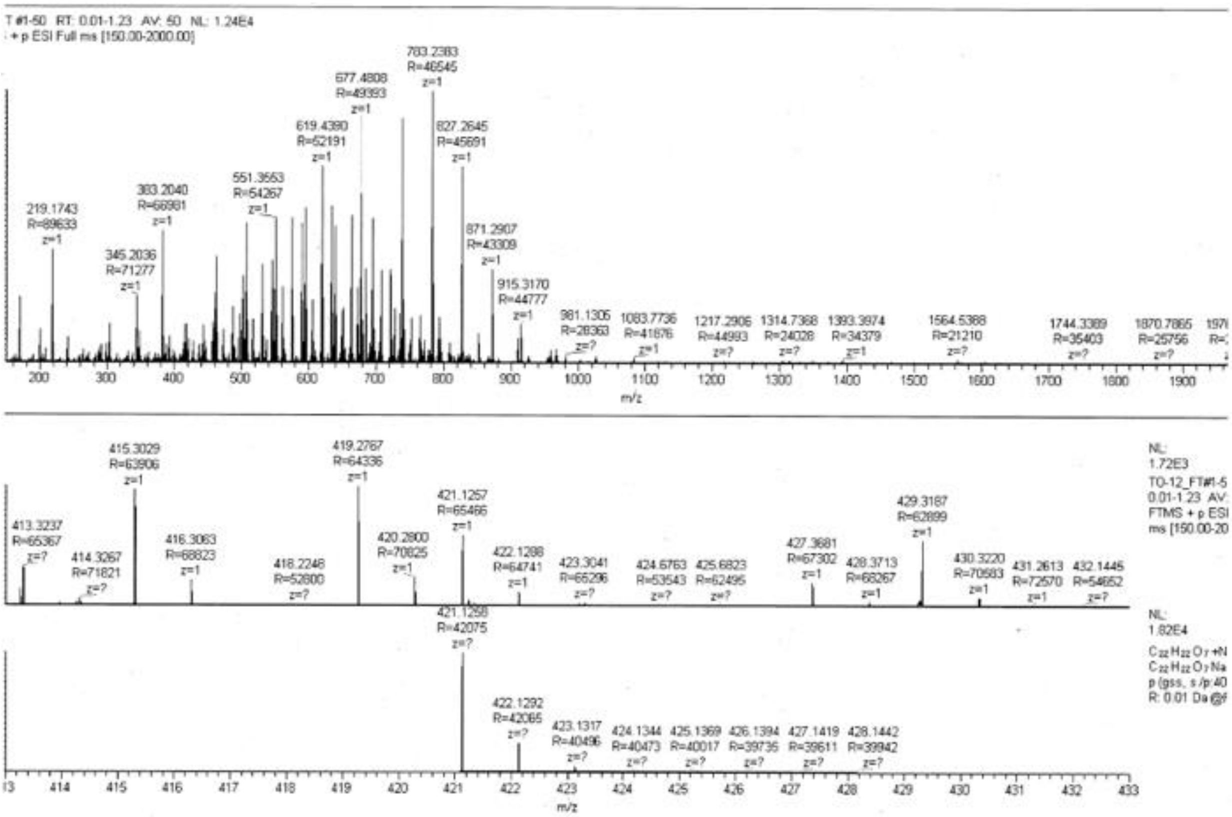

target m/z 421.1257

Calculated results

Idx.	Formula	RDB	Delta ppm
1	C22 H22 O7 Na	11.5	-0.224

Bounding conditions

Charge		1
Nitrogen Rule	None	
Mass tolerance		5 ppm
RDB equiv.		-1 _100

Elements in use

16 O	0	15	0	15.995
12 C	0	50	1	12
1 H	0	50	-0.5	1.008
23 Na	0	1	-0.5	22.99

Figure S21. ^{1}H NMR chart of compound **10**. ($CDCl_3$, 500 MHz, internal standard: TMS)

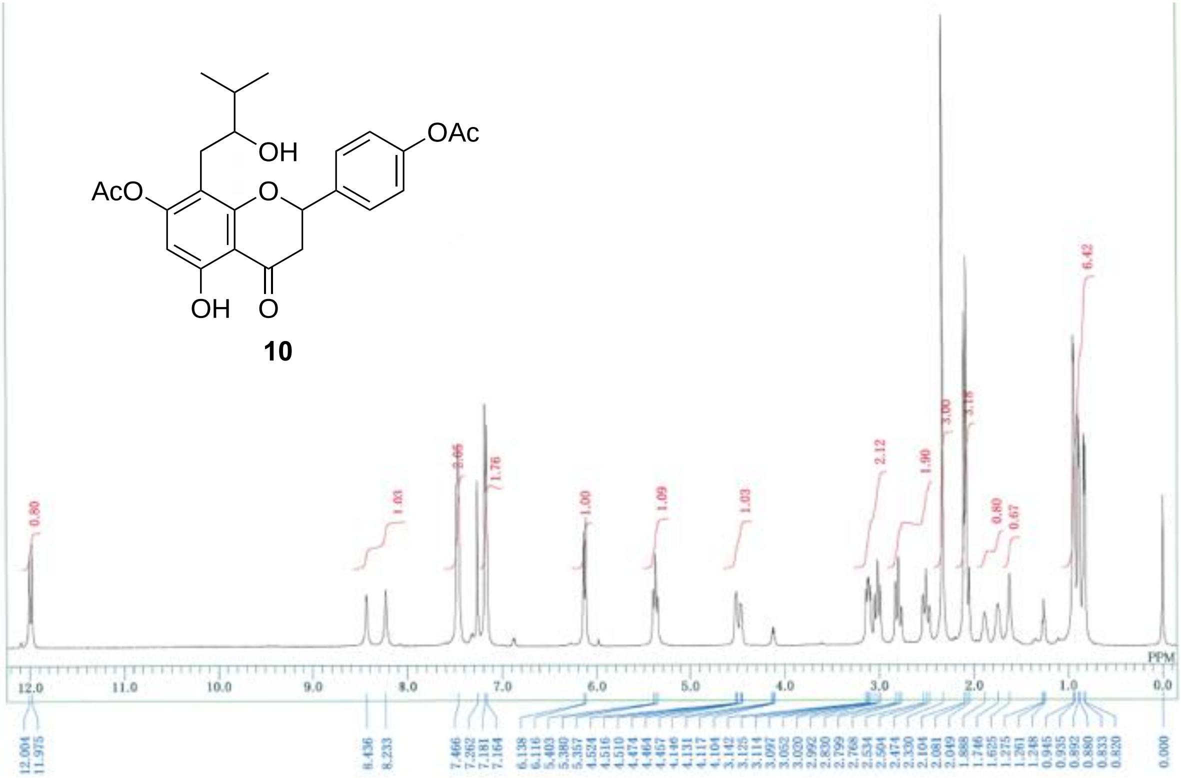

Figure S22. ^{13}C NMR chart of compound **10**. ($CDCl_3$, 125 MHz, internal standard: TMS)

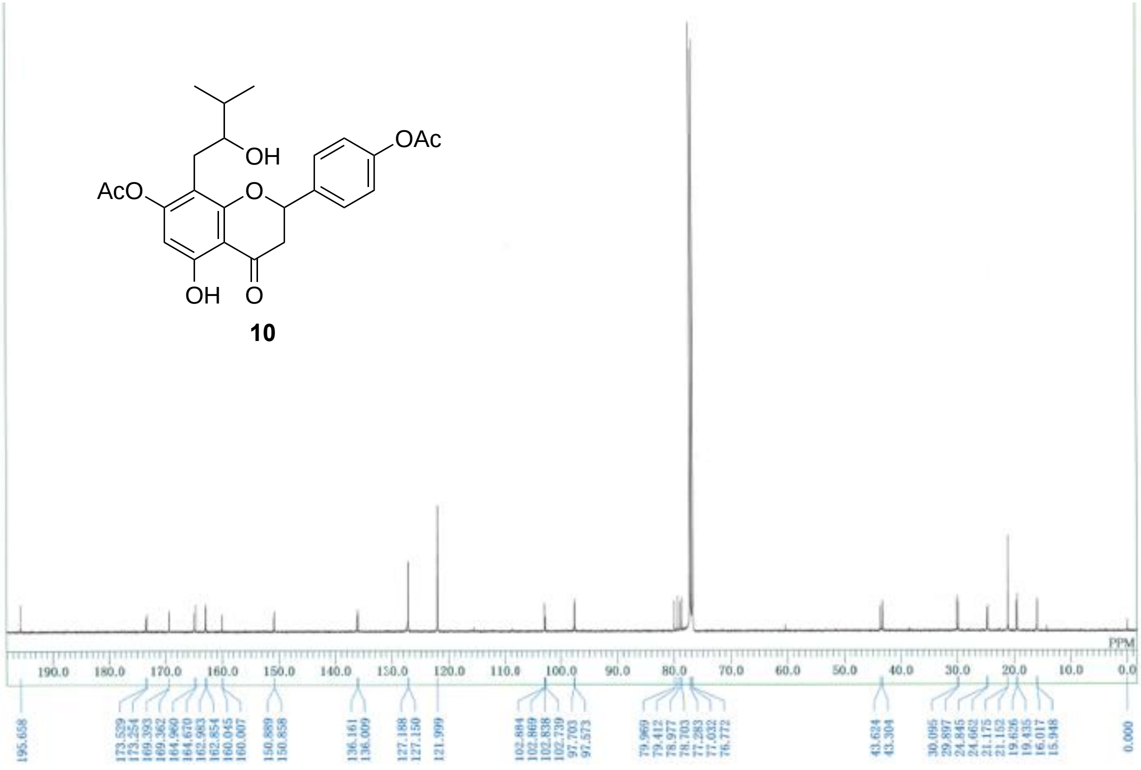

Figure S23. ESI-HR-MS chart of compound **10**. $[M+Na]^+$ m/z: Calcd for $C_{24}H_{26}O_8Na$ 465.1525; Found 465.1519.

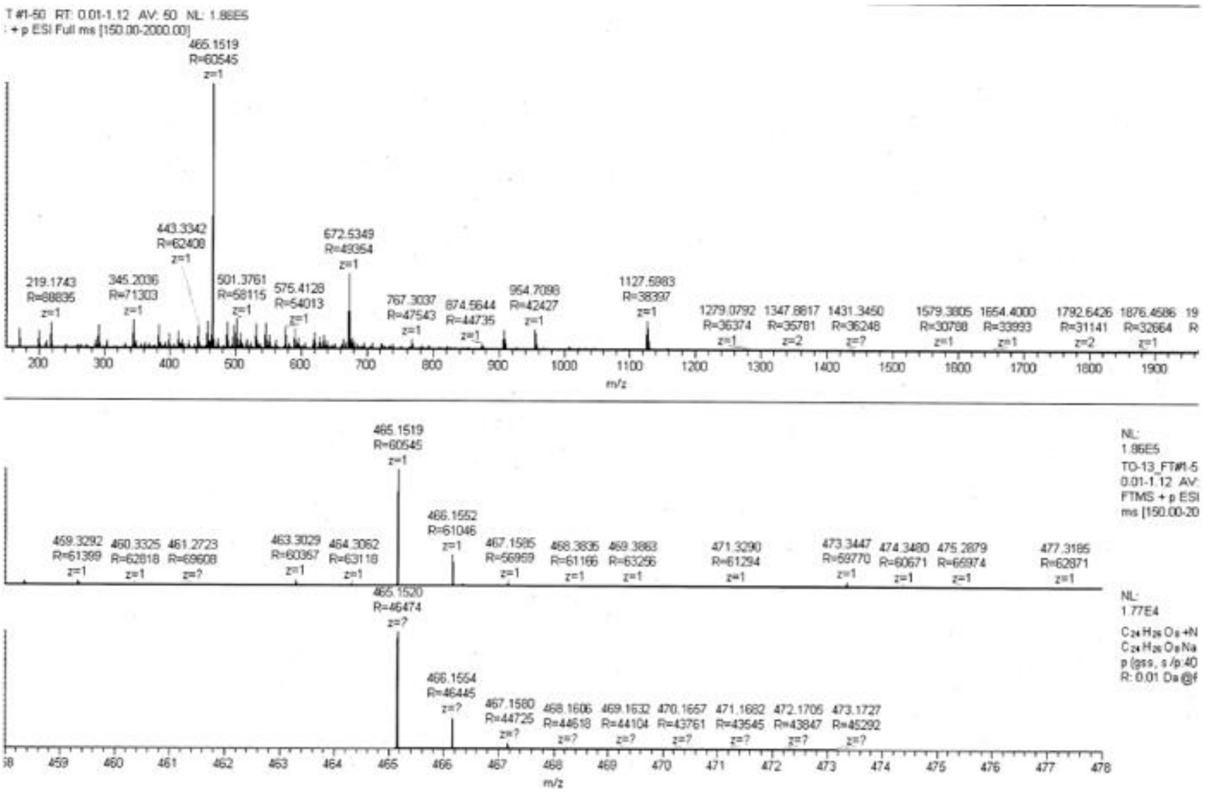

target m/z 465.1519

Calculated results

Idx.	Formula	RDB	Delta ppm
1	C24 H26 O8 Na	11.5	−0.213

Bounding conditions

Charge		1
Nitrogen Rule	None	
Mass tolerance		5 ppm
RDB equiv.		−1 _100

Elements in use

16 O	0	15	0	15.995
12 C	0	50	1	12
1 H	0	50	−0.5	1.008
23 Na	0	1	−0.5	22.99

Figure S24. ^{1}H NMR chart of compound **1e**. (DMSO-*d6*, 500 MHz)

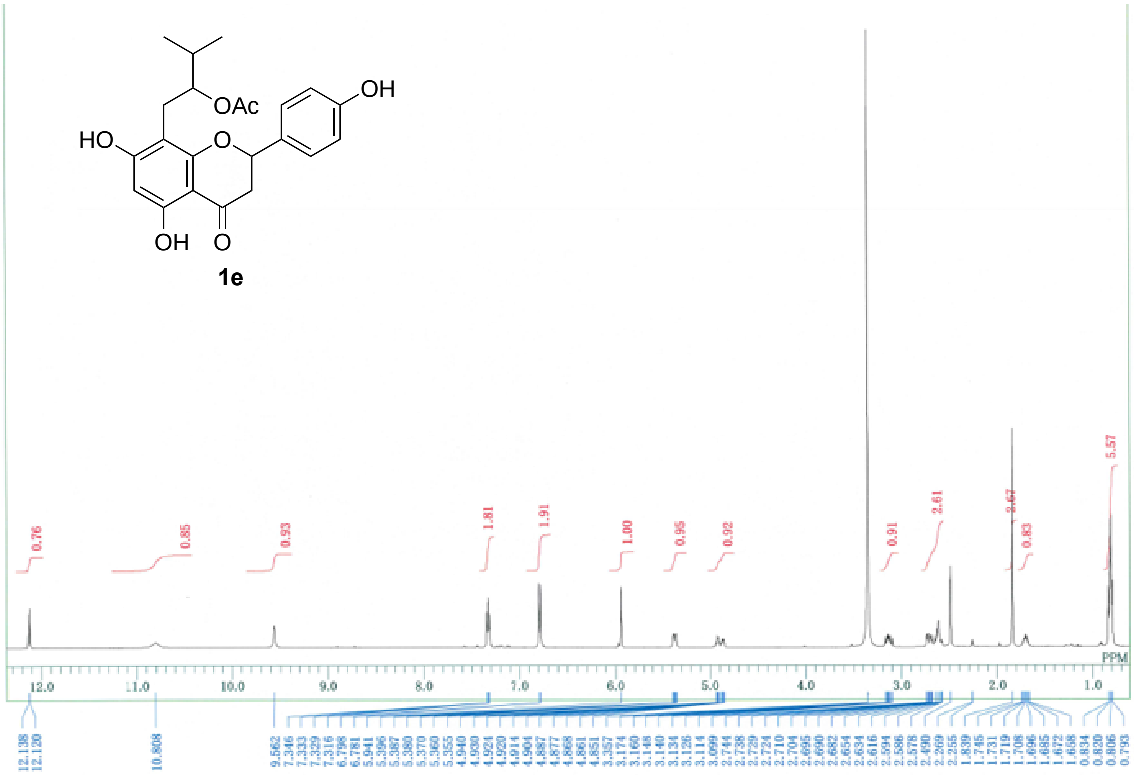

Figure S25. ^{13}C NMR chart of compound **1e**. (DMSO-*d6*, 125 MHz)

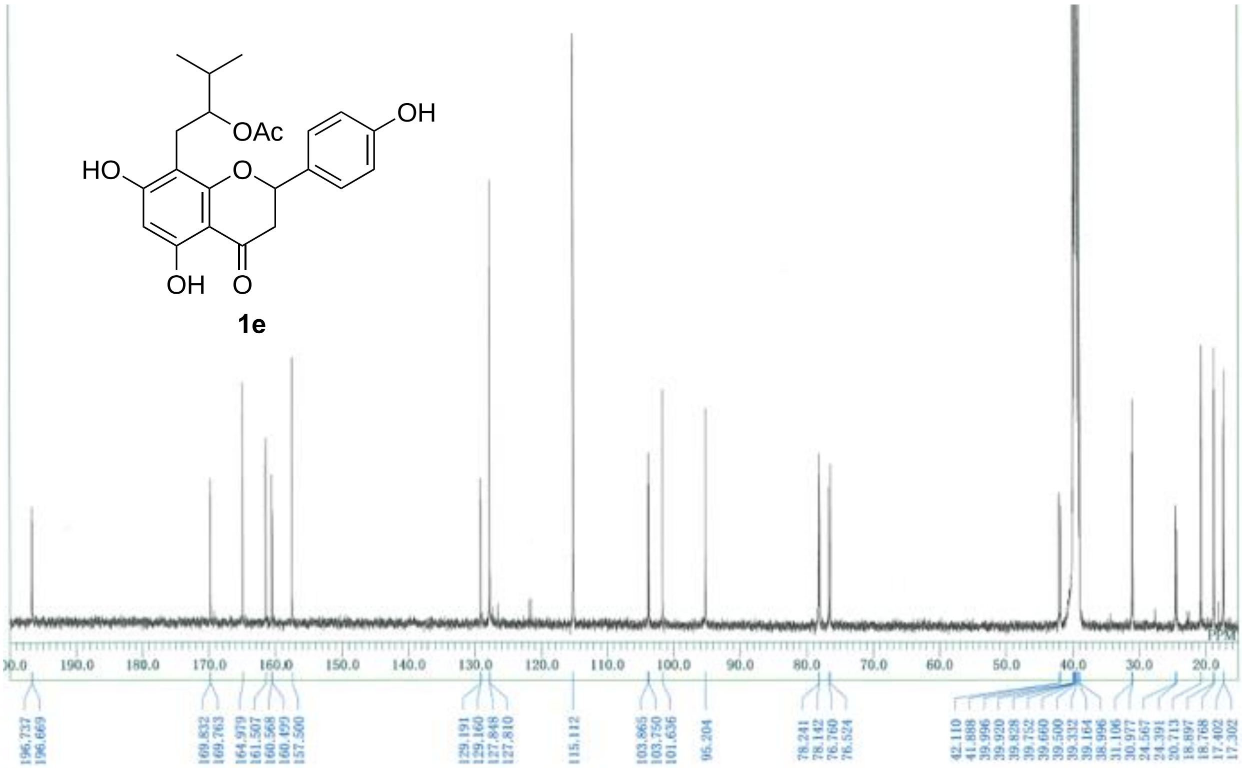

Figure S26. ESI-HR-MS chart of compound **10**. $[M+Na]^{+}$ m/z: Calcd for $C_{22}H_{24}O_{7}Na$ 423.1420; Found 423.1414.

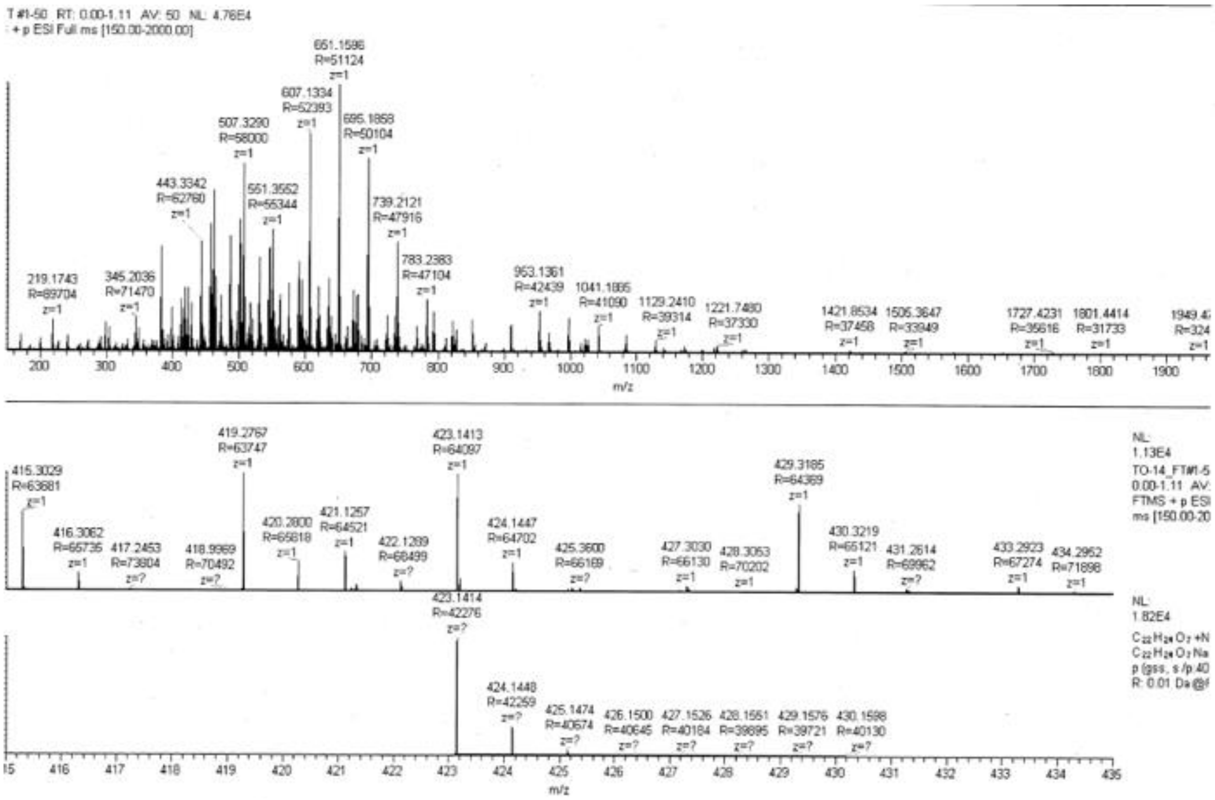

target m/z		423.1414		

Calculated results

Idx.	Formula	RDB	Delta ppm
1	C22 H24 O7 Na	10.5	−0.175

Bounding conditions

Charge		1
Nitrogen Rule	None	
Mass tolerance		5 ppm
RDB equiv.		−1 _100

Elements in use

16 O	0	15	0	15.995
12 C	0	50	1	12
1 H	0	50	−0.5	1.008
23 Na	0	1	−0.5	22.99

Figure S27. ¹H NMR chart of compound **2a**. (DMSO-*d6*, 500 MHz)

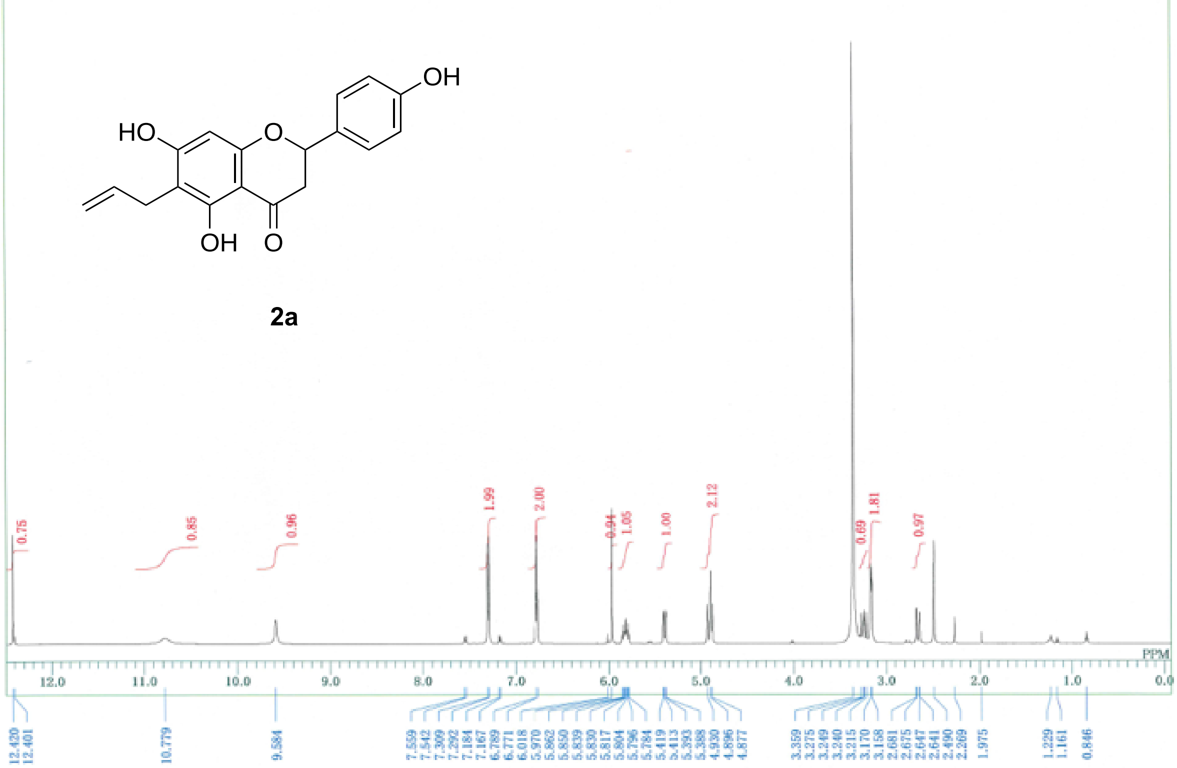

Figure S28. ¹³C NMR chart of compound **2a**. (DMSO-*d6*, 125 MHz)

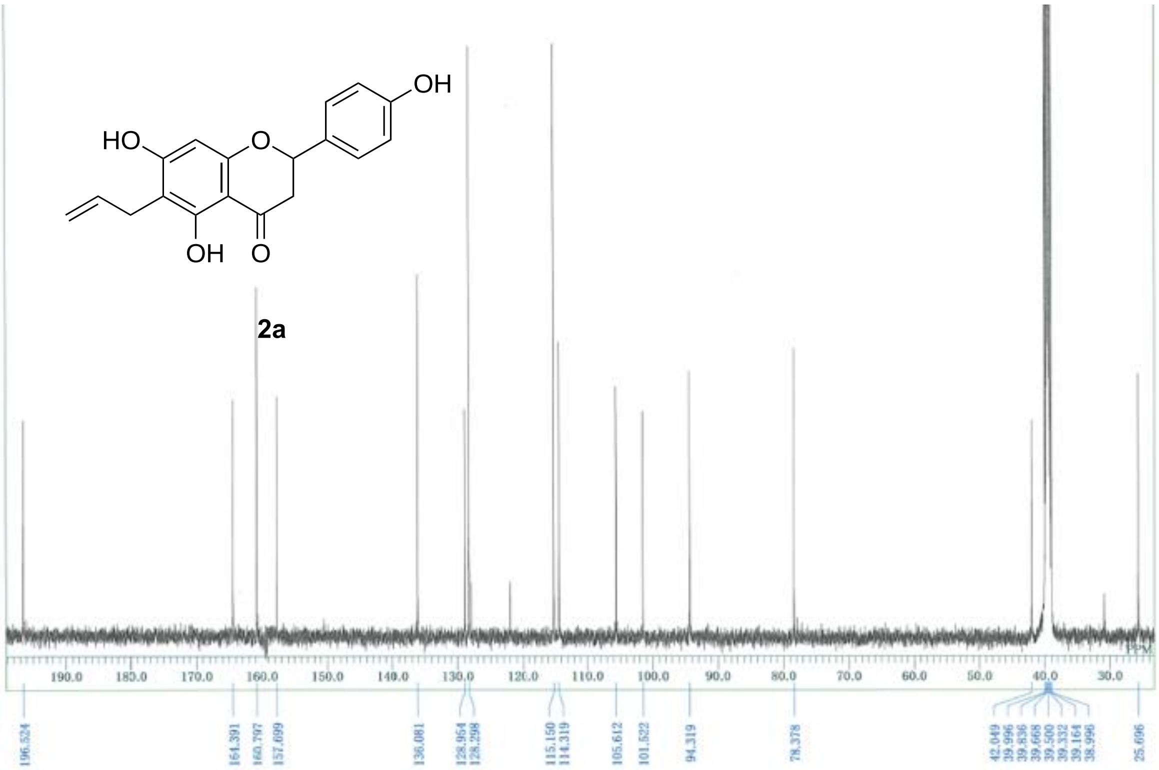

Figure S29a. ¹H NMR chart of compound **6-PN**. (DMSO-*d6*, 500 MHz)

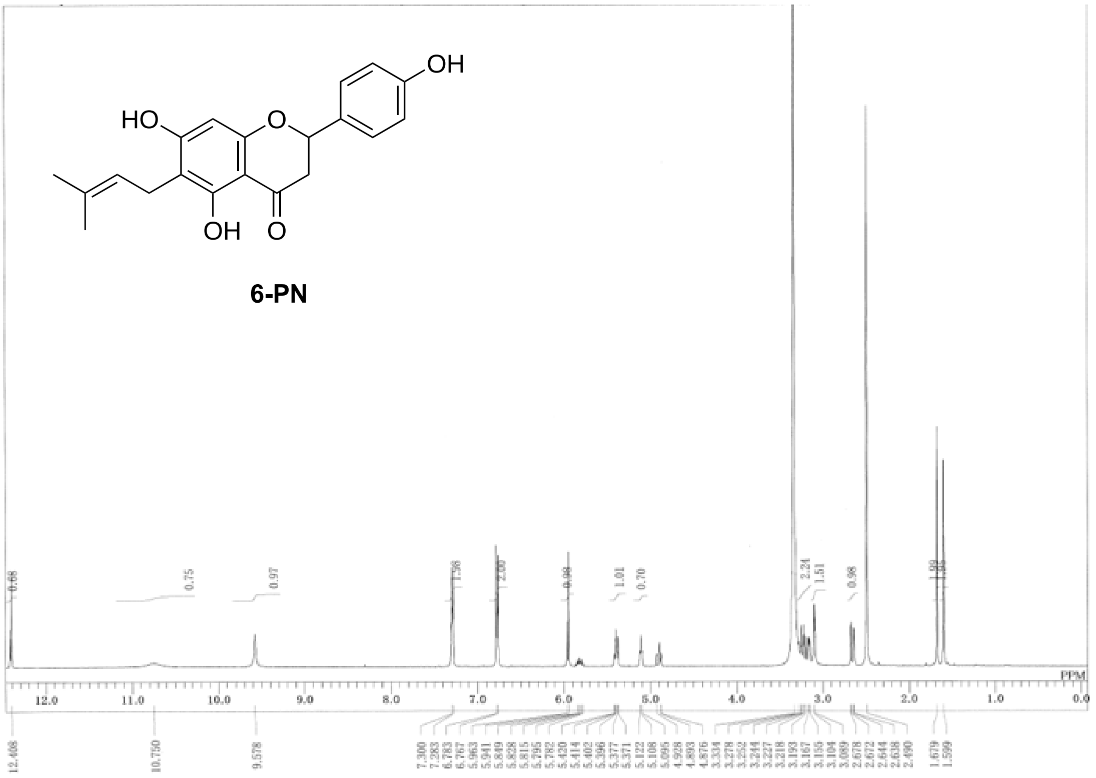

Figure S29b. ^{1}H NMR chart of compound **6-PN**. ($CDCl_3$, 500 MHz, internal standard: TMS)

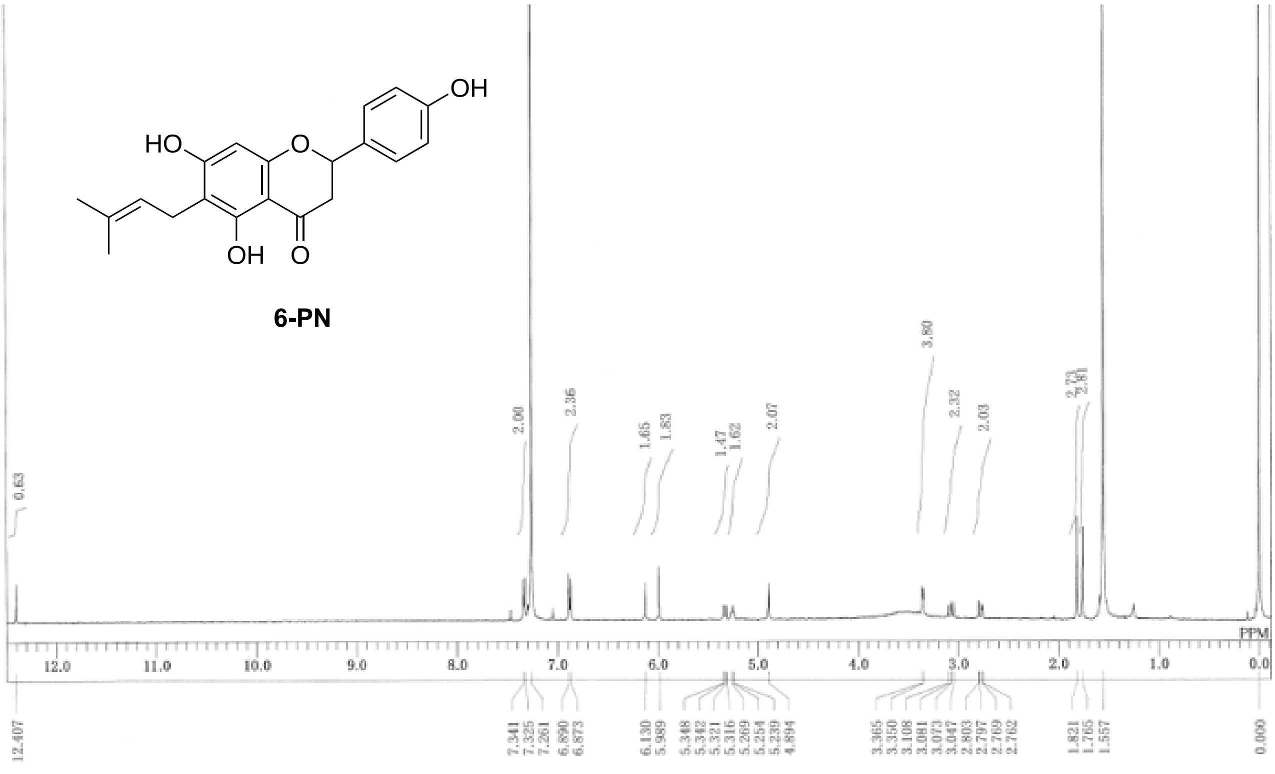

Figure S30. ^{1}H NMR chart of compound **12**. ($CDCl_3$, 500 MHz, internal standard: TMS)

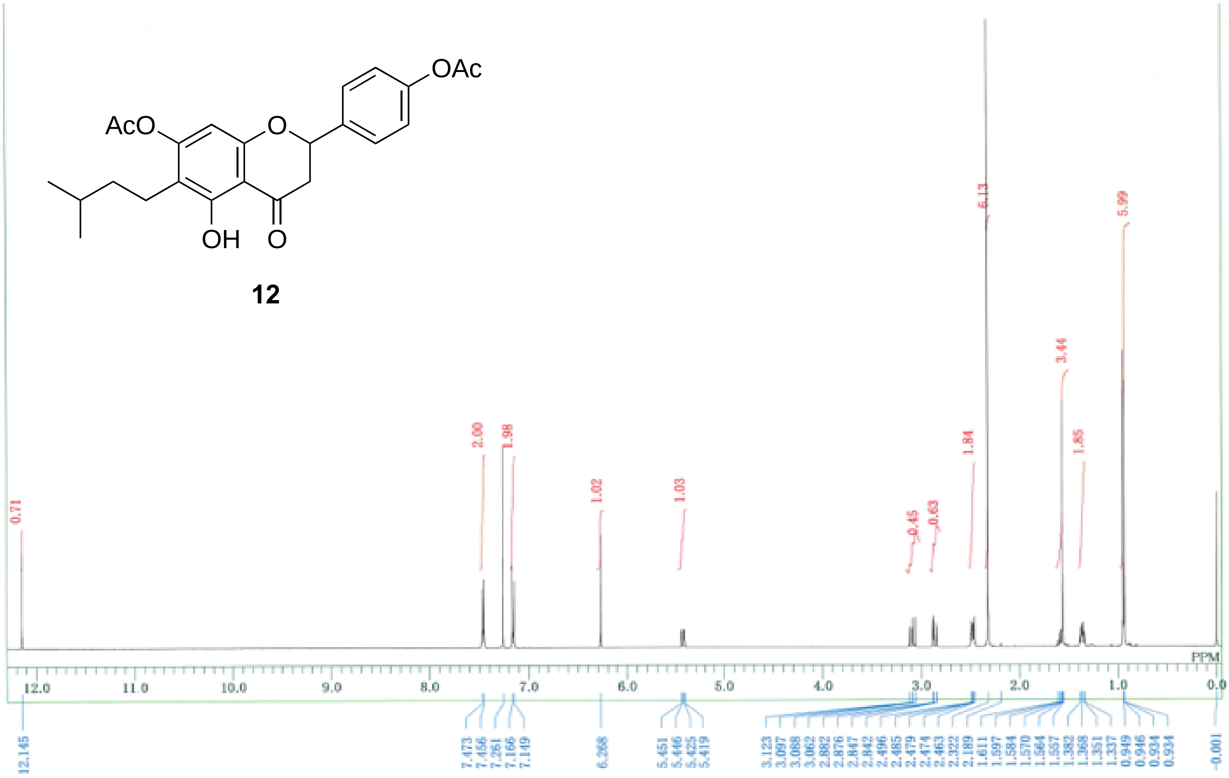

Figure S31. ^{1}H NMR chart of compound **2b**. (DMSO-*d6*, 500 MHz)

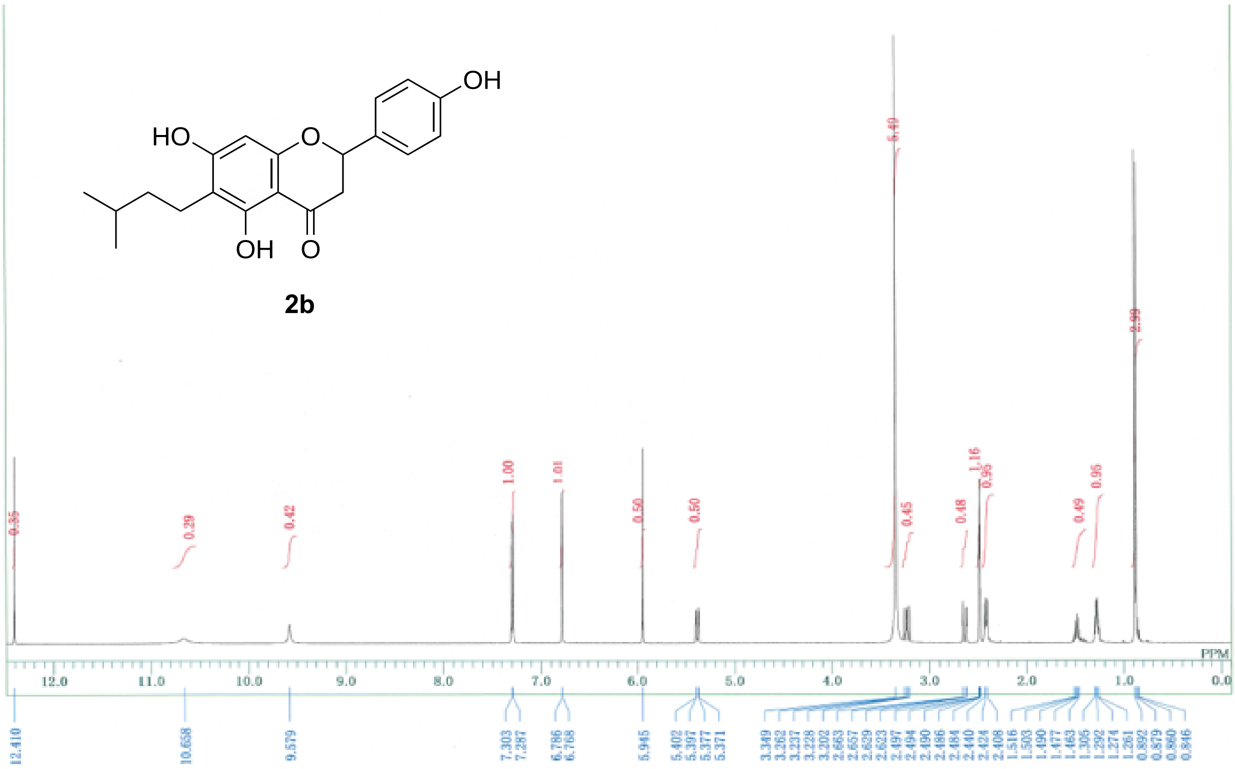

Figure S32. ^{1}H NMR chart of compound **13**. ($CDCl_3$, 500 MHz, internal standard: TMS)

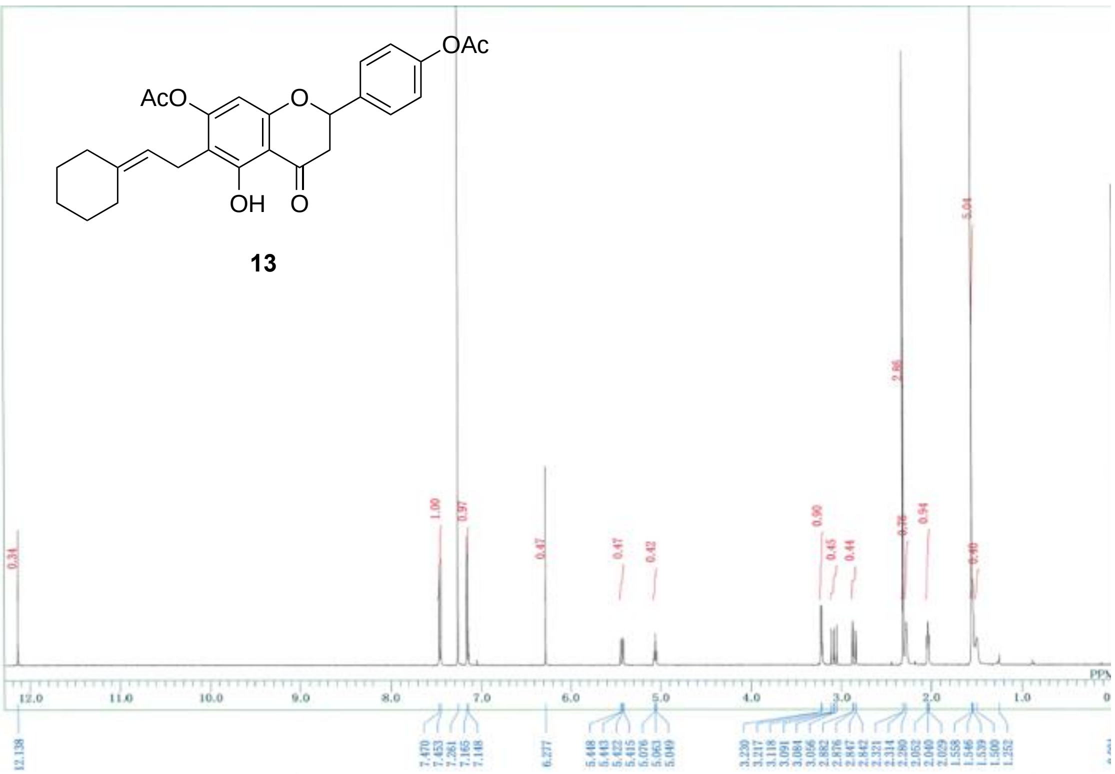

Figure S33. ^{13}C NMR chart of compound **13**. (DMSO-*d6*, 125 MHz)

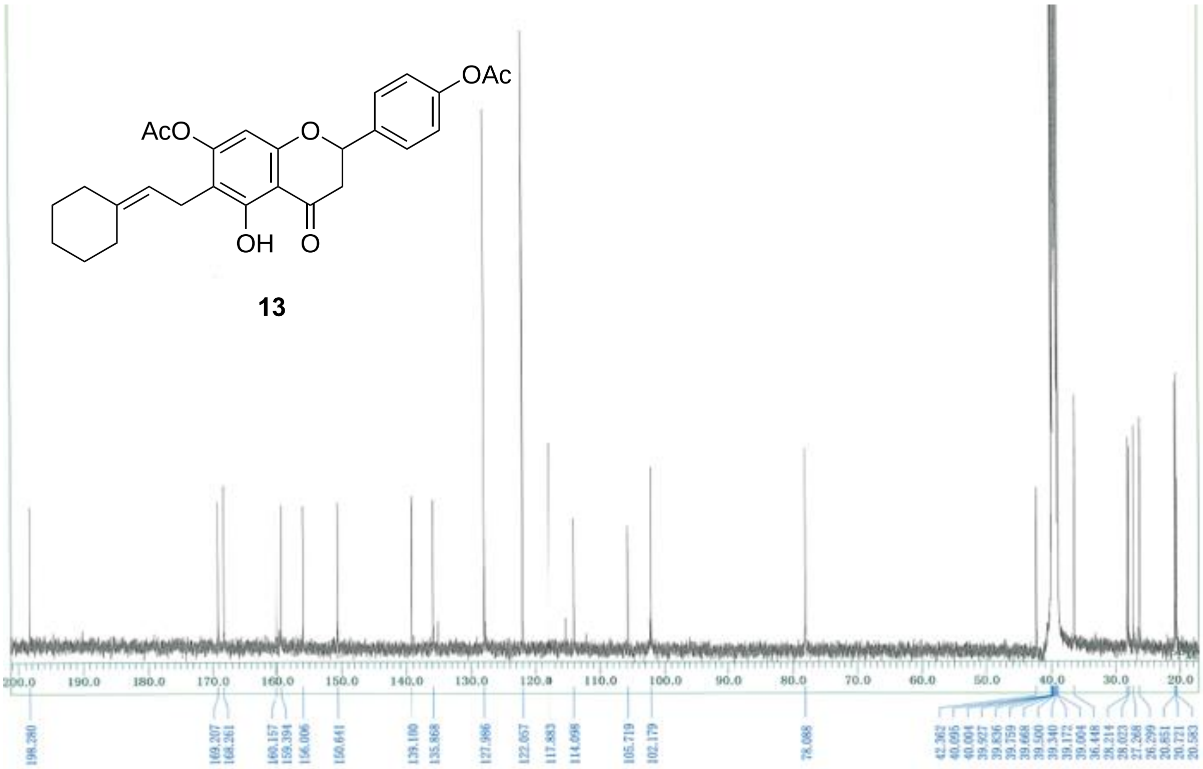

Figure S34. ESI-HR-MS chart of compound **13**. $[M+H]^+$ m/z: Calcd for $C_{27}H_{29}O_7$ 465.1908; Found 465.1915.

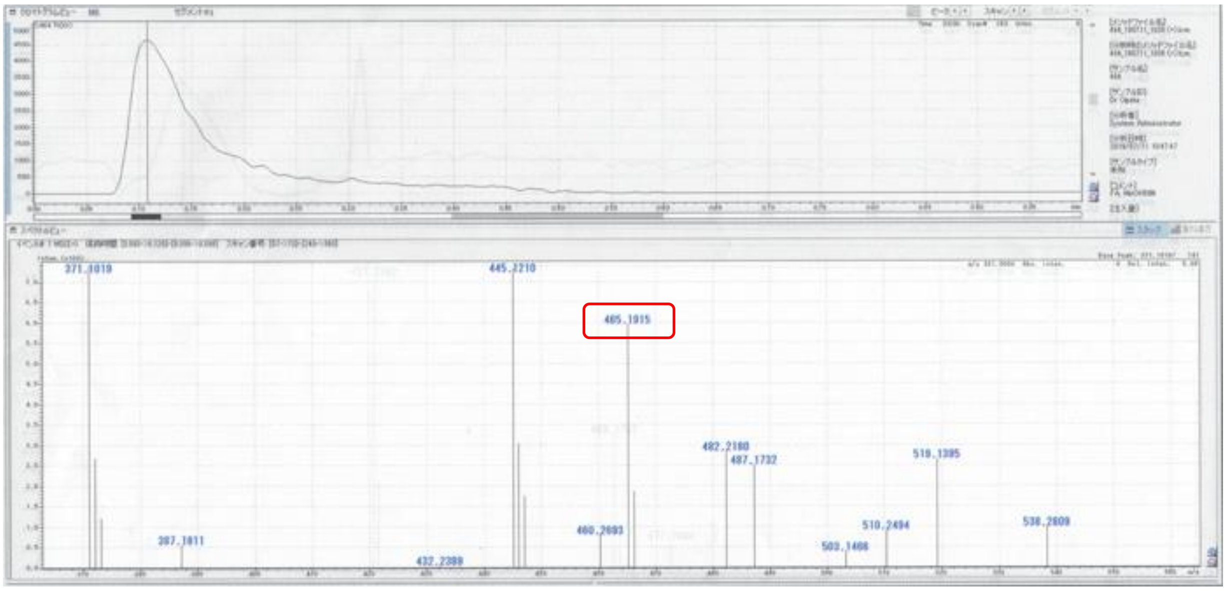
371.1019
445.2210
465.1915
482.2180
487.1732
519.1395
538.2609
510.2494
503.1466
460.2693
432.2399
397.1011

Figure S35. ^{1}H NMR chart of compound **2c**. (DMSO-*d6*, 500 MHz)

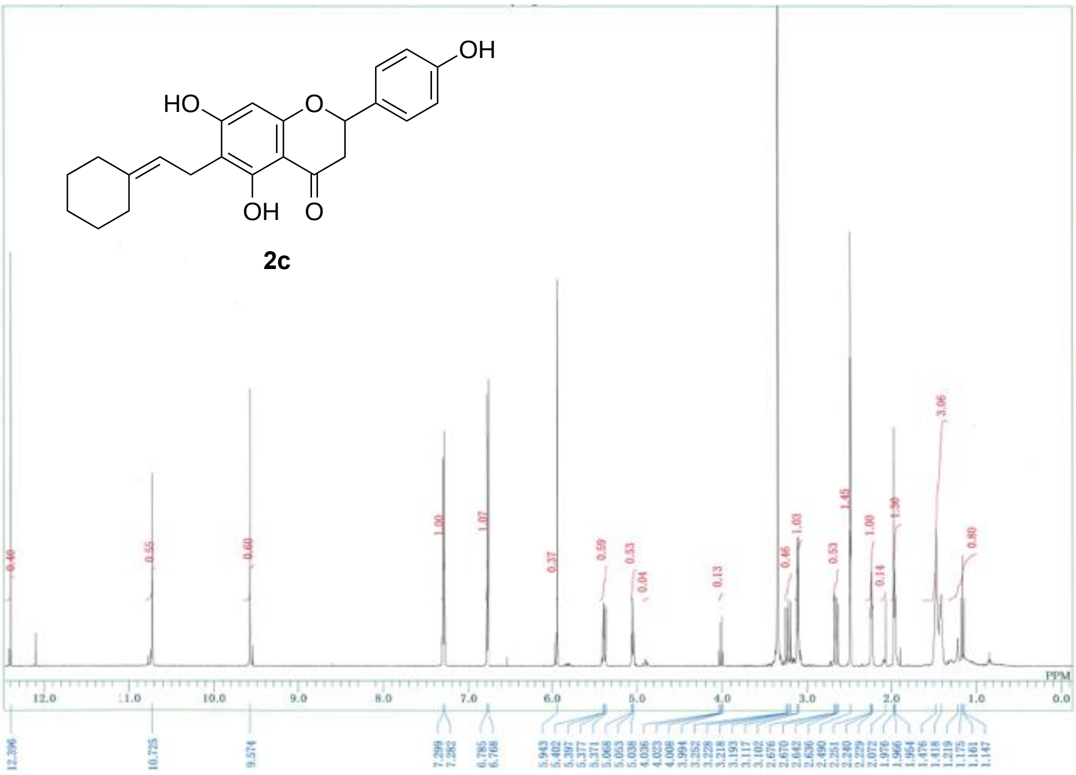

Figure S36. ^{13}C NMR chart of compound **2c**. (DMSO-*d6*, 125 MHz)

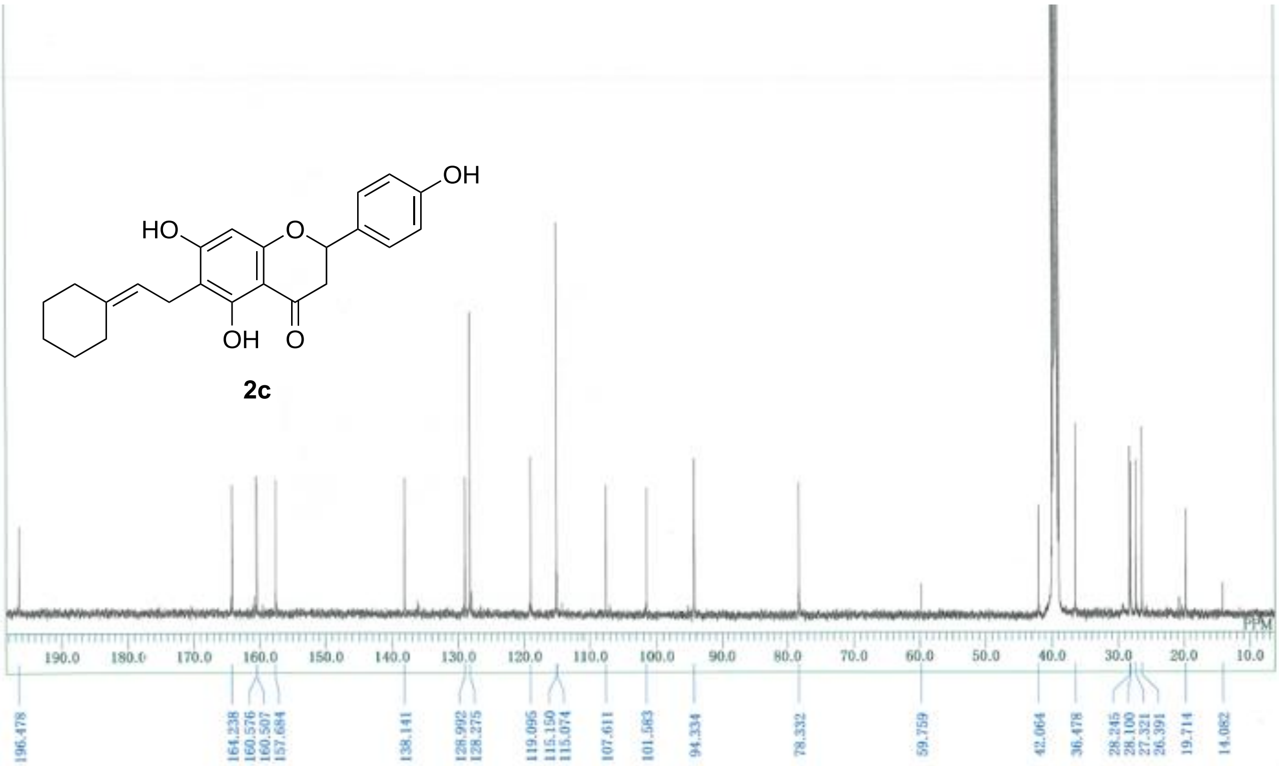

Figure S37. ESI-HR-MS chart of compound **2c**. $[M+H]^+$ m/z: Calcd for $C_{23}H_{23}O_5$ 379.1551; Found 379.1560.

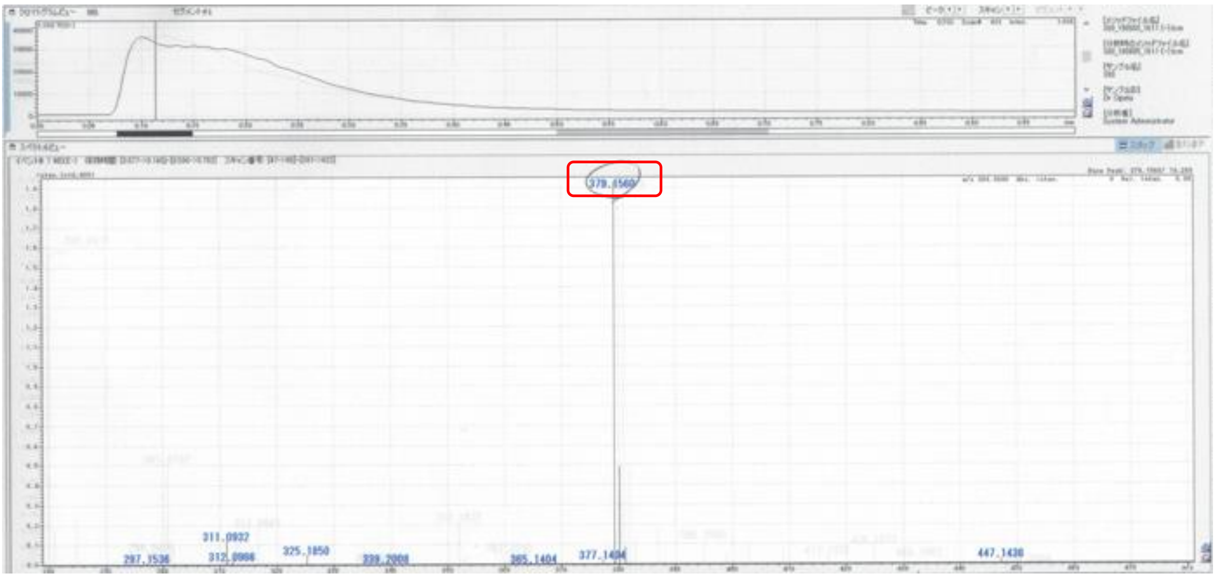

Figure S38. ^{1}H NMR chart of compound **14**. ($CDCl_3$, 500 MHz, internal standard: TMS)

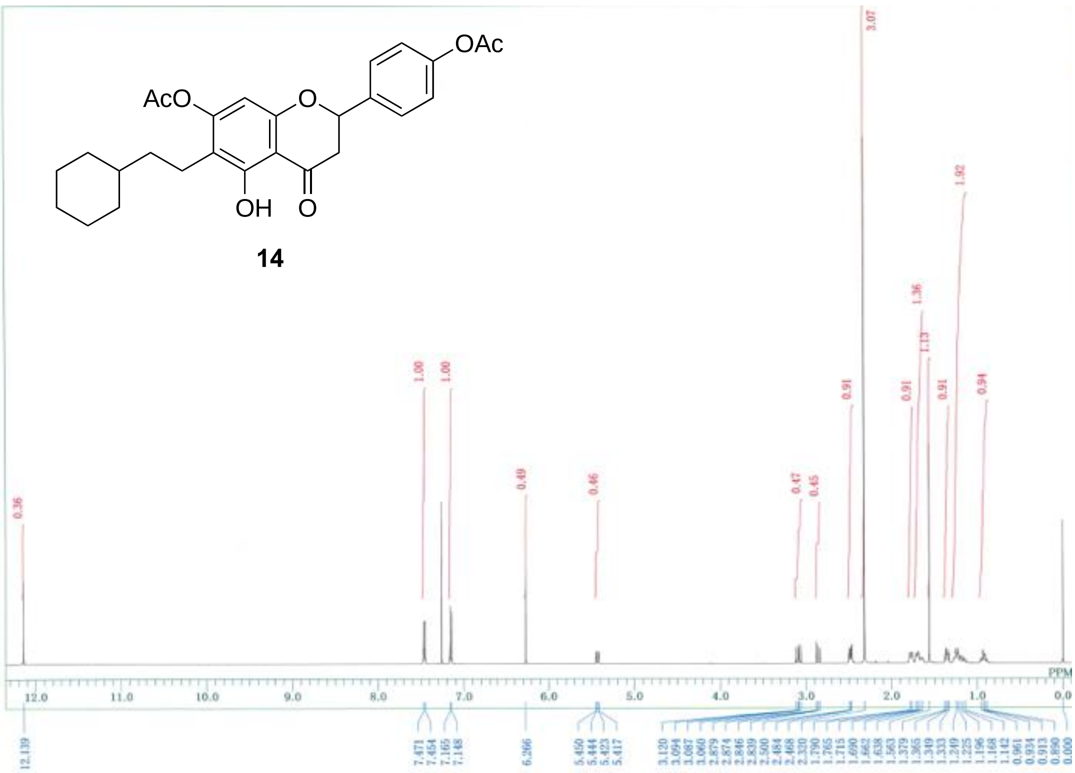

Figure S39. ^{13}C NMR chart of compound **14**. (DMSO-*d6*, 125 MHz)

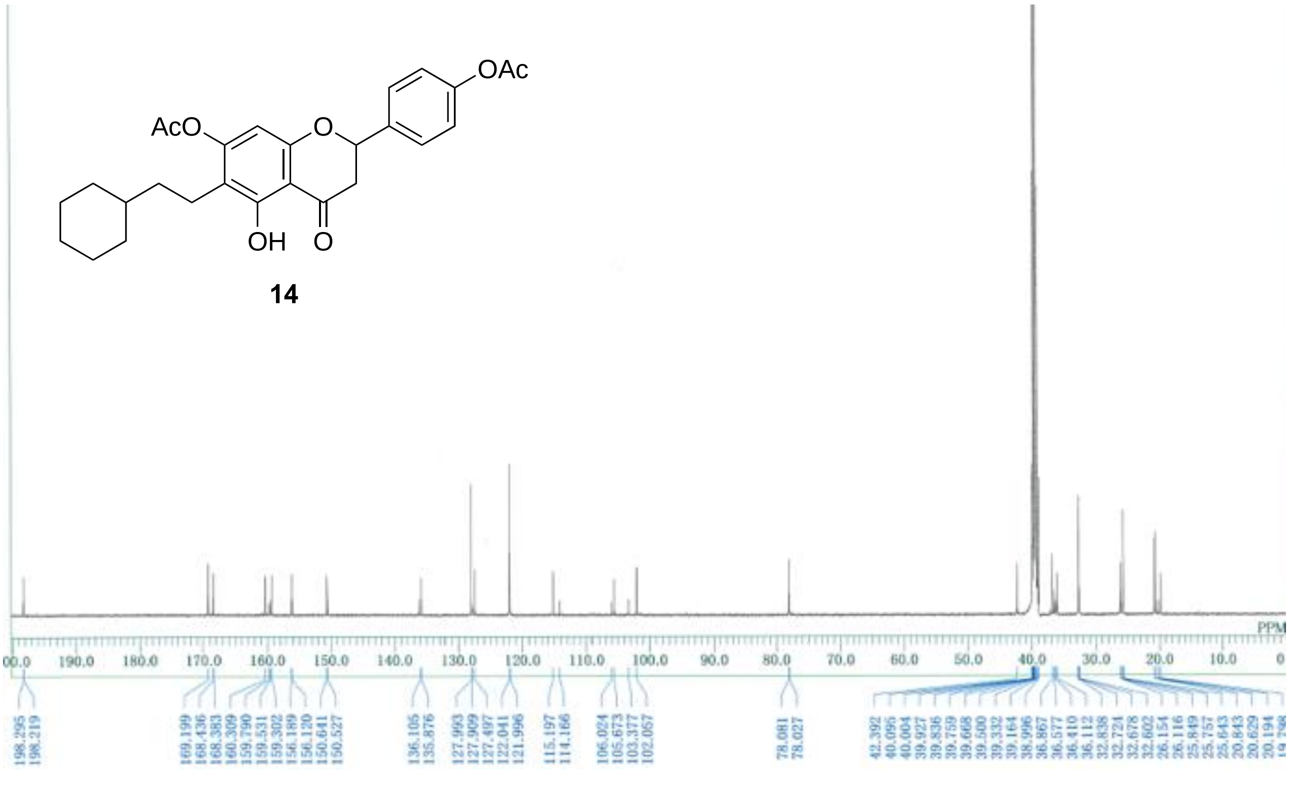

Figure S40. HR-MS chart of compound **14**. $[M+H]^+$ m/z: Calcd for $C_{27}H_{31}O_7$ 467.2064; Found 467.2069.

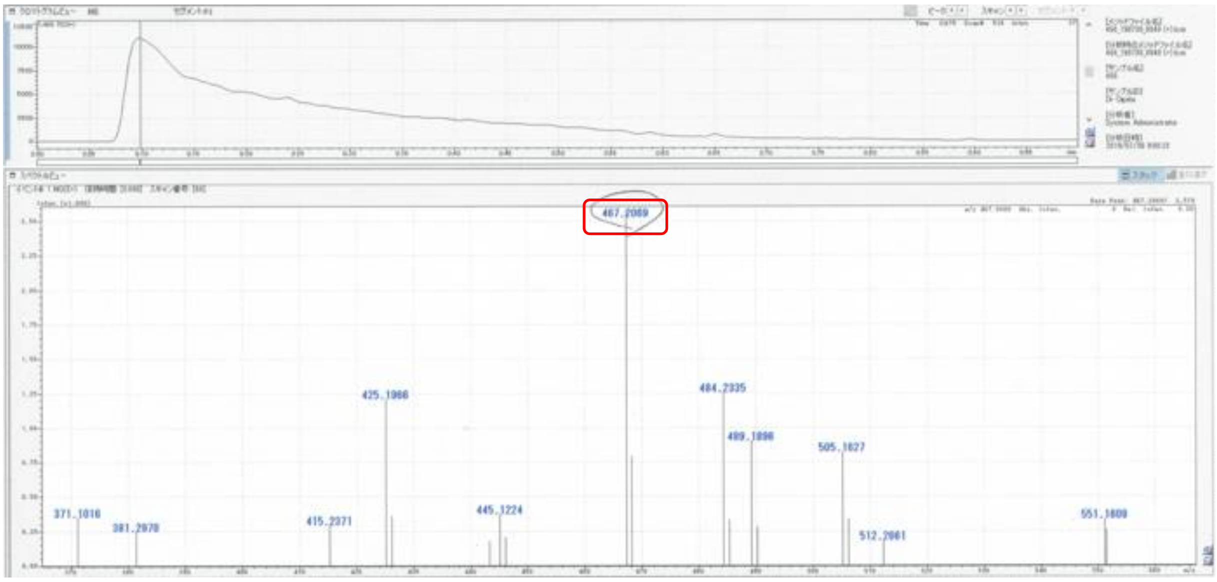

Figure S41. ^{1}H NMR chart of compound **2d**. (DMSO-*d6*, 500 MHz)

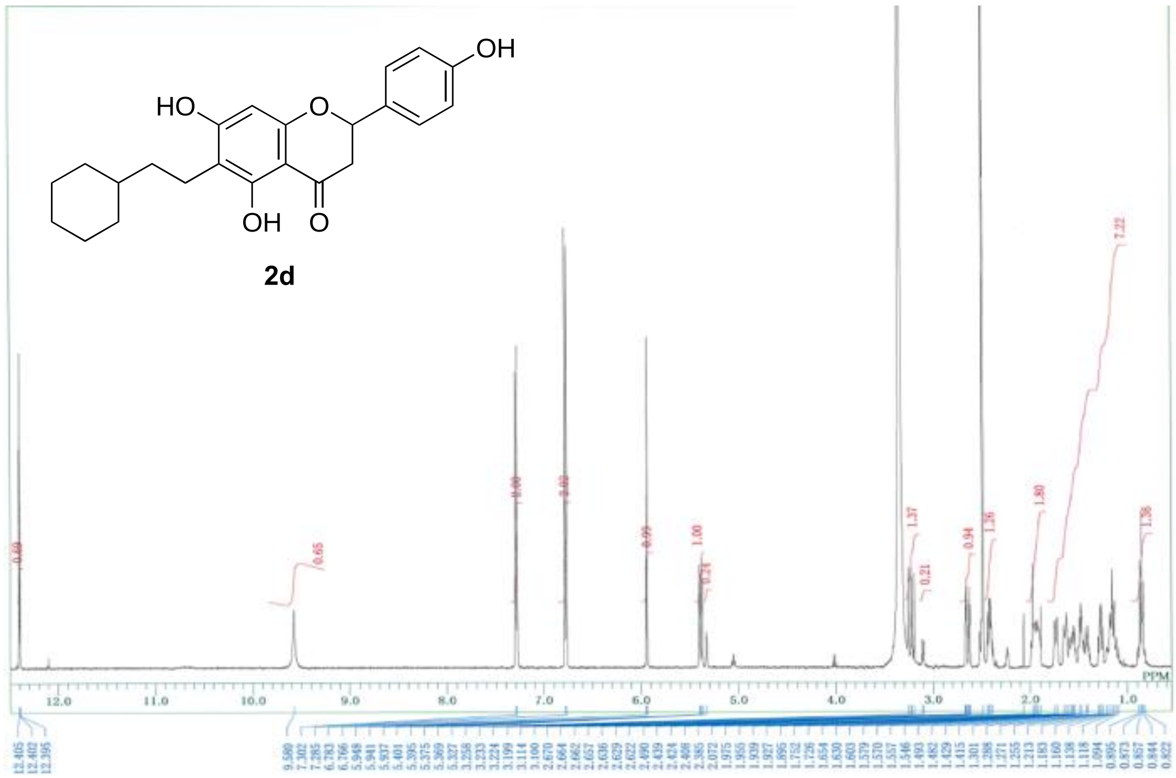

Figure S42. ^{13}C NMR chart of compound **2d**. (DMSO-*d6*, 125 MHz)

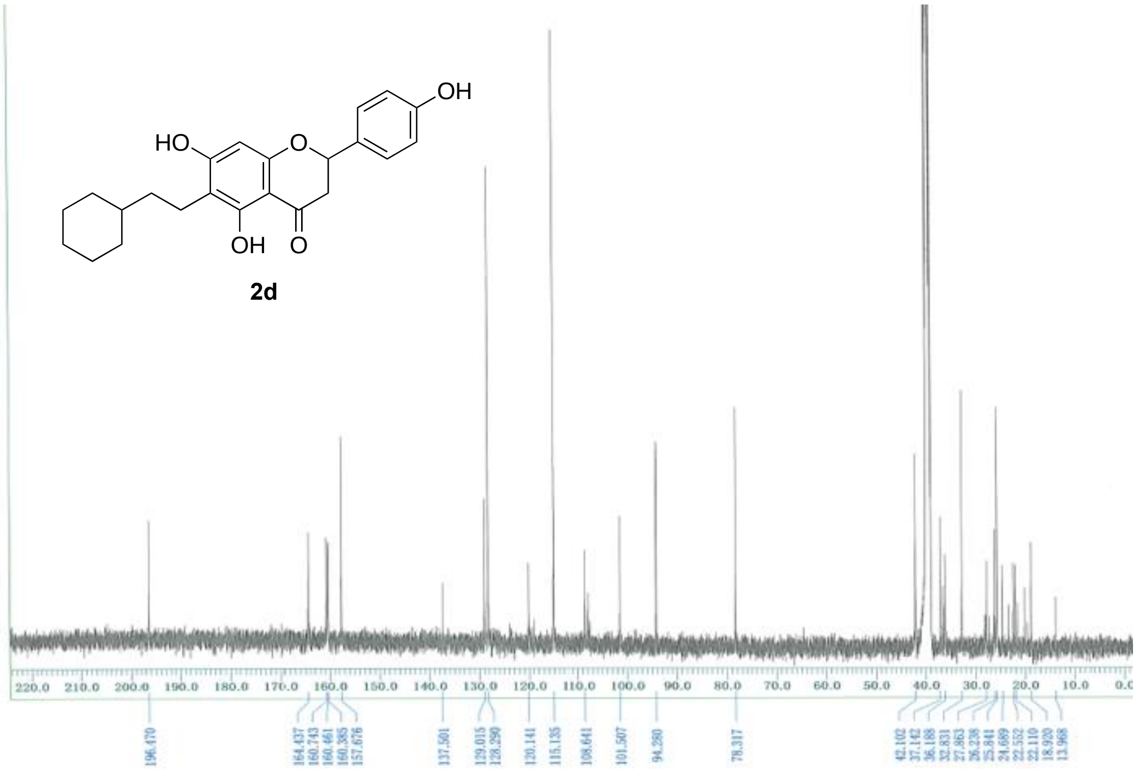

Figure S43. ESI-HR-MS chart of compound **2d**. $[M+H]^+$ m/z: Calcd for $C_{23}H_{25}O_5$ 381.1707; Found 381.1715.

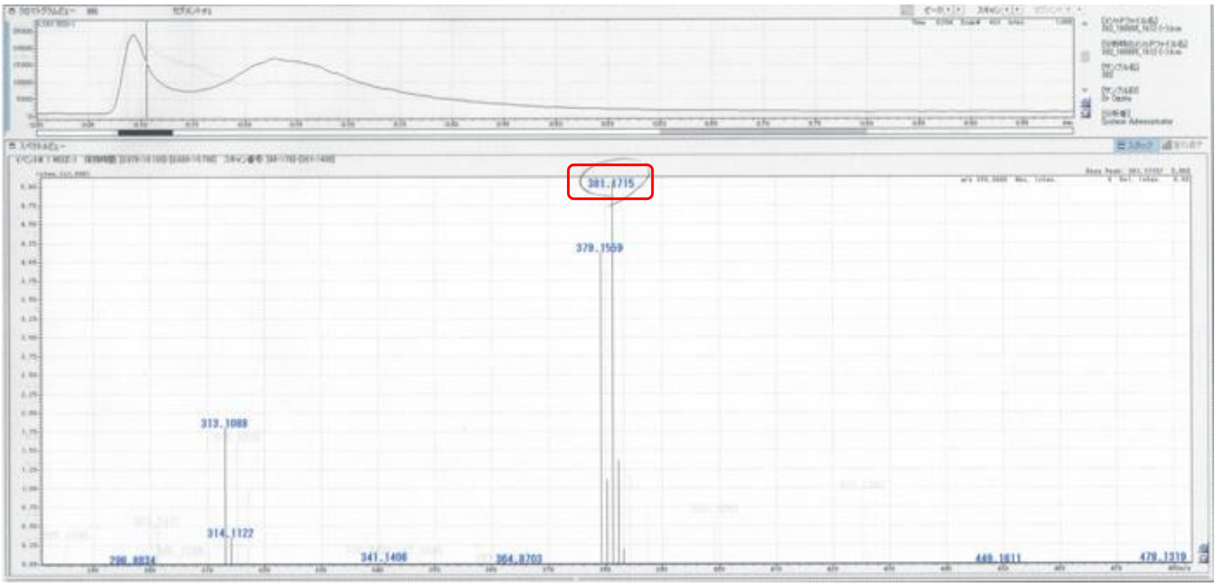

References

1. Gester, S.; Metz, P.; Zierau, O.; Vollmer, G. An efficient synthesis of the potent phytoestrogens 8-prenylnaringenin and 6-(1,1-Dimethylallyl) naringenin by europium (III)-catalyzed claisen rearrangement. Tetrahedron. **2001**, 57, 1015–1018. [PubMed] https://doi.org/10.1016/S0040-4020(00)01078-4
2. Tischer, S.; Metz, P. Selective C-6 prenylation of flavonoids via europiumA (III)-catalyzed claisen rearrangement and cross-metathesis. Adv. Synth. Catal. **2007**, 349, 147-51. [PubMed] https://doi.org/10.1002/adsc.200600454.
3. Ogata, T.; Tanaka, M.; Ishigaki, M.; Shimizu, M.; Nishiuchi, A.; Inamoto, K.; Kimachi, T. The first enantioselective total synthesis of lantalucratin C and determination of its absolute configuration. Tetrahedron. **2015**, 71, 6672-6680. [PubMed] https://doi.org/10.1016/j.tet.2015.07.050.
4. Su, W.; Wang, Y. PCT Int. Appl. WO 2014094370 A1 20140626, **2014**.
5. Laettig, J.; Boehl, M.; Fischer, P.; Tischer, S.; Tietboehl, C.; Menschikowski, M.; Gutzeit, H.O.; Metz, P.; Pisabarro, M.T.; Comput, J. Mechanism of inhibition of human secretory phospholipase A2 by flavonoids: rationale for lead design. J. Comput. Aided. Mol. Des. **2007**, 21, 473-483. [PubMed] https://doi.org/10.1007/s10822-007-9129-8.
6. Hänsel, R.; Schulz, J. Desmethylxanthohumol: Isolierung aus hopfen und cyclisierung zu flavanonen. Arch. Pharm. **1988**, 321, 37–40.
7. Kumano, T.; Richard, S.B.; Noel, J. P.; Nishiyama, M.; Kuzuyama, T. Chemoenzymatic synthesis of

prenylated aromatic small molecules using streptomyces prenyltransferases with relaxed substrate specificities. Bioorg. Med. Chem. **2008**, 16, 8117-8126.

8. Nguyen, H Du.; Okada, T.; Kitamura, S.; Yamaoka, S.; Horaguchi, Y.; Kasanami, Y.; Sekiguchi, F.; Tsubota, M.; Yoshida, S.; Nishikawa, H.; Kawabata, A.; Toyooka, N. Design and synthesis of novel anti-hyperalgesic agents based on 6-prenylnaringenin as the T-type calcium channel blockers. Bioorg. Med. Chem. **2018**, 26, 4410–4427. [PubMed] https://doi.org/ 10.1016/j.bmc.2018.07.023.
